# A PSMA-targeted dextran-based conjugate eradicates PSMA-overexpressing prostate tumors while abolishing cabazitaxel toxicity

**DOI:** 10.7150/thno.124663

**Published:** 2026-05-11

**Authors:** Li Yang, Tingchao Liu, Meng Huo, Weiping Jia, Yu Chen, Maoxu Ge, Yan Pan, Jianfei Shi, Xiaohai Li, Si Wang, Yikang Shi

**Affiliations:** 1 National Glycoengineering Research Center, Shandong University, Qingdao 266237, China; Shandong Center of Technology Innovation for Carbohydrate, Shandong University, Qingdao 266237, China; Shandong Key Laboratory of Carbohydrate and Carbohydrate-conjugate Drugs, Shandong University, Qingdao 266237, China.; 2 Department of Pharmacy, Qilu Hospital of Shandong University, Jinan 250012, China.; 3 Department of Pharmacology and Pharmaceutical Sciences, Alfred E. Mann School of Pharmacy and Pharmaceutical Sciences, University of Southern California, Los Angeles, CA 90089, United States.; 4 Santolecan Pharmaceuticals LLC, Jupiter 33458, United States.; 5 Suzhou Santolecan Pharmaceutical Co., Ltd, Suzhou 215123, China.

**Keywords:** prostate cancer, cabazitaxel, dextran, gamma-linolenic acid, prostate-specific membrane antigen

## Abstract

**Background:**

High expression of prostate-specific membrane antigen (PSMA) is observed in advanced prostate cancer, supplying a promising target for precision therapeutic interventions. Despite its efficacy in metastatic castration-resistant disease, cabazitaxel (CTX) is limited by severe systemic toxicity and a narrow therapeutic index, underscoring the urgent demand for tumor-selective delivery systems.

**Methods:**

A novel PSMA-targeted dextran-based conjugate, Dextran-CTX-GLA-EuK, was synthesized via click chemistry by conjugating CTX, γ-linolenic acid (GLA), and a Glu-urea-Lys (EuK) PSMA-targeting ligand to bifunctionalized dextran. Critical *in vitro* and *in vivo* PSMA blocking experiments (using the PSMA inhibitor 2-PMPA) were performed to validate its targeting specificity. A thorough myelosuppression study was performed in murine models to evaluate the systemic hematological safety profile. The biodistribution profile and *in vivo* antitumor efficacy of the conjugate were evaluated in murine xenograft models.

**Results:**

The conjugate Dextran-CTX-GLA-EuK exhibited favorable physicochemical properties, high water solubility, and strong PSMA-binding affinity. *In vitro* and *in vivo* PSMA blocking experiments conclusively verifying its PSMA-mediated specific cellular internalization and tumor accumulation. In PSMA-overexpressing xenograft models, the conjugate demonstrated selective tumor enrichment, with intratumoral CTX levels up to 98.3-fold higher than those of parent CTX, while reducing exposure in normal tissues. Dextran-CTX-GLA-EuK exerted prominent dose-dependent tumor growth inhibition, attaining a 96.6% suppression rate at a 10 mg/kg dosage in PC-3/PSMA tumors and prolonging the survival of 22Rv1 tumor-bearing mice. Importantly, comprehensive myelosuppression assays revealed that the conjugate only induced a transient reduction in white blood cell and neutrophil counts (which rapidly recovered to baseline) without impairing bone marrow hematopoietic function; unlike CTX, the conjugate did not cause significant weight loss, organ toxicity, or hematological abnormalities in tumor-bearing mice.

**Conclusions:**

These findings demonstrate that Dextran-CTX-GLA-EuK synergizes active targeting with dextran-based delivery, enhancing antitumor efficacy while abolishing dose-limiting toxicities. This strategy offers a clinically translatable approach for PSMA-directed therapy in prostate cancer.

## Introduction

Prostate cancer (PCa) ranks as the second most frequently diagnosed male malignancy globally, with approximately 1.4 million new cases and over 375,000 annual deaths reported 1. While early-stage PCa is often curable via radical prostatectomy or radiotherapy, a significant number of patients eventually develop advanced or metastatic castration-resistant prostate cancer (mCRPC)—a fatal, treatment-refractory stage characterized by resistance to androgen deprivation therapy (ADT). Despite advances in systemic therapies, approximately half of these patients die within two years of diagnosis, and the overall five-year survival rate in the United States for histologically confirmed metastatic adenocarcinoma of the prostate remains only 10–31% across different series 2. This grim prognosis highlights the urgent need for more effective and tumor-selective therapeutic strategies.

ADT remains the cornerstone of PCa treatment, often combined with androgen receptor signaling inhibitors, taxane-based chemotherapies, or radioligand therapies. In mCRPC, cabazitaxel (CTX)—a second-generation taxane—has become a key therapeutic option following docetaxel failure. Cabazitaxel exerts its antitumor effects by interfering with microtubule dynamics, blocking cancer cell division and promoting apoptosis, which in turn extends survival and slows disease progression in this challenging patient population. Nevertheless, its clinical application is markedly limited by dose-limiting toxicities, such as neutropenia and gastrointestinal side effects, as well as by limited tumor specificity 3, 4. This constrained therapeutic index highlights the critical demand for approaches that can retain cabazitaxel’s efficacy while reducing systemic toxicity. Although novel formulations—including albumin-bound nanoparticles 5, 6, liposomes 7, 8, and polymer–drug conjugates 9, 10—have been investigated to enhance tumor targeting, none have fully overcome these limitations 11, 12. Therefore, there is a critical unmet need for advanced delivery platforms capable of selectively delivering cabazitaxel to tumor sites, improving its therapeutic index, and expanding its clinical utility in mCRPC.

A highly promising approach in PCa therapeutics is the targeting of PSMA, a type II transmembrane glycoprotein whose expression in mCRPC is 100 to 1000-fold higher relative to normal prostate tissues 13, 14. PSMA exhibits minimal expression in normal tissues, such as the kidney, salivary glands, and small intestine, rendering it a compelling target for both diagnostic imaging and targeted therapeutic precision interventions. Elevated PSMA levels in PCa are strongly correlated with tumor grade, metastatic potential, treatment resistance, and poor prognosis 13. The clinical utility of PSMA has been validated through the development of both diagnostic agents and therapeutics. The transmembrane characteristics of PSMA and its capacity for ligand-induced internalization allow for the targeted delivery of radionuclides (e.g., 177Lu) or cytotoxic agents to tumor cells. The US FDA has approved three PSMA-targeted diagnostics including ⁶⁸Ga-PSMA-11, ¹⁸F-DCFPyL, and ¹⁸F-Flotufolastat, which achieve superior detection sensitivity compared to conventional imaging 15, 16. In 2022, the FDA granted approval for PSMA-targeted theranostic ^177^Lu-PSMA-617 for patients with PSMA-PET-positive mCRPC who had previously progressed on at least one androgen receptor pathway inhibitor and one taxane-based chemotherapy. However, PSMA-directed radioligand therapy faces significant challenges such as PSMA heterogeneity, hematological toxicity and neutropenia 17-19. These limitations have spurred development of non-radioactive PSMA-targeted drug conjugates. Four antibody-drug conjugates (ADCs) targeting PSMA, including MLN2704, PSMA ADC, MEDI3726, and ARX517, have been evaluated in clinical trials. However, three of the four PSMA ADCs, although showing initial efficacy signals, these agents failed to deliver meaningful therapeutic benefits in clinical trials, primarily due to issues such as linker instability, rapid deconjugation, poor safety profiles, unregulated drug-to-antibody ratios (DARs), limited therapeutic activity, and a narrow therapeutic window 14, 20. Non-radioactive PSMA-targeting small-molecule drug conjugates and polymer-based nanoparticles have also become an active research area, with several conjugates and nanoparticles having entered the early stages of clinical trials 21-24. For example, A PLA-PEG-based nanoparticle platform, functionalized with small-molecule PSMA-targeting ligands, was developed as BIND-014. This therapy demonstrated efficacy and favorable tolerability in mCRPC patients who had not previously received chemotherapy (NCT01812746), however, BIND-014 failed in head and neck cancer (NCT02479178) 25-27. Therefore, it is necessary to continuously optimize the payloads, linkers, PSMA-targeting molecules, and carriers to enhance the activity and safety of the conjugate.

PSMA-targeting tracers are primarily categorized into monoclonal antibodies and small-molecule inhibitors 28. Small-molecule inhibitors are further subdivided into urea-based, phosphorus-based, and thiol-based compounds. Urea-based molecules demonstrate the broadest clinical utility owing to their superior PSMA-binding affinity relative to other classes. Among urea-based ligands, glutamate-urea-lysine (Glu-urea-Lys, EuK) and DUPA represent the most prevalent pharmacophores 29, 30. EuK confers enhanced pharmacokinetics by specifically engaging the enzymatic pocket of PSMA, outperforming radiolabeled PSMA antibodies 31. Reported PSMA-targeted radioligands predominantly utilize the EuK pharmacophore, including FDA-approved agents such as ⁶⁸Ga-PSMA-11, ¹⁸F-DCFPyL, and ¹⁷⁷Lu-PSMA-617 32. Previous studies have also demonstrated that EuK promotes the tumor-selective delivery of cytotoxic agents to PCa. A docetaxel-EuK conjugate, linked via a cleavable moiety, demonstrated strong PSMA-binding affinity, rapid internalization, and efficient intracellular drug release, yielding superior antitumor efficacy and reduced systemic toxicity compared with free docetaxel in PSMA-overexpressing xenograft models 33. Likewise, monomethyl auristatin E (MMAE) has been conjugated to EuK via a protease-cleavable linker, yielding a potent PSMA-targeted construct. This construct exhibited nanomolar activity against PSMA-positive cells, minimal toxicity toward PSMA-negative cells, and enables significant tumor regression *in vivo* 34. While these results are promising, such short peptide-drug conjugates suffer from rapid systemic clearance and very short plasma half-lives, which restrict sustained tumor exposure and impede their translation to clinical applications. These pharmacokinetic limitations, together with the potential for off-target toxicity, emphasize the need for sophisticated delivery systems like polymer-drug conjugates. Such systems would retain PSMA-targeting precision while extending circulation time, enhancing tumor accumulation, and improving the therapeutic index.

Owing to its superior biocompatibility, high aqueous solubility, and low immunogenicity, the polysaccharide polymer dextran represents an appealing platform for drug delivery. Clinically, it is utilized not only as a plasma volume expander but also as a carrier in formulations like iron dextran 35, 36. Within nanomedicine, dextran coatings enhance nanoparticle stability and prolong circulation time by minimizing protein corona formation, which in turn limits uptake by the mononuclear phagocyte system (MPS) and dampens complement activation 37-39. Such characteristics are especially advantageous for overcoming the rapid clearance and short half-life that commonly restrict peptide-drug conjugates, enabling sustained systemic exposure and enhanced therapeutic efficacy.

Polyunsaturated fatty acids (PUFAs) are capable of improving the therapeutic efficacy of chemotherapeutic agents 40, 41. In our previous study, docosahexaenoic acid (DHA), alpha-linolenic acid (ALA), and gamma-linolenic acid (GLA) were individually conjugated to CTX via a dextran backbone. Our results demonstrated that the three conjugates (Dextran-CTX-GLA, Dextran-CTX-DHA, and Dextran-CTX-ALA) all exerted potent antitumor effects, with Dextran-CTX-GLA being more effective than the other two 42. Expanding on this foundation, we incorporated the PSMA-targeting EuK ligand into dextran-CTX-GLA to generate dextran-CTX-GLA-EuK. This conjugate exhibited enhanced PSMA-specific uptake, increased tumor accumulation both i*n vitro* and *in vivo*, and potent antitumor activity in PSMA-overexpressing prostate cancer xenografts. This design thus represents a promising platform for improving chemotherapeutic selectivity and efficacy, with significant potential for clinical translation in PSMA-targeted therapy.

## Materials and Methods

### Materials

Cabazitaxel was purchased from Nanjing Dilger Medical Technology Co., Ltd. Sigma-Aldrich supplied dextran with a molecular weight of 100,000 Da. GLA was purchased from Aladdin located in Shanghai, China. All other chemical reagents were obtained from Beijing Innokai Technology Co., Ltd.

### The preparation of Dextran-CTX, Dextran-CTX-GLA, Dextran-CTX-GLA-EuK, and dextran-GLA-Cy7.5 conjugates

The full synthetic procedures for the aforementioned conjugates Dextran-CTX, Dextran-CTX-GLA, Dextran-CTX-GLA-EuK, and dextran-GLA-Cy7.5 were included in the supplementary material.

### *In vitro* release profile of CTX from Dextran-CTX-GLA-EuK in plasma and PBS

To assess the release behavior of CTX in plasma, the process began with the preparation of a standard curve. Specifically, 20 μL aliquots of CTX dissolved in methanol at concentrations of 2, 1, 0.8, 0.5, 0.25, 0.1, 0.05, and 0.02 mg/mL were each thoroughly mixed with 180 μL of Sprague-Dawley rat plasma, resulting in final concentrations of 200, 100, 80, 50, 25, 10, 5, and 2 mg/mL. Subsequently, 500 μL of a protein precipitation reagent (methanol:acetonitrile = 1:1) was introduced. After vortexing and centrifugation at 12,000 rpm for 15 min, the supernatant was filtered through a 0.22 μm filter and subjected to HPLC analysis using a C18 column. For the *in vitro* release assay, 200 μL of Dextran-CTX-GLA-EuK (10 mg/mL, CTX-equivalent, dissolved in PBS) and 200 μL of free CTX (10 mg/mL, dissolved in methanol) were individually mixed with 1.8 mL of plasma. These mixtures were incubated at 37 °C under gentle constant shaking. At predetermined time points (0.5, 1, 2, 4, 8, 12, 24, 48, and 72 h), 200 μL of the plasma sample was withdrawn, followed by the addition of 500 μL of protein precipitation solution (methanol:acetonitrile = 1:1) and mixing via shaking. After vortexing, the mixture was incubated on ice for 5 min, centrifuged at 12,000 rpm for 15 min, and the supernatant was filtered through a 0.22 µm membrane for HPLC measurement.

For assessing CTX release in PBS, a standard curve was first established. CTX dissolved in methanol at a concentration of 1 mg/mL was further diluted with a methanol:PBS (5:95) mixture to obtain serial concentrations of 150, 100, 50, 25, 10, and 5 µg/mL. These diluted solutions were subjected to HPLC analysis using a C18 column for the construction of the standard curve. For the release experiment, 1.2 mg of parent CTX or each conjugate (equivalent to 1.2 mg CTX) was dissolved in 1 mL of methanol:PBS solution (5:95) and placed into a dialysis bag (MWCO: 50,000 Da). Subsequently, the dialysis bag was placed in 39 mL of methanol:PBS solution (5:95, pH7.4) and agitated horizontally at 100 rpm at 37 °C. At predetermined time intervals (0.5, 1, 2, 4, 8, 12, 24, 48, and 72 h), 1 mL of the medium was sampled for HPLC detection, and an equal volume of fresh methanol:PBS (5:95) was supplemented. After collection, the medium was vortexed, incubated on ice for 5 min, and centrifuged at 12,000 rpm for 15 min. The supernatant was then passed through a 0.22 µm membrane prior to HPLC detection.

### Evaluation of myelosuppression induced by the conjugate in mice

BALB/c mice (male) were randomly assigned into three groups (n = 24 per group): the PBS group, the CTX group (10 mg/kg), and the Dextran-CTX-GLA-EuK group (10 mg/kg, equivalent to CTX). All agents were administered via the tail vein on day 0. On days 1, 3, 5, 7, 9, 11, and 14, 6 mice were selected from each group for blood collection to determine the counts of white blood cells (WBCs), neutrophils, red blood cells (RBCs), and platelets. The days when neutrophils reached the lowest value (neutrophil nadir) and the days required for neutrophils to recover to normal levels were recorded.

Subsequently, native BALB/c mice were obtained and randomly allocated into three groups (n = 6 per group): the PBS group, the CTX group (10 mg/kg), and the Dextran-CTX-GLA-EuK group (10 mg/kg). Blood samples were collected on day 1 after administration, at the time of neutrophil nadir, and on the day of neutrophil recovery to assess the concentrations of alanine aminotransferase (ALT), aspartate aminotransferase (AST), urea, and creatinine (CREA). Meanwhile, mice were euthanized for the counting of bone marrow nucleated cells (BMNC), flow cytometric analysis of hematopoietic stem cells, and hematoxylin and eosin (HE) staining of bone marrow tissue.

Isolation and enumeration of BMNC: Under sterile conditions, the femurs and tibias were harvested from mice. Both epiphyses, along with the epiphyseal plates, were cut off. Bone marrow was flushed from the medullary cavity into a centrifuge tube using PBS. Afterwards, red blood cell lysis buffer was added to remove red blood cells. Post centrifugation, the supernatant was aspirated. The resulting cell pellet was resuspended in 1 mL PBS, and the number of nucleated cells was quantified using trypan blue staining combined with an automated cell counter.

Histopathological analysis of bone marrow: The femurs obtained from mice was fixed in 4% formaldehyde solution. The tissue was washed with PBS and placed in a decalcification solution for continuous decalcification over 2 days. After decalcification, the femurs tissue was processed for paraffin embedding and HE staining.

Hematopoietic stem and progenitor cell staining: For staining, 1×10⁶ bone marrow cells were resuspended in FACS buffer and stained with Fixable Viability Stain 700 for 15 min at 25 °C under light protection. After 2 rounds of washing and resuspension in FACS buffer, Fc receptors were blocked utilizing anti-mouse CD16/CD32 (clone 2.4G2). Lineage-positive cells were labeled using a PerCP-Cy5.5-conjugated lineage cocktail containing antibodies against CD3e, CD11b, CD45R/B220, TER-119, and Ly6G/Ly6C (BD Biosciences). Cells were subsequently stained with antibodies against Sca-1 (Ly-6A/E)-BV421 (BD Biosciences) and c-Kit (CD117)-APC (BD Biosciences) to identify hematopoietic stem and progenitor populations. Antibody incubation was carried out in the dark at 25 °C for 30 min, after which cells were washed twice and resuspended in FACS buffer. Flow cytometry was performed on a BD FACSymphony™ A3 (BD Biosciences) flow cytometer, with data analyzed via FlowJo software. The compensation determined using single-stained controls. Debris and doublets were removed based on forward and side scatter profiles and singlet gating. After gating viable lineage-negative cells, and LSK cells were defined as Lin⁻Sca-1⁺c-Kit⁺ events. Data were analyzed using FlowJo software, and LSK frequencies were calculated relative to total bone marrow. Statistical analyses were performed using GraphPad Prism.

### Cell culture

PSMA-overexpressing prostate cancer cells (22Rv1 and LNCaP) and low-PSMA-expressing cells (PC-3 and DU145) were obtained from Pricella Biotechnology (Wuhan, China). All cells were cultivated in either DMEM or RPMI 1640 medium containing 10% fetal bovine serum, under a humidified atmosphere of 5% CO₂ at 37 °C. Lentiviral vectors carrying the PSMA overexpression plasmid were purchased from GENECHEM (Shanghai, China) and transfected into PC-3 cells. Stable cell lines PC-3/PSMA were selected by puromycin pressure (1.5 µg/mL) and subjected to subsequent experiments.

### CCK-8 cell proliferation assay

Cell growth inhibition was evaluated using a cell counting kit-8 (CCK-8) assay purchased from Beyotime Biotechnology (Shanghai, China). 22Rv1, LNCaP, PC-3, and PC-3/PSMA cells were seeded into 96-well plates at 5,000 cells/100 μL into each well and allowed to attach for 24 h. A CCK-8 assay was performed after treatment with varying concentration of CTX or Dextran-CTX-GLA-EuK for 72 h. Subsequently, 20 µL of CCK-8 reagent was dispensed into each well that contained 200 µL of culture medium. After being incubated at 37 °C for 1.5 h, the absorbance value at 450 nm was determined using a Bio-Rad 550 ELISA microplate reader. The CCK-8 assay was conducted independently in triplicate.

### Cellular uptake of Dextran-CTX-GLA-EuK

Firstly, 22Rv1 and PC-3/PSMA (PSMA-overexpressing prostate cancer cell lines) were cultured in dishes for 24 h. They were then exposed to Dextran-CTX-GLA-EuK at a final concentration of 100 ng/mL (CTX equivalent). At specified time points after incubation, cells were collected, rinsed with cold PBS, and resuspended in 1 mL PBS for counting. A portion of cells was resuspended in a 1:1 PBS-acetonitrile mixture for subsequent steps.

Within the cells, blood, and tissues, a fraction of CTX was released from the Dextran-CTX-GLA-EuK, while another portion remained conjugated. The amounts of released CTX and total CTX (comprising both the released and conjugated moieties) were subjected to separate analytical detection.

To quantify released CTX, 450 μL of the cell suspension was combined with 50 μL of internal standard (2 μg/mL DTX in acetonitrile). Subsequent steps included sonication on ice for 2 min and centrifugation at 12,000 rpm for 15 min at 4 °C. After filtration through a 0.22 µm membrane filter, the clarified supernatant was analyzed via HPLC/MS.

For the determination of total CTX, a 450 μL aliquot of cell suspension was blended with 50 μL Boc-linker-Phenylalanine internal standard solution (prepared at 2 μg/mL in acetonitrile), subjected to ice-cold sonication for 2 min, and centrifuged at a suitable speed. A 380 μL aliquot of the supernatant was combined with 20 μL of 1 g/mL aqueous sodium hydroxide solution, incubated at 28 °C with shaking at 200 rpm for 6 h, adjusted to pH 6.0 with glacial acetic acid, and centrifuged again. Following filtration through a 0.22 μm membrane filter, the resulting supernatants were subjected to HPLC/MS analysis to determine total CTX content.

### Cellular uptake and *in vitro* cell binding assay

Prostate cancer cells were plated in 6-well dishes and incubated for 24 h, then exposed to Dextran-GLA-Cy7.5 for various durations. Subsequently, the intracellular fluorescence intensity was quantified using flow cytometry. For the cell binding assay, the Dextran-GLA-Cy7.5 solution was supplemented with 2-(phosphonomethyl) pentanedioic acid (2-PMPA, a widely used PSMA inhibitor) at a final concentration of 1.0 mM. After 2 h of incubation with the cells, the cells were rinsed and their fluorescence intensity was measured. For the analysis of endocytic pathways, 22Rv1 and PC-3/PSMA cells were first cultured in 6-well plates for 24 h, then incubated in serum-free medium for 2 h. Subsequently, the cells were treated with a mixture of endocytic inhibitors and Dextran-GLA-Cy7.5 for another 2 h, and the intracellular fluorescence intensity was measured via flow cytometry. The concentrations of the applied inhibitors were as follows: 100 μM of the macropinocytosis inhibitor EIPA, 1 μg/mL of the clathrin-dependent endocytosis inhibitor chlorpromazine, and 1 μM of the caveolin-dependent endocytosis inhibitor methyl-β-cyclodextrin.

### *In vivo* and *ex vivo* imaging for PSMA-mediated tumor targeting evaluation

PSMA-overexpressing 22Rv1 xenograft model was established to assess the tumor-targeting ability of Dextran-CTX-GLA-EuK. After the tumor volume was measured to be approximately 100 mm³, the mice received intravenous injections of Cy7.5, Dextran-Cy7.5 and Dextran-Cy7.5-EuK via the tail vein, each at a dose of 1.5 mg/kg (corresponding to Cy7.5). As for the blocking study, 2-PMPA (10 mg/kg) dissolved in saline was injected intravenously into the mice. At 15 min after the injection of 2-PMPA, Cy7.5, Dextran -Cy7.5 and Dextran-Cy7.5-EuK were injected intravenously into the mice. At various time points post-administration, the mice were anesthetized and imaged using the Xenogen IVIS Lumina system (Caliper Life Sciences, USA) to capture fluorescent signals. For *ex vivo* imaging, the mice were euthanized at 96 h after injection, and their primary organs were harvested and imaged. All acquired imaging data were processed and analyzed with Living Image 4.1 software.

### Determination of released CTX and total CTX in plasma and tissues

For the quantification of released CTX, samples were mixed with pH 7.4 PBS and homogenized on ice. A DTX internal standard (dissolved in 1:1 methanol:acetonitrile) was added, followed by centrifugation at 12,000 rpm for 15 min. After filtering through a 0.22 µm membrane, the supernatants were analyzed via HPLC/MS.

For the quantification of total CTX, tissue or blood specimens were mixed with pH7.4 PBS and homogenized under ice-cold conditions. An internal standard consisting of Boc-linker-Phenylalanine dissolved in a 1:1 (v/v) mixture of methanol and acetonitrile was introduced, followed by centrifugation of the resulting mixture at 12,000 rpm for 15 min. Subsequently, sodium hydroxide was added to the collected supernatants to reach a final concentration of 1 g/mL. After shaking at 200 rpm for 6 h at 25 °C and adjusting the pH to 6.0 using glacial acetic acid, the samples were centrifuged once more. The obtained supernatants were filtered through a 0.22 μm membrane filter prior to HPLC/MS analysis.

### Tumor inhibition studies in 22Rv1 and PC-3/PSMA xenograft models

All animal experiments performed in this study were reviewed and approved by the Animal Care and Welfare Committee of the School of Life Sciences, Shandong University (Approval No.: SYDWLL-2023-042). Six-week-old male BALB/c (nu/nu) nude mice (20 ± 2 g), purchased from Beijing Vital River Laboratory Animal Technology Co., Ltd., were utilized in the experiments. Each mouse was subcutaneously implanted with 6 million cells of either 22Rv1 or PC-3/PSMA into the right flank. Once the average tumor volume attained 100-200 mm³, the mice bearing tumors were randomly allocated to nine groups, consisting of 6 mice per group. The control group received only PBS administration. For the treatment groups, parent CTX and its conjugates (at doses of 5 mg/kg and 10 mg/kg, equivalent to parent CTX) were administered via intravenous injection once a week. Tumor diameters and mouse body weights were monitored twice per week. Tumor volume was measured and calculated via the formula: V = (a × b²)/2, where V denotes volume, a refers to the major axis length, and b stands for the short axis length of the tumor. For the 22Rv1 model, a survival study was conducted. For the PC-3/PSMA model, nude mice were euthanized on the 21 days. Blood samples were obtained for the examination of liver function, renal function, and blood routine parameters, while normal tissues and tumor were harvested for HE staining.

### Statistical analysis

All data are presented as the Mean ± Standard Deviation (SD), with data derived from a minimum of three independent *in vitro* experiments and six tumor samples per group for *in vivo* studies. Comparisons between two groups were conducted via Student’s test, whereas differences across three or more groups were assessed by one-way analysis of variance (ANOVA). A P value of less than 0.05 was regarded as statistically significant.

## Results and Discussion

### Chemical synthesis and characterization of the conjugates

**Scheme 1:**
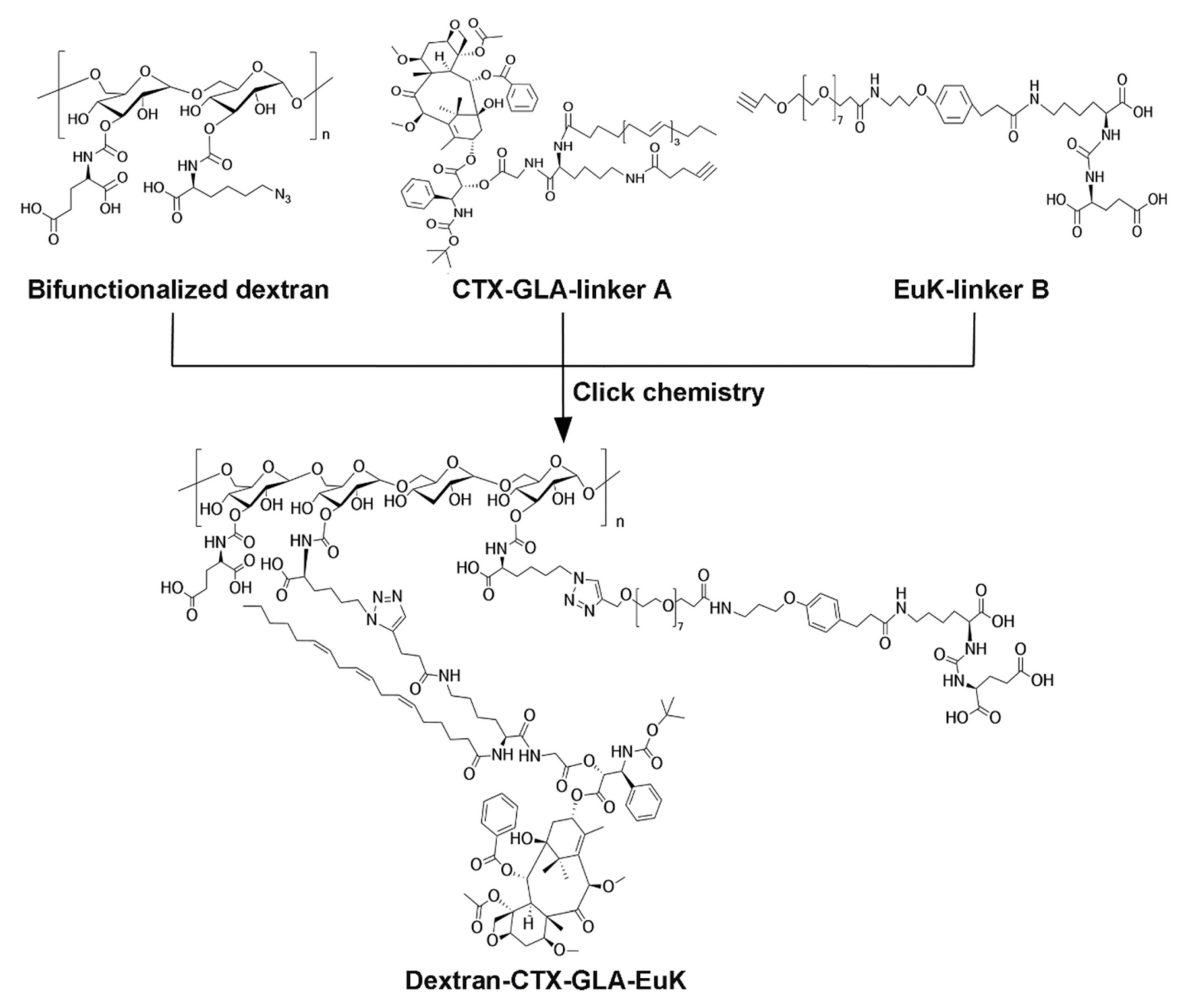
The synthetic route of the conjugate Dextran-CTX-GLA-EuK. EuK denotes the PSMA-targeting small-molecule moiety Glu-urea-Lys.

Firstly, a bifunctional dextran modified with both azide groups and glutamic acid was first synthesized using the protocol described in our prior study 42 (**Scheme S1**). Secondly, compound CTX-GLA-linker A was prepared using the method from the same work (**Scheme S2**). Thirdly, the EuK-linker B was synthesized as described in supplementary materials (**Scheme S3**). To enhance the affinity of the EuK moiety within the conjugate for PSMA-overexpressing cancer cells, we selected (PEG)_8_ to tether EuK to dextran. Finally, CTX-GLA-linker A, and EuK-linker B were grafted to bifunctionalized dextran to obtain the conjugate Dextran-CTX-GLA-EuK through click chemistry reaction (**Scheme 1**). This conjugation allows modulation of CTX and EuK pharmacophore ratios by adjusting the molar ratios among these three components, while exhibiting high yield, excellent selectivity, rapid reaction kinetics, and industrial scalability. In this study, two conjugate groups—Dextran-CTX and Dextran-CTX-GLA—were synthesized independently in this study (**Figure S1**).

**Figure 1:**
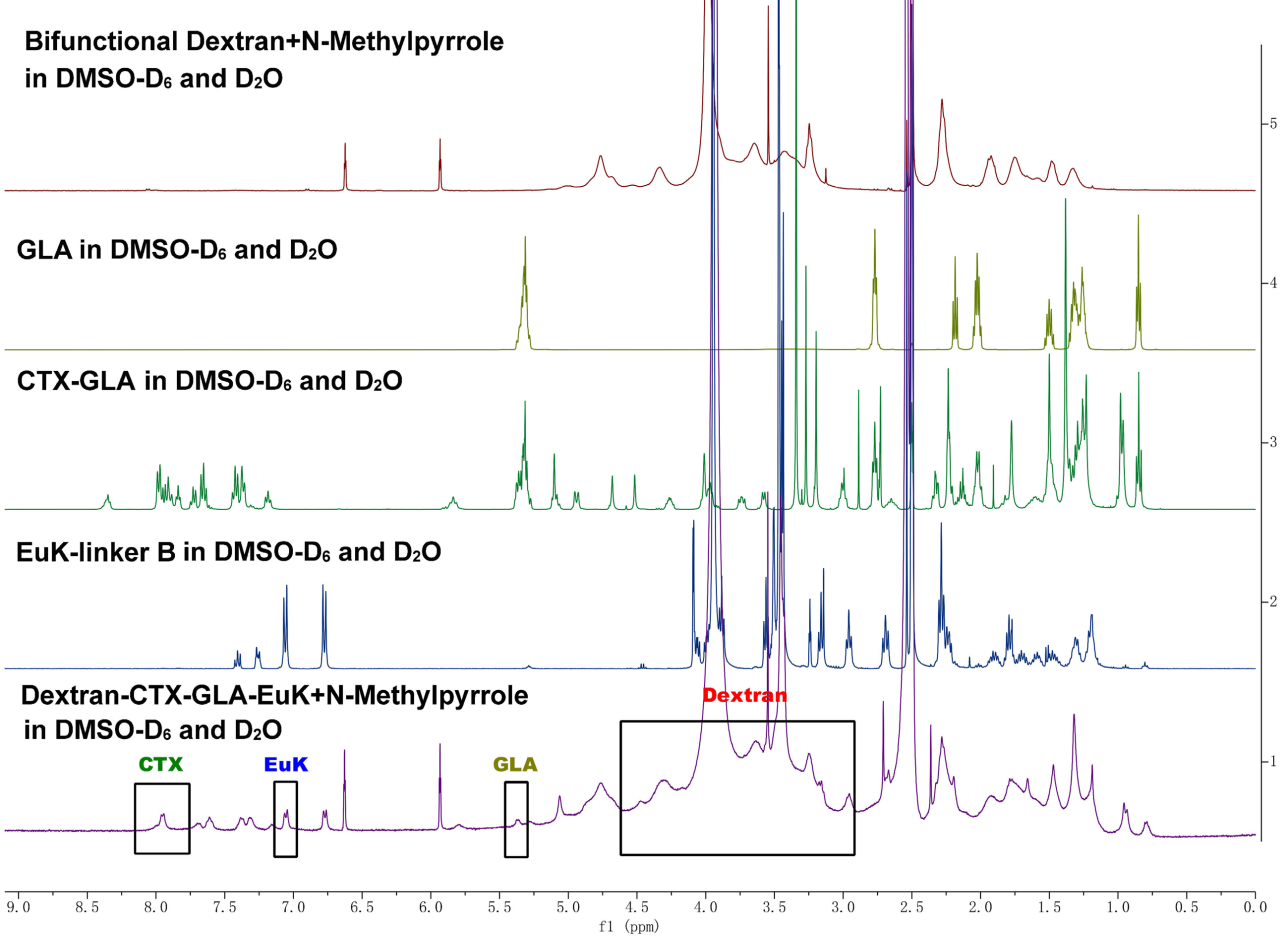
^1^H NMR spectra of dextran, GLA, CTX-GLA, EuK-linker B and Dextran-CTX-GLA-EuK.

**Figure 2:**
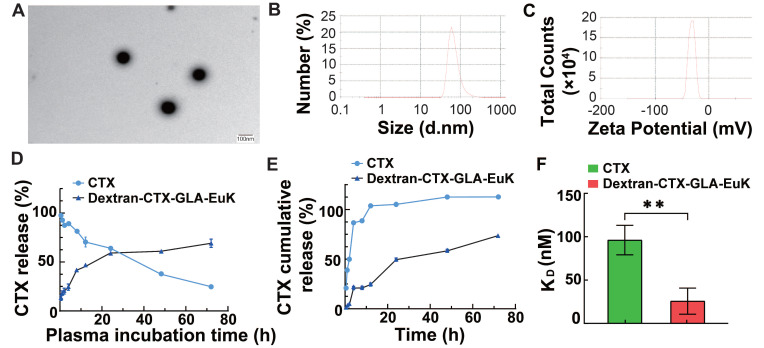
Characterization of the conjugate Dextran-CTX-GLA-EuK. (A) TEM image at 1 mg/mL. (B) Particle size distribution. (C) Surface charge. (D) CTX release from the conjugate in rat plasma. (E) CTX release from the conjugate in PBS solution. (F) The affinity of the conjugate for PSMA measured by SPR. Data were expressed as mean ± SD (n = 3).

The structure of Dextran-CTX-GLA-EuK was confirmed by comparing the ¹H NMR spectra of dextran, GLA, CTX-GLA, and EuK-linker B (**Figure 1**). Gel permeation chromatography (GPC) analysis showed that the purity of Dextran-CTX-GLA-EuK exceeded 98%. The CTX content in dextran-based conjugates was determined via ¹H NMR using an internal standard (N-methyl pyrrole, chemical shift: 6.6 ppm). The target signal was the β-site protons (two H atoms) of one benzene ring in CTX (chemical shift: ~8.00 ppm). The CTX contents (w/w) of Dextran-CTX, Dextran-CTX-GLA, and Dextran-CTX-GLA-EuK were 12.4%, 11.5%, and 11.8%, respectively. Since the molar ratio of CTX to GLA was 1:1 in both Dextran-CTX-GLA and Dextran-CTX-GLA-EuK, their GLA contents (w/w) were calculated as 3.8% and 3.9%, respectively. EuK-containing PSMA-targeting moiety exhibited two distinct benzene ring proton signals at 6.86 ppm and 7.13 ppm, respectively. The absence of interfering signals from neighboring peaks makes these signals suitable for quantitative analysis of the PSMA-targeting moiety. The content of EuK in Dextran-CTX-GLA-EuK is 5.3%. The conjugate Dextran-CTX-GLA-EuK was readily soluble in water, forming a pale-yellow solution. The aqueous solubility of Dextran-CTX-GLA-EuK was 23.6 ± 1.5 mg/mL (equivalent to CTX), while parent CTX is poorly soluble in water. Transmission electron microscopy (TEM) revealed that Dextran-CTX-GLA-EuK at 1.0 mg/mL formed spherical nanoparticles in aqueous solution, with a mean diameter of 95.62 ± 5.45 nm (**Figure 2A-B**). The zeta potential of Dextran-CTX-GLA-EuK was -31.80 ± 2.25 mV (**Figure 2C**). The above characteristics indicated that the Dextran-CTX-GLA-EuK was suitable as a drug delivery system.

The release behaviors of CTX from the Dextran-CTX-GLA-EuK conjugate were investigated in both plasma and PBS. For plasma assays, 200 µL conjugate solution (containing 0.40 mg CTX) was combined with 1800 µL rat plasma, followed by incubation at 37 °C with agitation. Aliquots collected at designated intervals were subjected to quantification of free CTX. Results showed that parent CTX in plasma decreased in time-dependent manner; however, Dextran-CTX-GLA-EuK slowly released CTX and maintained a release rate of around 65.1% (**Figure 2D**). Parallel evaluation in PBS employed dialysis methodology. 1 mL conjugate (containing 2.0 mg CTX) was loaded into a dialysis bag immersed in 39 mL PBS (pH7.4) at 37 °C under mild agitation. Following sampling of 1 mL PBS at predetermined time points, HPLC-based measurements indicated Dextran-CTX-GLA-EuK slowly released CTX in PBS, reaching its peak at 24 h and maintaining a release rate of 69.2%. As a control, almost all parent CTX can be fully detected at 9 h. These results demonstrated that Dextran-CTX-GLA-EuK exhibited greater stability than parent CTX and Dextran-CTX-GLA-EuK can slowly release CTX under normal physiological conditions.

The surface plasmon resonance (SPR) technique allows for the direct quantification of interactions between a protein and its binding ligand. The binding affinity of Dextran-CTX-GLA-EuK for recombinant human PSMA protein was determined by SPR. Results indicated that the dissociation equilibrium constant (KD) of Dextran-CTX-GLA-EuK was substantially lower than that of Dextran-CTX-GLA, indicating stronger binding specificity of Dextran-CTX-GLA-EuK toward PSMA (**Figure 2F**).

### Myelosuppression induced by Dextran-CTX-GLA-EuK in mice

**Figure 3:**
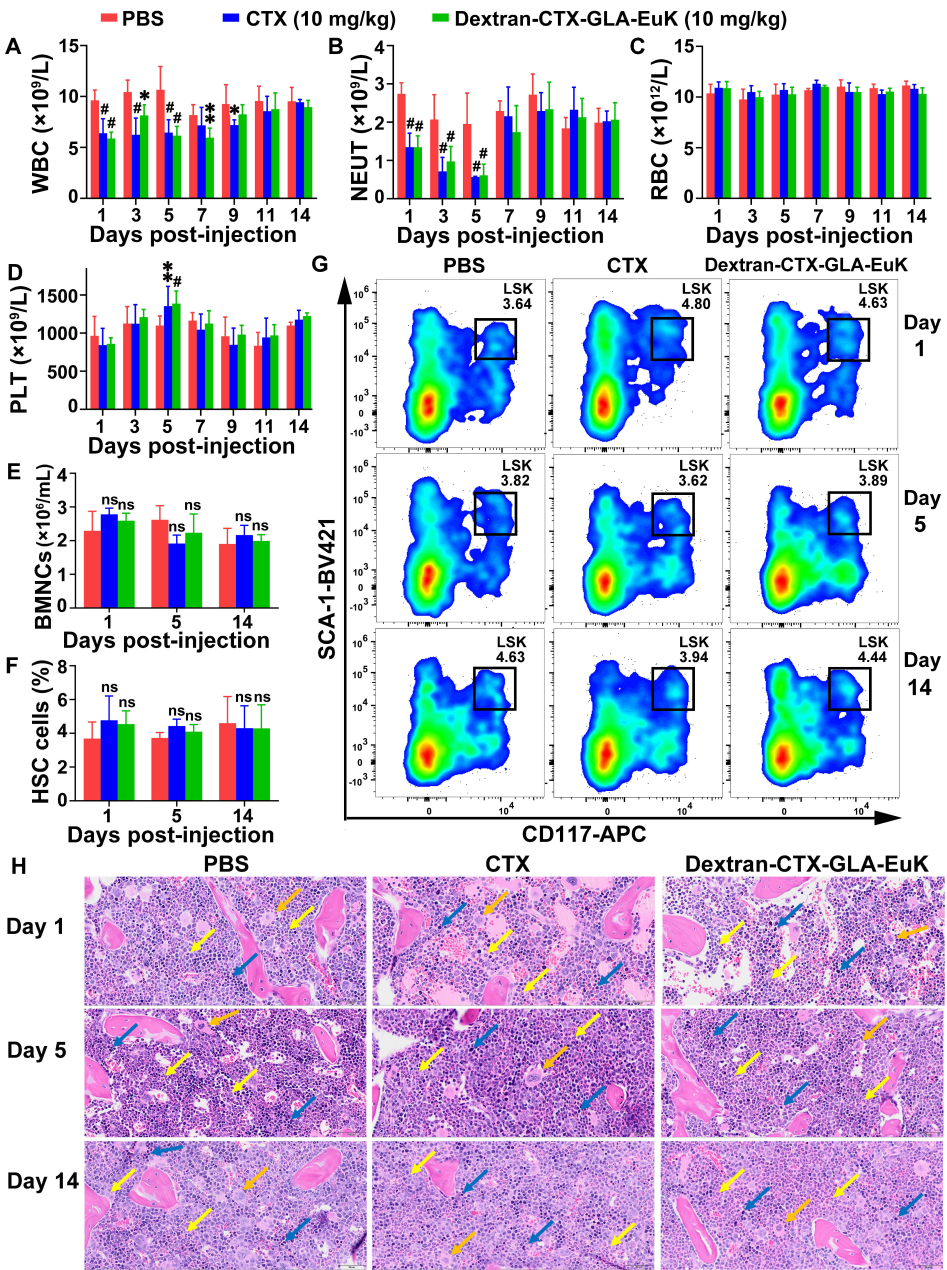
** Myelosuppression induced by the conjugate Dextran-CTX-GLA-EuK in mice.** (A-D) Effects of the conjugate (10 mg/kg) on peripheral blood parameters, including WBC, NEUT, RBC and PLT. (E) Impact of the conjugate on bone marrow nucleated cell (BMNC) counts. (F) Effect of the conjugate on hematopoietic stem cell (HSC) populations in the bone marrow. (G) Quantification of bone marrow LSK cells by flow cytometry. (H) Histological analysis of femur bone marrow sections. Granulocytes (yellow arrow), lymphocytes (blue arrow) and megakaryocytes (orange arrow) were indicated (original magnification × 200). *p < 0.05, **p < 0.01, ***p < 0.001, ^#^p < 0.0001, ns means p > 0.05, vs PBS group in the same day (n ≥ 3).

It is well known that CTX mainly induces myelosuppression, including symptoms such as neutropenia, anemia, and thrombocytopenia. After intravenous tail vein injection of parent CTX and the conjugate Dextran-CTX-GLA-EuK (10 mg/kg, equivalent to CTX) into BALB/c mice, no changes were observed in red blood cell (RBC) count, platelet count, hemoglobin (HGB), or hematocrit (HCT) within 14 days. However, the white blood cell (WBC) and neutrophil (NEUT) counts decreased sharply on day 1, reached the nadir on day 5, and then recovered to normal levels on day 7. Notably, the WBC and NEUT levels on day 5 were still within the normal range for healthy BALB/c mice 43, 44. Consistent with previous literature reports, the time-dependent changes in NEUT counts induced by CTX in this study align with established findings on CTX-related myelosuppression 45-47. No significant alterations were noted in comparison with the control group in AST, ALT, UREA, or CREA levels within 14 days (**Figure 3 A-D, Figure S2**). Additionally, administration of Dextran-CTX-GLA-EuK did not cause changes in the number of nucleated cells (BMNCs) or hematopoietic stem cells (HSCs) in the femurs and tibias (**Figure 3 E-G**). HE staining of femoral bone marrow revealed no histological differences among the PBS, CTX, and Dextran-CTX-GLA-EuK treatment groups (**Figure 3H**). All groups presented abundant hematopoietic cells in the bone marrow cavity, with hematopoietic cells at various developmental stages identified. A large number of granulocytes and lymphocytes, along with a small number of megakaryocytes, were observed with densely arranged cellular morphology in any group. Additionally, a rich distribution of vascular sinuses was detected in the bone marrow cavity, and no cellular damage or adipocyte hyperplasia was noted across all groups. Combined with the findings that the numbers of BMNCs and HSCs remained unaltered, these results fully confirmed that Dextran-CTX-GLA-EuK at this administered dose only induced a transient mild reduction in peripheral blood WBC and NEUT counts, without impairing the hematopoietic microenvironment or bone marrow hematopoietic stem/progenitor cells. These findings suggested that the PSMA-targeted conjugate exhibited favorable safety regarding myelotoxicity at 10 mg/kg.

### Dextran-CTX-GLA-EuK inhibited cell growth

To investigate the antitumor effects of Dextran-CTX-GLA-EuK against PCa cells with varying PSMA expression, this study utilized PSMA-overexpressing cells (22Rv1, LNCaP, PC-3/PSMA) and low-PSMA-expressing cells (PC-3 and DU145). The PSMA expression levels of these cells are shown in **Figure S3**. Among them, PC-3/PSMA cells are a stable transfected cell line derived from PC-3 cells infected with lentivirus expressing PSMA. CCK-8 assays revealed that Dextran-CTX-GLA-EuK exhibited marginally higher cytotoxic effects than the parent CTX and other conjugates, although these differences failed to reach statistical significance after 72 h of incubation with PSMA-overexpressing cells (**Table 1**). The IC_50_ value of Dextran-CTX-GLA-EuK in PC-3/PSMA cells was significantly lower than that in PC-3 cells, indicating that Dextran-CTX-GLA-EuK exhibited stronger cytotoxicity in PSMA-overexpressing cells.

### Cellular uptake and *in vitro* cell binding assay

**Figure 4:**
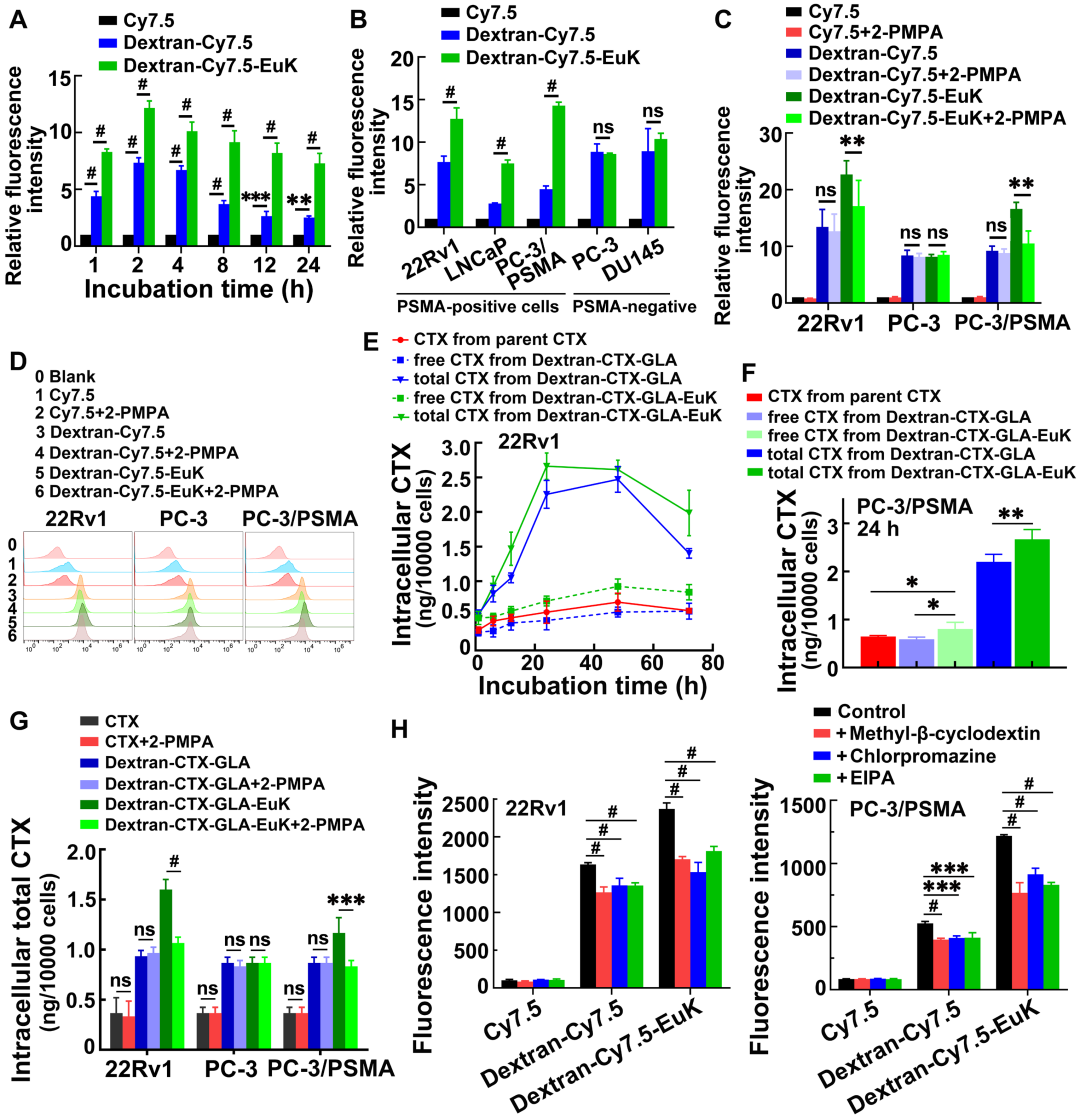
Cellular uptake and endocytic pathways of the conjugate Dextran-CTX-GLA-EuK (n = 3). (A) The fluorescence intensity in 22Rv1 cells after incubation at indicated time. (B) The fluorescence intensity of the cellular uptake of fluorescent conjugate Dextran-Cy7.5 and Dextran-Cy7.5-Euk in different cells incubated for 2 h. (C, D) *In vitro* cellular binding of Dex-Cy7.5-EuK with 1 mM 2-PMPA for 2 h. (E) Intracellular levels of free CTX and total CTX in 22Rv1 cells incubated with CTX, Dextran-CTX-GLA and Dextran-CTX-GLA-EuK for varying time. (F) Free CTX and total CTX contents in PC-3/PSMA cells incubated with Dextran-CTX-GLA-EuK for 24 h. (G) *In vitro* cellular binding of Dextran-CTX-GLA-EuK with 1 mM 2-PMPA for 2 h. (H) The effect of endocytic pathway inhibitors on the cellular uptake of Dextran-Cy7.5-Euk in 22Rv1 and PC-3/PSMA cells. Data are mean ± SD; *p < 0.05, **p < 0.01, ***p < 0.001, #p < 0.0001, ns means p > 0.05.

To evaluate the PSMA-targeting ability of Dextran-CTX-GLA-EuK, we synthesized two fluorescent derivatives, Dextran-Cy7.5 and Dextran-Cy7.5-EuK. In 22Rv1 cells overexpressing PSMA, Dextran-Cy7.5-EuK exhibited significantly higher fluorescence intensity compared to Dextran-Cy7.5 and free Cy7.5 over a 24-hour period. (**Figure 4A**). Consistently, Dextran-Cy7.5-EuK displayed significantly stronger fluorescence than Dextran-Cy7.5 in PSMA-overexpressing cell lines (22Rv1, LNCaP, PC-3/PSMA), but not in PSMA-low cell lines (PC-3, DU145) (**Figure 4B**). The uptake of Dextran-Cy7.5-EuK in PC-3/PSMA cells was 1.7-fold higher than that in PC-3 cells, while no such difference was observed for Dextran-Cy7.5. Both conjugates, Dextran-Cy7.5-EuK and Dextran-Cy7.5, showed markedly higher intracellular fluorescence than free Cy7.5 in all five prostate cancer cell lines, verifying the improved internalization of dextran-based systems.

To confirm PSMA-mediated endocytosis, 2-PMPA, a specific PSMA inhibitor, was used. Following co-incubation with 1 mM 2-PMPA for 2 h, the fluorescence intensity of Dextran-Cy7.5-EuK was significantly reduced in 22Rv1 and PC-3/PSMA cells, but remained unchanged in PC-3 cells (**Figure 4C-D**). In contrast, 2-PMPA exerted no effect on the fluorescence signals of Dextran-Cy7.5 or Cy7.5 in these cell lines.

Intracellular CTX levels were further quantified. In 22Rv1 and PC-3/PSMA cells, the total CTX content from Dextran-CTX-GLA-EuK and Dextran-CTX-GLA was markedly higher than the released CTX, indicating sustained intracellular CTX release (**Figure 4E-F**). Dextran-CTX-GLA-EuK exhibited markedly higher levels of both free and total CTX than Dextran-CTX-GLA and parent CTX, demonstrating enhanced cellular internalization. Co-incubation with 2-PMPA significantly decreased the intracellular content of Dextran-CTX-GLA-EuK in 22Rv1 and PC-3/PSMA cells, but not in PC-3 cells (**Figure 4G**). However, 2-PMPA did not affect the levels of CTX or Dextran-CTX-GLA in PSMA-positive cells, nor any of the tested agents in PC-3 cells. Collectively, these findings suggest that Dextran-CTX-GLA-EuK enhanced intracellular accumulation through specific binding to the PSMA receptor.

Finally, to elucidate the cellular internalization pathway of Dextran-CTX-GLA-EuK, cells were incubated with Dextran-Cy7.5-EuK in the presence of 100 μM EIPA (a macropinocytosis inhibitor), 1 μg/mL chlorpromazine (a clathrin-dependent endocytosis inhibitor), and 1 μM methyl-β-cyclodextrin (a caveolin-dependent endocytosis inhibitor), respectively (**Figure 4H**). The findings demonstrated that all three inhibitors observably reduced the intracellular fluorescence intensity of Dextran-Cy7.5-EuK, indicating that the dextran-based conjugates were internalized through multiple endocytic pathways.

### Dextran-CTX-GLA-EuK targeted PSMA in prostate cancer* in vivo*

**Figure 5:**
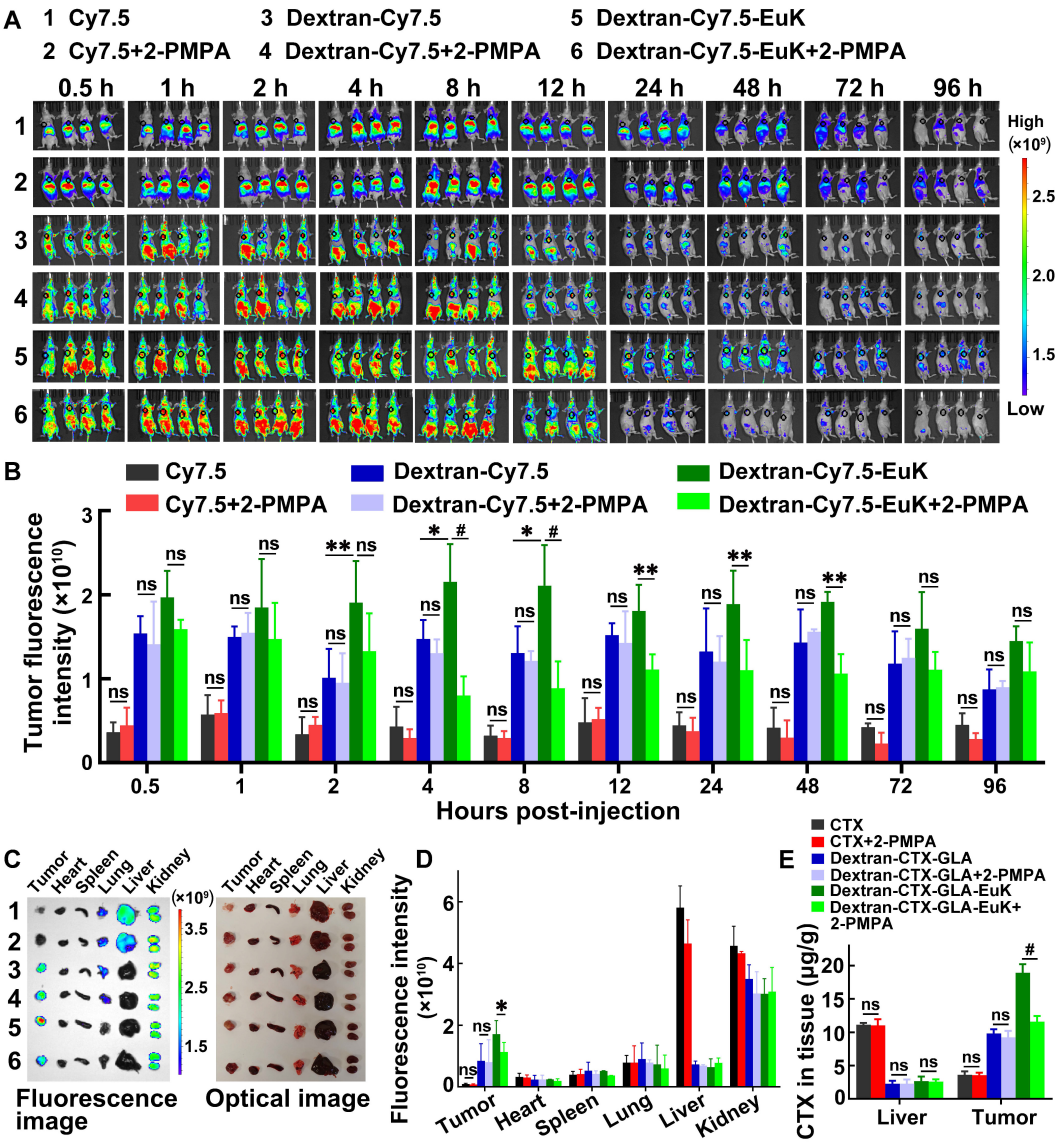
Dextran-Cy7.5-EuK and Dextran-CTX-GLA-EuK specifically accumulated in tumors of PSMA-overexpressing 22Rv1 xenograft model. (A) *In vivo* fluorescence imaging was performed on nude mice after intravenous injection of Cy7.5, Dextran-Cy7.5, and Dextran-Cy7.5-EuK, with or without 15-min pretreatment with 10 mg/kg 2-PMPA. The tumor foci were delineated with black circles (n = 4). (B) Quantification of tumor fluorescence intensity in (A). (C) *Ex vivo* fluorescence and optical images of major tissues at 96 h post-injection of Dextran-Cy7.5-EuK, with or without 2-PMPA (n = 3). (D) Quantification of *ex vivo* fluorescence intensity in different tissue samples from Figure C. (E) At 8 h after Dextran-CTX-GLA-EuK injection (with or without 2-PMPA), total CTX content was quantified in tissues of tumor-bearing mice (n = 4). *p < 0.05, **p < 0.01, #p < 0.0001, ns means p > 0.05.

To assess the tumor-targeting efficacy of EuK-modified conjugates, Cy7.5, Dextran-Cy7.5, and Dextran-Cy7.5-EuK were intravenously injected into nude mice via the tail vein. These mice were xenografted with PSMA-overexpressing 22Rv1 tumors. From 1 to 96 h post-injection, the fluorescence intensities of Dextran-Cy7.5 and Dextran-Cy7.5-EuK in tumors were significantly higher than that of free Cy7.5, indicating that dextran-based conjugates accumulated more efficiently in tumor tissues than small molecules (**Figure 5A–B**). The fluorescence intensity of Dextran-Cy7.5-EuK in tumors was also markedly higher than that of Dextran-Cy7.5 within 96 h. At 96 h post-administration, major tissues were collected and their fluorescence intensities were quantified. Dextran-Cy7.5-EuK showed substantially higher fluorescence intensity in tumors than both Dextran-Cy7.5 and free Cy7.5, accompanied by lower signals in most normal tissues. All three agents (Dextran-Cy7.5-EuK, Dextran-Cy7.5, and Cy7.5) accumulated in the kidneys, which was attributed to the renal excretion of Cy7.5 (**Figure 5C-D**), as the degraded low-molecular-weight conjugates (~50 kDa) could be efficiently cleared by the kidneys.

For the blocking study, 2-PMPA (10 mg/kg) was intravenously injected into mice. After 15 min of 2-PMPA pretreatment, Dextran-Cy7.5-EuK was administered via the tail vein, and tumor fluorescence intensity was monitored at various time points within 96 h. These findings indicated that 2-PMPA markedly decreased the tumor accumulation of Dextran-Cy7.5-EuK within 96 h, but did not affect the fluorescence intensities of Cy7.5 or Dextran-Cy7.5 in tumors (**Figure 5A-D**). 2-PMPA also did not alter the fluorescence signals of the conjugates in other tissues. Furthermore, tumors and livers were harvested at 8 h after intravenous administration of 2-PMPA and Dextran-CTX-GLA-EuK, and the total CTX content in these tissues was determined. 2-PMPA markedly decreased the tumor content of Dextran-CTX-GLA-EuK to a level comparable to that of Dextran-CTX-GLA, but did not change its content in the liver (**Figure 5E**). Accordingly, 2-PMPA did not affect the tumor levels of parent CTX or Dextran-CTX-GLA. Notably, the intratumoral total CTX level in the Dextran-CTX-GLA-EuK group was markedly higher than that in the parent CTX group, whereas its hepatic content was markedly lower than that of parent CTX. Collectively, these findings demonstrated that Dextran-CTX-GLA-EuK enabled highly specific and active targeting to PSMA-overexpressing tumor.

**Figure 6:**
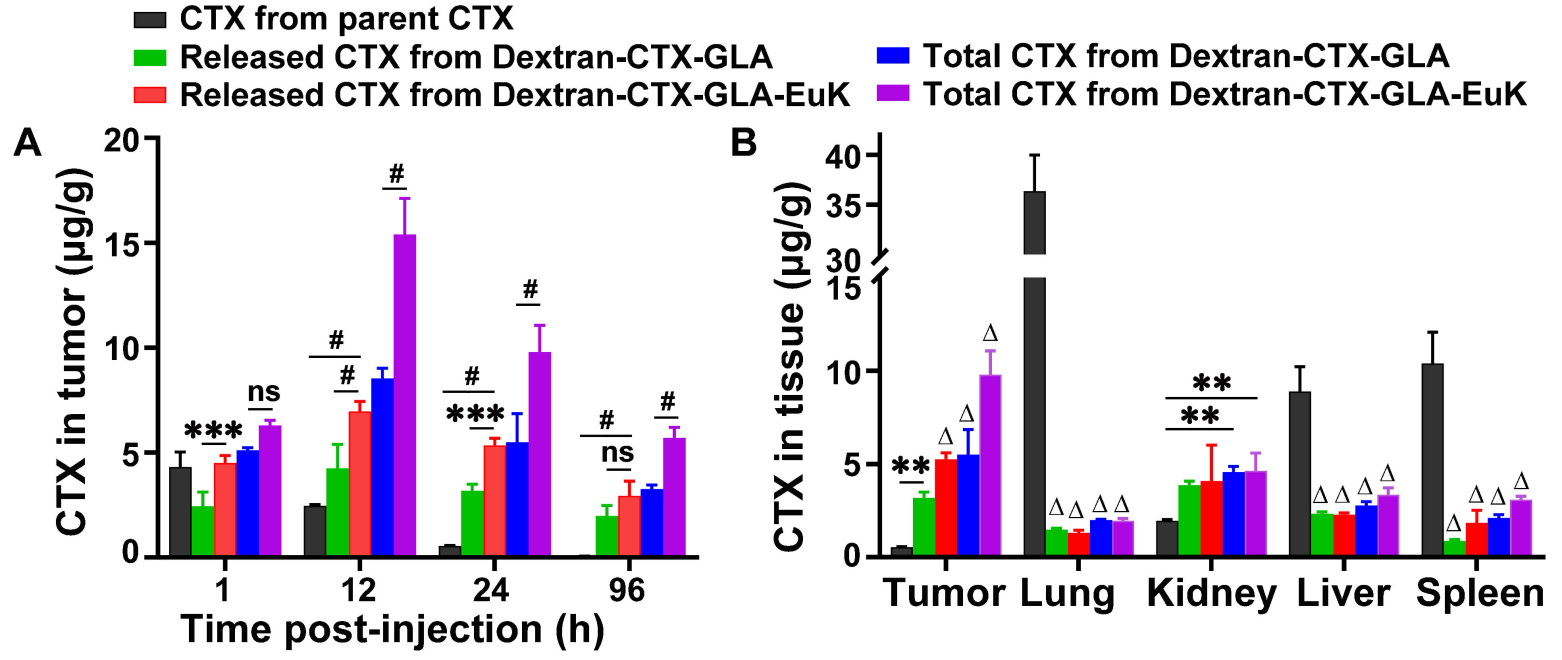
Biodistribution of Dextran-CTX-GLA-EuK in PSMA-overexpressing 22Rv1 xenograft model. (A) Intratumoral levels of free CTX and total CTX in mice administered with parenteral CTX, Dextran-CTX-GLA and Dextran-CTX-GLA-EuK. (B) Free CTX and total CTX content in tumor, lung, kidney, liver and spleen at 24 h post Dextran-CTX-GLA-EuK injection. **p < 0.01, ***p < 0.01, #p < 0.0001, ns means p > 0.05. Δ p < 0.0001, vs the CTX group in the same tissue.

Subsequently, the tissue distribution profile of Dextran-CTX-GLA-EuK was examined in 22Rv1 tumor-bearing nude mice. The intratumoral level of parent CTX declined gradually from 4.306 μg/g at 1 h to 0.058 μg/g at 96 h (**Figure 6A**). Conversely, the intratumoral levels of Dextran-CTX-GLA and Dextran-CTX-GLA-EuK peaked at 12 h, then decreased, but still remained at a relatively high level at 96 h. At 1, 12, 24, and 96 h, the free CTX contents in tumors of the Dextran-CTX-GLA-EuK group were 1.04, 2.84, 9.76, and 50.50 times those of the parent CTX group, respectively. Similarly, the total CTX contents in tumors of the Dextran-CTX-GLA-EuK group were 1.46, 6.27, 17.90, and 98.31 times those of the parent CTX group, respectively. Furthermore, at different time points assessed, the free CTX and total CTX levels in the tumors of the Dextran-CTX-GLA-EuK group were also markedly enhanced in comparison to those in the Dextran-CTX-GLA group. At 24 h after injection, the CTX contents in the tumors of the parent CTX group were much lower than their levels in normal tissues (liver, spleen, and lung). Conversely, the contents of free CTX and total CTX in the tumors of Dextran-CTX-GLA-EuK group were significantly higher than their levels in normal tissues (**Figure 6B**). Additionally, the levels of free CTX and total CTX in normal tissues of Dextran-CTX-GLA-EuK group were markedly lower than those in the parent CTX group. These findings demonstrated that Dextran-CTX-GLA-EuK had a targeted enrichment effect in PSMA-highly expressed tumors, while exhibiting minimal distribution in normal tissues.

### *In vivo* antitumor efficacy of Dextran-CTX-GLA-EuK on 22Rv1 and PC-3/PSMA xenograft tumors

The *in vivo* anti-tumor effect of Dextran-CTX-GLA-EuK was evaluated in nude mice bearing PSMA-overexpressing PCa cells 22Rv1 and PC-3/PSMA xenografts. The parent drug CTX, Dextran-CTX, Dextran-CTX-GLA, and Dextran-CTX-GLA-EuK were administered via tail vein injection at 5 and 10 mg/kg, respectively, once a week. The 22Rv1 xenografts were administered four times, while the PC-3/PSMA xenografts were administered three times.

**Figure 7:**
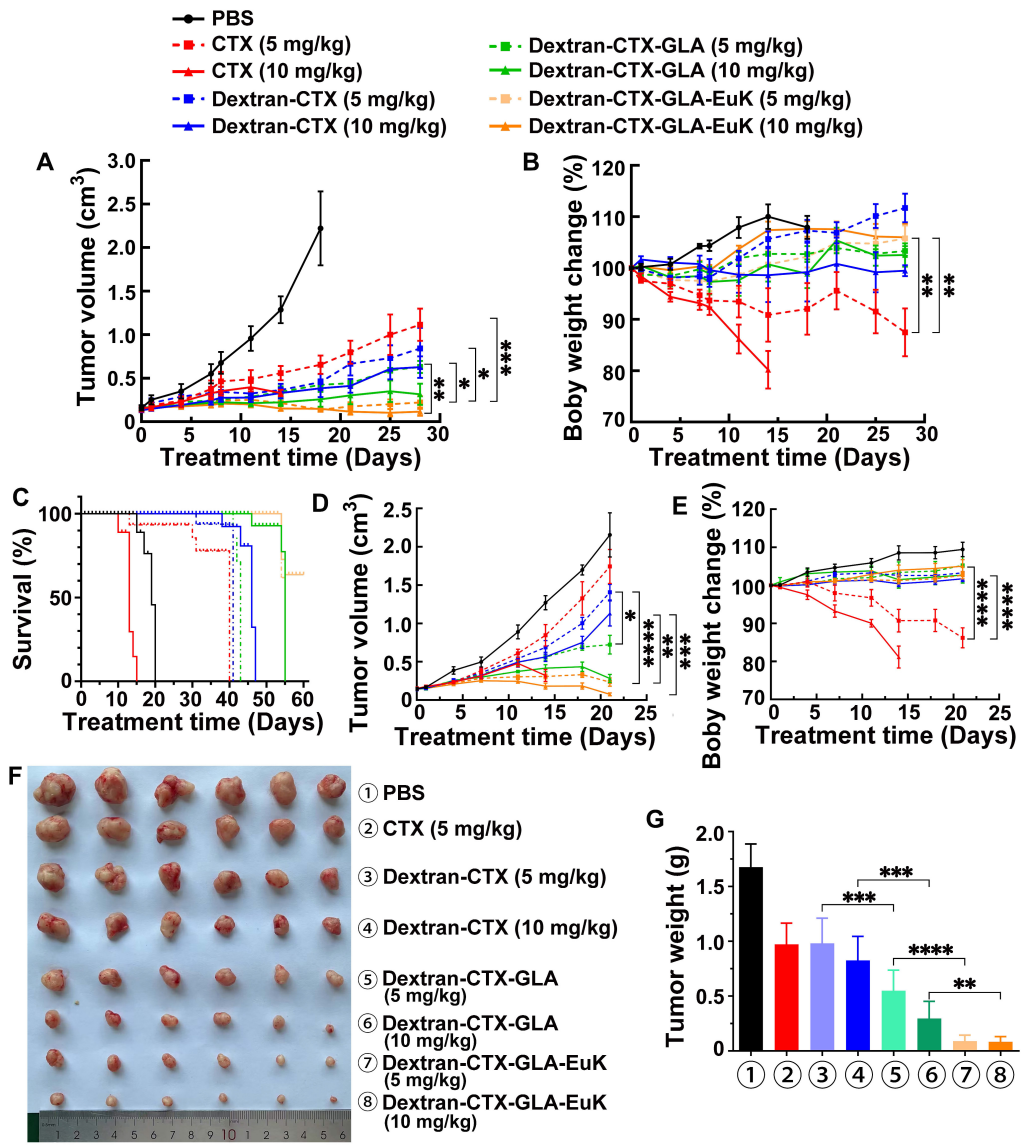
Dextran-CTX-GLA-EuK inhibited PCa growth of 22Rv1 and PC-3/PSMA xenograft models. (A-C) Analysis of tumor volume, body weight, and survival rates in the 22Rv1 model. (D-G) Tumor volume, body weight, tumor photograph and tumor weight in PC-3/PSMA model. Nude mice harboring xenografts were treated with weekly intravenous injections of CTX or different conjugates for 4 or 3 weeks, respectively. Data are presented as the mean ± SD, *p < 0.05, **p < 0.01, ***p < 0.001 and ****p < 0.0001.

In both 22Rv1 and PC-3/PSMA xenografts, two dose levels of parent CTX and each conjugate suppressed tumor growth in a dose-dependent fashion, and all conjugates demonstrated enhanced antitumor efficacy relative to equivalent doses of the parent drug CTX. In both xenograft models, at equivalent doses (5 mg/kg or 10 mg/kg), the antitumor activity increased in the following order: CTX < Dextran-CTX < Dextran-CTX-GLA < Dextran-CTX-GLA-EuK (**Figure 7**). In the 22Rv1 xenograft model, the survival duration of nude mice treated with Dextran-CTX, Dextran-CTX-GLA, and Dextran-CTX-GLA-EuK progressively extended (**Figure 7C**). The CTX group showed the shortest survival, while the Dextran-CTX-GLA-EuK group exhibited the longest survival. In the PC-3/PSMA xenograft model, Dextran-CTX-GLA-EuK at 5 mg/kg and 10 mg/kg achieved tumor growth inhibition rates of 89.1% and 96.6%, respectively (**Figure 7D-G**). These findings indicated that Dextran-CTX-GLA-EuK exhibited the most potent antitumor activity, with both GLA and the targeting moiety EuK contributing to enhanced efficacy of Dextran-CTX.

Notably, in the 22Rv1 xenograft model, nude mice treated with 5 mg/kg parent CTX showed a 15.1% body weight reduction by the end of the 28-day experiment compared to baseline (day 0). In the PC-3/PSMA model, the 5 mg/kg parent CTX group exhibited a 16.7% decrease after 21 days. However, in the 10 mg/kg parent CTX group, nude mice in both models were euthanized on day 14 because their body weight decreased by 20% (**Figure 7B,7E**). Each conjugate group showed body weight higher than the initial (day 0) value, and no statistically significant inter-group differences were observed. The above results indicated that the conjugates can reduce the toxicity of the parent CTX.

**Figure 8:**
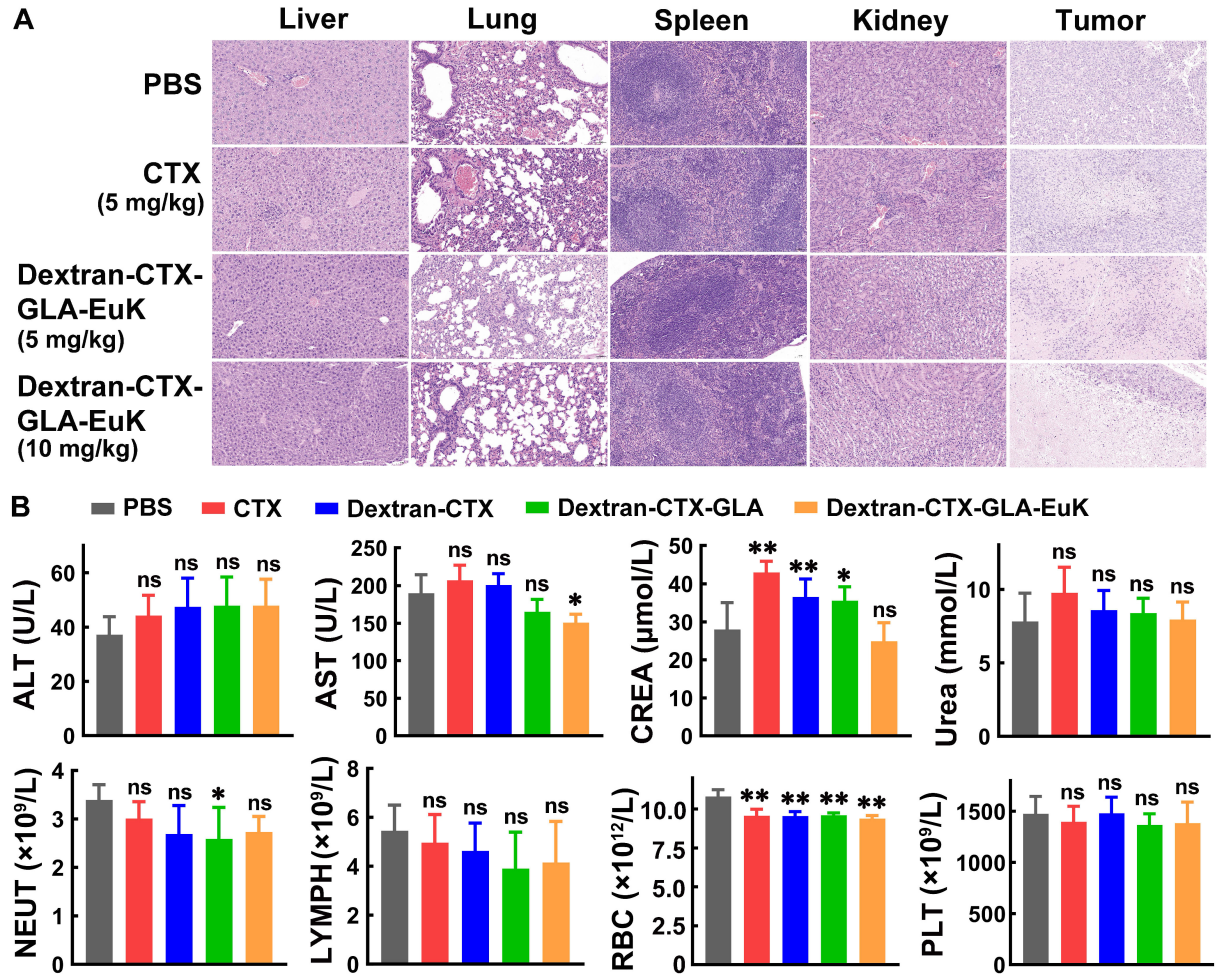
Safety evaluation of Dextran-CTX-GLA-EuK in PC-3/PSMA xenograft-bearing nude mice. (A) H&E staining of key organs in mice post-administration. (Original magnification × 200). (B) Detection of major liver function, renal function and hematological parameters. *p < 0.05, **p < 0.01, ns means p > 0.05, vs PBS control group.

At the end of the 21-day experiment in the PC-3/PSMA xenograft model, normal organs were harvested and subjected to HE staining (**Figure 8A**). No abnormalities or inflammatory cell infiltration were observed in the liver, spleen, lung, or kidney across all groups. In the tumors of each group, a large number of tumor cells showed necrosis, characterized by nuclear pyknosis, hyperchromasia, karyorrhexis, or karyolysis. Plasma samples were also collected at the end of the 21-day experiment for the determination of biochemical indicators and hematological parameter (**Figure 8B**). CTX frequently induces elevated transaminase levels (ALT and AST), and less frequently, increased creatinine. In contrast, the conjugate Dextran-CTX-GLA-EuK showed no notable differences in ALT, AST, CREA, or UREA levels compared to the PBS group. Administration of Dextran-CTX-GLA-EuK did not significantly alter the counts of NEUT, lymphocytes, RBC, or platelets. These results collectively indicated that Dextran-CTX-GLA-EuK exhibited potent antitumor activity without causing obvious toxicity to nude mice bearing PCa cells.

PSMA has emerged as a core molecular target for precision diagnosis and treatment of prostate cancer, with several PSMA-targeted radiopharmaceuticals approved for clinical use in mCRPC 15-17. However, the clinical application of these agents is hampered by two critical challenges: PSMA heterogeneity and off-target ligand uptake in normal tissues (e.g., kidneys and salivary glands) 48, 49. Notably, the conjugate Dextran-CTX-GLA-EuK addresses these limitations through distinct structural and functional advantages, which are fundamentally different from conventional PSMA-targeted radiopharmaceuticals. Firstly, with a molecular weight of ~130 kDa, Dextran-CTX-GLA-EuK passively accumulates in tumor tissues via the enhanced permeability and retention (EPR) effect, a hallmark of macromolecular anticancer agents. Biodistribution analysis in this study demonstrated that Dextran-CTX-GLA-EuK specifically accumulated in tumors with extremely low distribution in normal tissues. This inherent property minimizes the risk of off-target toxicity, as corroborated by *in vivo* studies demonstrating no observable abnormalities. Secondly, Dextran-CTX-GLA-EuK entered tumor cells through three complementary mechanisms: macropinocytosis, clathrin-dependent endocytosis, and caveolin-dependent endocytosis. Importantly, the Dextran-CTX-GLA backbone (without EuK) exhibited efficient cellular internalization even in the absence of PSMA targeting. This is because tumor cells rely heavily on macropinocytosis to scavenge extracellular macromolecules (e.g., polysaccharides, proteins) for energy metabolism and proliferation—a metabolic adaptation not shared by most normal cells 50. Consequently, tumor cells with low or absent PSMA expression can still internalize the conjugate efficiently, eliminating the risk of tumor escape associated with PSMA-targeted monotherapies. Thirdly, GLA moiety in the conjugate exerts both direct antitumor activity and synergistic effects with CTX. Critically, GLA is non-toxic to quiescent normal cells, as its antitumor effects are selectively triggered by the dysregulated metabolic microenvironment of proliferating tumor cells 51. This selectivity further mitigates potential toxicity to normal tissues, including kidneys and salivary glands. In summary, Dextran-CTX-GLA-EuK circumvents the key limitations of conventional PSMA-targeted radiopharmaceuticals by leveraging macromolecular EPR-mediated tumor targeting, multiple endocytic pathways for heterogeneous tumor cell uptake, and GLA-induced selective cytotoxicity. This study confirmed robust antitumor efficacy in xenograft models, with no detectable systemic or organ-specific toxicity. These findings highlight the conjugate’s potential as a safe and effective alternative for PCa treatment, particularly in scenarios where PSMA heterogeneity or off-target toxicity limits the utility of current PSMA-targeted therapies.

## Conclusion

A novel PSMA-targeting conjugate, Dextran-CTX-GLA-EuK, was synthesized via click chemistry in this study. The conjugate exhibited favorable physicochemical properties that make it suitable as an efficient drug delivery platform.

The combined incorporation of GLA and the PSMA-targeting EuK moiety was crucial for enhancing therapeutic efficacy. Surface plasmon resonance analysis demonstrated significantly higher PSMA binding affinity compared to non-targeted analogs, which consequently led to enhanced cellular uptake in PSMA-overexpressing cells via multiple endocytic pathways. This active targeting strategy achieved exceptional tumor accumulation *in vivo*, with free CTX and total CTX concentrations in 22Rv1 xenografts reaching 50.5-fold and 98.3-fold higher levels, respectively, than those of parent CTX control at 96 h post-administration.

Critically, Dextran-CTX-GLA-EuK exhibited potent antitumor activity superior to all comparator agents in both 22Rv1 and PC-3/PSMA xenograft models. At 10 mg/kg, it achieved remarkable 96.6% tumor growth inhibition in PC-3/PSMA tumors and significantly extended survival in 22Rv1-bearing mice. Importantly, Dextran-CTX-GLA-EuK showed improved safety profiles. Unlike parent CTX, which caused significant weight loss and even euthanasia due to 20% weight loss at high doses, all conjugate groups maintained or increased body weight. HE staining and biochemical/hematological analyses confirmed no obvious toxicity to major organs or abnormalities in immune cell counts, indicating reduced systemic toxicity.

Collectively, these findings demonstrated that Dextran-CTX-GLA-EuK integrates the advantages of dextran-based delivery, GLA-enhanced activity, and EuK-mediated PSMA targeting, resulting in synergistically enhanced antitumor efficacy and reduced toxicity. This conjugate holds promising potential as a targeted therapeutic agent for PSMA-overexpressing prostate cancer, and its design provides a valuable strategy for optimizing the delivery of chemotherapeutic drugs.

## Supplementary Material

Supplementary methods, figures.

## Figures and Tables

**Table 1:** The IC_50_ values of conjugates after 72 h incubation in cells (ng/mL)

	CTX	Dextran-CTX	Dextran-CTX-GLA	Dextran-CTX-GLA-EuK
22Rv1	4.17 ± 0.66	4.48 ± 0.84	4.84 ± 0.68	3.36 ± 0.15
LNCaP	3.52 ± 0.27	2.62 ± 0.23	2.68 ± 0.13	2.39 ± 0.23
PC-3	13.04 ± 0.23	14.95 ± 1.47	12.73 ± 1.11	13.26 ± 1.70*
PC-3/PSMA	13.47 ± 3.64	13.46 ± 2.86	9.87 ± 1.45	8.27 ± 1.91*

*P < 0.05.

## Data Availability

The data that support the findings of this study are available from the corresponding author upon reasonable request.
