# Supplementary material for: A PSMA-targeted dextran-based conjugate eradicates PSMA-overexpressing prostate tumors while abolishing cabazitaxel toxicity
Source: Theranostics. 2026 May 11;16(12):6843–60. doi: 10.7150/thno.124663 (PMC13232434; doi:10.7150/thno.124663)

Manuscript ID：124663m1

Supplementary material

A PSMA-targeted dextran-based conjugate eradicates PSMA-overexpressing prostate tumors while abolishing Cabazitaxel toxicity

Li Yang[1,#], Tingchao Liu[1,#], Meng Huo[1], Weiping Jia[1], Yu Chen[1], Maoxu Ge[2], Yan Pan[1], Jianfei Shi[3], Xiaohai Li[4,5], Si Wang[4,5], Yikang Shi[1,*]

1. National Glycoengineering Research Center, Shandong University, Qingdao 266237, China; Shandong Center of Technology Innovation for Carbohydrate, Shandong University, Qingdao 266237, China; Shandong Key Laboratory of Carbohydrate and Carbohydrate-conjugate Drugs, Shandong University, Qingdao 266237, China.

2. Department of Pharmacy, Qilu Hospital of Shandong University, Jinan 250012, China.

3. Department of Pharmacology and Pharmaceutical Sciences, Alfred E. Mann School of Pharmacy and Pharmaceutical Sciences, University of Southern California, Los Angeles, CA 90089, United States

4. Santolecan Pharmaceuticals LLC, Jupiter 33458, United States.

5. Suzhou Santolecan Pharmaceutical Co., Ltd, Suzhou 215123, China.

# These authors contributed equally to this work.

*Corresponding author. E-mail addresses: shiyikang@sdu.edu.cn (Yikang Shi).

Table of Contents

1. Figures
2. Bioanalytical method for determination of total cabazitaxel (CTX) in mouse plasma and tissues
3. Synthetic routes and procedures for conjugates
4. ^{1}H NMR, ^{13}C NMR and MS spectra of compounds related to conjugates

1. Figures

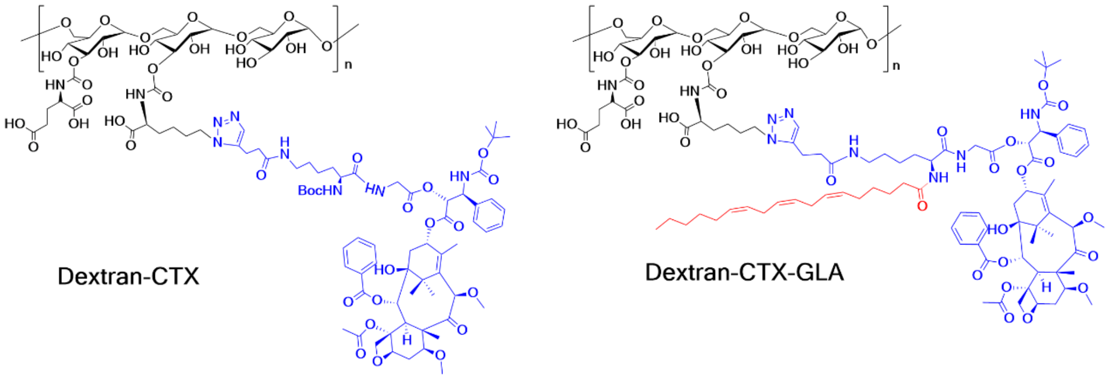

Figure S1. Structure of conjugates Dextran-CTX and Dextran-CTX-GLA

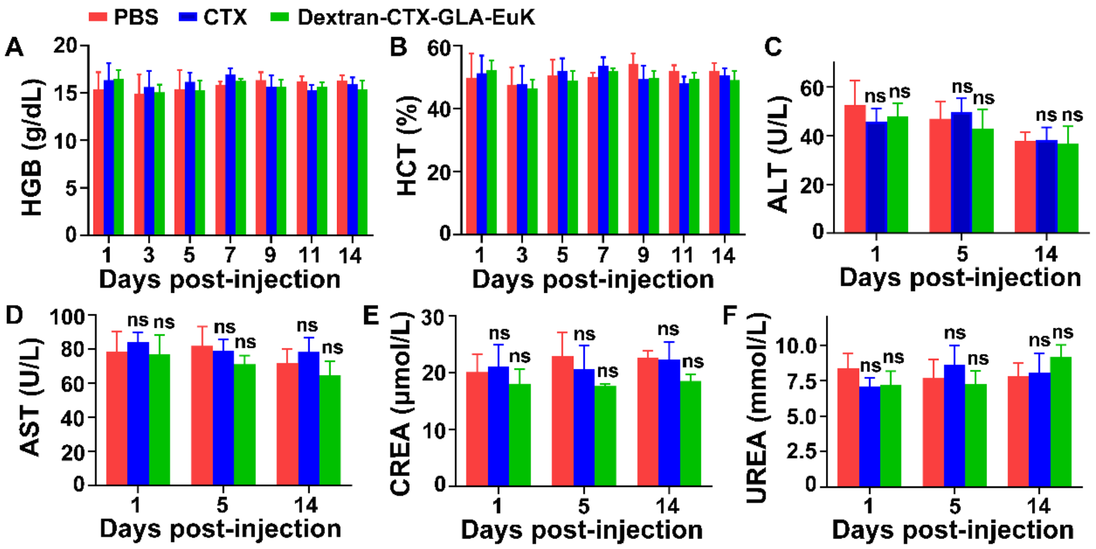

Figure S2. Myelosuppression induced by the conjugate Dextran-CTX-GLA-EuK in mice. (A-F) Effects of the conjugate (10 mg/kg) on peripheral blood HGB, HCT,

ALT, AST, CREA and UREA. ns means $p > 0.05$. ($n \geq 3$).

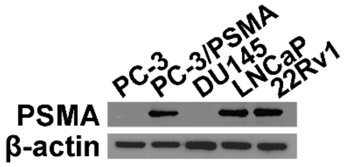

Figure S3. PSMA expression in prostate cancer cell lines by Western blot analysis.

2. Bioanalytical method for determination of total cabazitaxel (CTX) in mouse plasma and tissues

To accurately determine the total CTX concentration in mouse plasma and tissues, a sensitive, rapid, and reliable UHPLC–MS/MS bioanalytical method incorporating an alkaline hydrolysis step was developed. Plasma samples were first subjected to protein precipitation for extraction. The resulting extracts were then treated with a sodium hydroxide-based hydrolysis solution in an aqueous-organic solvent system. This hydrolysis step cleaved the ester bond at the C13 hydroxyl position of CTX, releasing a characteristic side-chain analyte that served as a surrogate marker for total CTX. The released analyte (Figure S4) was subsequently quantified using a UHPLC-MS/MS analytical method, enabling the determination of total CTX in plasma and tissues regardless of whether CTX existed in free, partially released, or conjugated forms.

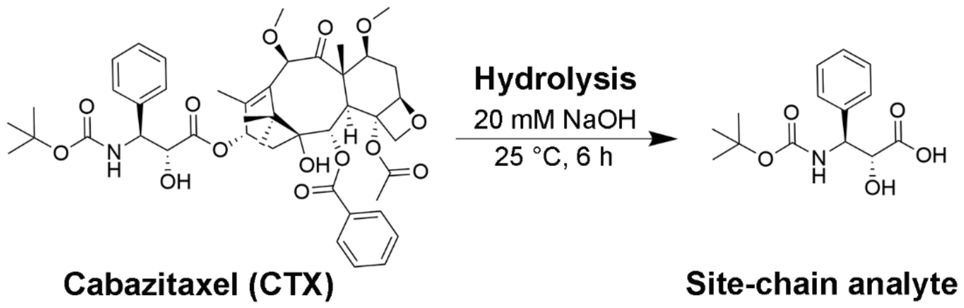

Figure S4. Schematic diagram illustrating the principle of determining the total CTX content in the conjugate Dextran-CTX-GLA-EuK via alkaline hydrolysis of CTX into its side-chain.

2.1 Reagents and Materials

Mouse plasma was used as the biological matrix for preparation of calibration standards and quality control samples. The stock solution of **side-chain reference standard** and **CTX** reference standard were made in dimethyl sulfoxide (DMSO). The test conjugate Dextran-CTX-GLA-EuK stock solution was made in **methanol–water (4:1, v/v)**. **Boc-L-Phenylalanine** was used as the internal standard (IS). The following solvents were used: Methanol, Acetonitrile, Methanol–acetonitrile (1:1, v/v) were of LC–MS grade. A 40 mM aqueous sodium hydroxide solution was prepared for the hydrolysis of extracted plasma samples.

2.2 Preparation of Stock Solutions

Stock solutions of the side-chain reference compound, **Cabazitaxel**, and the conjugate were prepared at **5 mg/mL** (calculated and converted as released side-chain analyte) in corresponding solvent.

- Side-chain standard and CTX were dissolved in **DMSO**.
- The conjugate compound was dissolved in **methanol–water (4:1, v/v)**.

Stock solutions were stored at **−20 °C** until use. Working solutions were prepared by sequentially diluting each stock solution into blank solvent **methanol–acetonitrile (1:1, v/v)**. conjugate compound working solution was prepared in **methanol–water (4:1, v/v)**.

2.3 Preparation of Calibration Standards and QC standards

Three types of calibration standards were prepared:

- Matrix calibration standards (processed samples)
- Matrix calibration standards (unprocessed samples)
- Solvent calibration standards

The dynamic range of the quantification method was 1.0–1000 ng/mL in mouse plasma. Calibration standards were prepared by spiking appropriate working solutions of the side-chain reference standard into blank mouse plasma to obtain final concentrations of 1.0, 2.0, 10, 50, 100, 200, 500, and 1000 ng/mL.

Quality control (QC) samples were prepared by separately spiking working solutions of CTX and the test conjugate Dextran-CTX-GLA-EuK into blank mouse plasma to generate nominal low, medium, and high concentration levels of 10, 100, and 500 ng/mL, respectively. The concentrations of these QC samples were calculated based on the amount of side-chain analyte formed after hydrolysis.

(i) Matrix Calibration Standards (Processed)

Aliquots of **100 µL stock solution (5 mg/mL)** were spiked into **400 µL mouse plasma**, giving a total volume of **500 µL**.

A **200 µL aliquot** of the plasma mixture was transferred and mixed with **800 µL methanol–acetonitrile (1:1, v/v)** for protein precipitation.

Samples were processed as follows:

- Vortex mixing for **10 s**
- Incubation at **room temperature for 10 min**
- Centrifugation at **13,500 g for 15 min at 15 °C**

The supernatant, in which the side-chain compound was present at a concentration of **200 µg/mL**, was collected as the **intermediate solution**.

Serial dilutions were prepared using **methanol–acetonitrile (1:1, v/v)** to obtain calibration standards across the desired concentration range.

An equal volume of **internal standard solution containing Boc-L-Phenylalanine in acetonitrile-40 mM NaOH solution (1:1, v/v)** was added to each calibration sample.

(ii) Matrix Calibration Standards (Unprocessed)

Stock solutions (5 mg/mL) were spiked into **blank plasma** by adding **100 µL stock solution to 400 µL plasma**, yielding a **500 µL mixture**.

The mixture was then serially diluted with **methanol–acetonitrile (1:1, v/v)** to prepare calibration standards at different concentrations.

Each standard was mixed with an equal volume of internal standard solution containing **Boc-L-Phenylalanine in acetonitrile–40 mM NaOH aqueous solution (1:1, v/v)**.

(iii) Solvent Calibration Standards

Solvent calibration standards were prepared using **methanol–acetonitrile (1:1, v/v)** as the blank solvent.

Stock solutions (5 mg/mL) of the side-chain compound, **Cabazitaxel**, and conjugate were serially diluted with methanol–acetonitrile (1:1, v/v) to produce a series of standard concentrations.

Each calibration solution was mixed with an equal volume of internal standard solution containing **Boc-L-Phenylalanine in acetonitrile–40 mM NaOH aqueous solution (1:1, v/v)**.

2.4 Plasma extraction Preparation

Mouse plasma samples were processed using **protein precipitation extraction**. 20 uL standard samples were added with 80 uL **methanol–acetonitrile (1:1, v/v)**, samples in eppendorf vials were vortexed for 10 seconds at 4000 rpm, followed by centrifugation at 13,500 g for 15 min at 15 °C. An aliquot of the resulting supernatant was carefully transferred into another new vials and prepared for further hydrolysis process.

2.5 Incubation Procedure for Total CTX Determination

To determine **total CTX from mice plasma samples**, all prepared samples were subjected to protein precipitation extraction followed by hydrolysis treatment on extracted plasma samples.

Samples were incubated under the following conditions:

- **Temperature:** 25 °C
- **Shaking speed:** 200 rpm
- **Incubation time:** 6 h

Following the incubation step, the samples were subjected to centrifugation at **13,500 g for 15 min at 4 °C**. The supernatant obtained from this step was harvested and prepared for LC-MS analysis.

2.6 LC–MS Analysis

Quantification was conducted by means of high-performance liquid

chromatography–mass spectrometry (HPLC-MS), and the analytical method was validated to determine total CTX concentration generated from the conjugate after incubation. Peak area ratios of analyte to internal standard (Boc-L-Phenylalanine) were used to construct calibration curves.

An aliquot of 2.0 µL of each prepared sample was injected into an Agilent UHPLC system coupled with a SCIEX QTRAP 5500 mass spectrometer (Sciex, Framingham, MA, USA). Separation of the side-chain analyte and its internal standard (IS) was achieved on an Agilent column (3.5 µm, 2.1 × 100 mm) with the column temperature set to 40 °C. The mobile phase comprised solvent A (0.1% formic acid in water) and solvent B (0.1% formic acid in acetonitrile). Gradient elution was applied as follows: 45% B from 0 to 1.8 min increasing to 95% B, maintained at 95% B for 1.0 min, followed by a rapid decrease to 45% B within 0.2 min, and column equilibration at 45% B until 4.5 min. The mobile phase was delivered at a constant flow rate of 0.40 mL/min. The retention times of the side-chain analyte and the internal standard, BOC-L-phenylalanine, were 1.6 and 1.91 min, respectively.

For quantitative analysis, the mass spectrometer was operated in negative electrospray ionization (ESI) mode using selected reaction monitoring (SRM). The transition m/z 280.2 → 206.2 was used for quantification of the side-chain analyte, while the transition m/z 264.2 → 190.0 was used for the internal standard. The ESI source temperature was set at 550 °C, and the ion spray voltage was −4000 V. The nebulizing gas and desolvation gas were set at 55 and 45 psi, respectively, while the curtain gas was maintained at 20 psi. The collision energies were −17 V and −14 V, the declustering potentials were −60 V and −50 V, and the entrance potential was 10 V for both transitions. The dwell time for each transition was 100 ms.

Data acquisition and processing were performed using Analyst software (version 1.6.2, SCIEX). Calibration curves were generated by plotting the peak area ratios of analyte to internal standard versus nominal analyte concentrations using a weighted (1/x) linear regression model.

2.7 Data Analysis

Calibration curves were generated by plotting the peak area ratio of analyte/internal

standard versus nominal concentration. Quantification of unknown samples was performed using the calibration curve obtained from matrix standards.

3. Synthetic Routes and Procedures for Conjugates

3.1 Synthetic scheme 1: Synthesis of Bifunctionalized dextran (4)

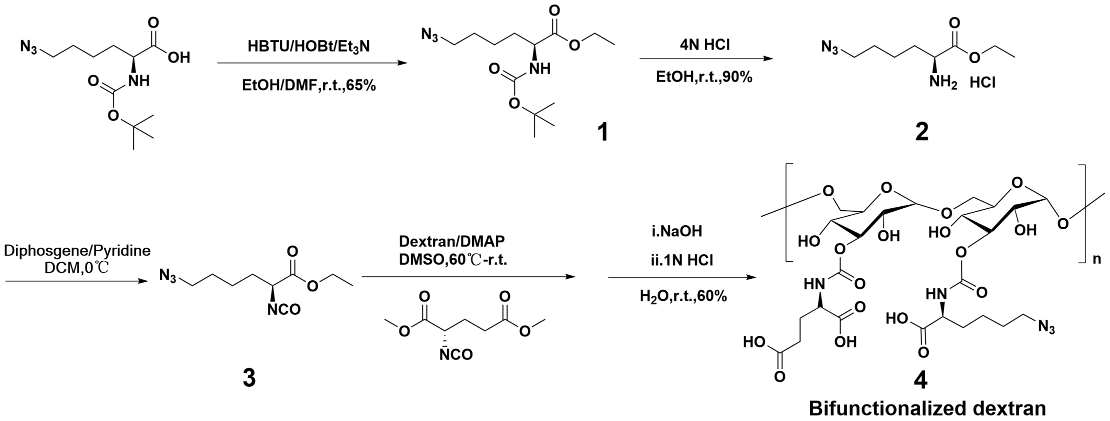

Scheme S1. Synthetic pathway of Bifunctionalized dextran.

3.1.1 Preparation of compound 1

Boc-Lys(N_3)-OH (2 g, 7.3 mmol), HBTU (4.18 g, 11 mmol), and HOBt (1.7 g, 11 mmol) were dissolved in anhydrous N,N-dimethylformamide (DMF, 15 mL) within a 250-mL round-bottom flask. Triethylamine (2 mL, 14.7 mmol) was introduced dropwise, followed by stirring at ambient temperature for 30 min. Subsequently, 10 mL of anhydrous ethanol was added, and the reaction was permitted to proceed at ambient temperature for 3 h, monitored by TLC (pE:EA = 2:1). Upon reaction completion, the mixture was extracted with ethyl acetate and brine (100 mL each). Subquently the organic layer was washed with brine twice (50 mL × 2), dried over anhydrous sodium sulfate, filtered, and concentrated under reduced pressure. Subsequent purification via silica gel column chromatography, eluting with a gradient of ethyl acetate in petroleum ether (10-50%), afforded 1.4g of compound **1**, corresponding to a yield of 70%.

^{1}H NMR (400 MHz，DMSO)：δ7.23 (d, J = 8.0 Hz, 1H), 4.15–4.01 (m, 2H), 3.90 (m, 1H), 3.30 (d, J = 6.8 Hz, 2H), 1.62 (m, 2H), 1.38 (s, 13H), 1.34 (s, 2H), 1.18 (t, J = 7.1 Hz, 3H).

^{13}C NMR (100 MHz，DMSO)：δ173.34, 173.03, 156.04, 78.88, 78.63, 78.59, 60.72, 55.19, 53.97, 53.81, 52.15, 50.97, 30.68, 30.63, 28.61, 28.35, 28.31, 23.31, 14.53.

MS (ESI, m/z)：calcd for $C_{13}H_{24}N_4O_4$ $[M+Na]^+$:323.36; found:323.24.

3.1.2 Preparation of compound 2

Compound **1** (1.4 g, 4.33 mmol) was dissolved in 15 mL of 4.0 N HCl in ethanol within a 250 mL round-bottom flask, and the mixture was stirred at ambient temperature for 1.5 h. After concentration, the reaction mixture was purified via silica gel column chromatography eluting with a gradient of methanol in chloroform (1% – 8%), affording 827 mg of the desired compound **2** in 90% yield.

^{1}H NMR (400 MHz，DMSO)：δ8.55 (s, 2H), 4.29–4.14 (m, 2H), 3.94 (t, J = 6.3 Hz, 1H), 1.88–1.77 (m, 2H), 1.54 (m, 2H), 1.49–1.30 (m, 2H), 1.24 (t, J = 7.1 Hz, 3H).

^{13}C NMR (100 MHz，DMSO)：δ170.11, 62.10, 52.20, 50.76, 30.08, 28.18, 22.01, 14.42.

MS (ESI, m/z)：calcd for $C_8H_{16}N_4O_2$ $[M+H]^+$:201.13;found:201.16.

3.1.3 Preparation of Bifunctionalized dextran (4)

To a 250-mL round-bottom flask was added compound **2** (2 g, 10 mmol), which was dissolved in anhydrous DCM and pyridine (4 g, 50 mmol) under nitrogen protection. After cooling to 0 ℃, diphosgene (2.4 g, 6 mmol) dissolved in anhydrous DCM was introduced slowly, and the resulting mixture was stirred for 5 h. After dilution with DCM (100 mL), the mixture was washed with 1.0 N HCl (50 mL × 3), dried over anhydrous Na_2SO_4, filtered, and evaporated to dryness to yield 1.5 g of compound **3**. Owing to its instability, this compound was employed directly in the subsequent step without further purification.

A total of 2 g of dextran (average molecular weight: 100 kDa) was completely dried under high vacuum in a 60 °C oil bath for 10 h, followed by dissolution in 10 mL of anhydrous DMSO at the same temperature. Once the mixture had cooled to ambient temperature, the oil bath was taken away. To the above mixture, (s)-dimethyl 2-isocyanoglutarate (4.14 g, 20.6 mmol), compound **3** (1.5 g, 6.64 mmol) and DMAP (10 g, 81.72 mmol) were introduced gradually and stirred overnight at ambient temperature. Following the end of the reaction, the resulting solution was subjected to direct dialysis against distilled water for 24 h, after which it was concentrated and treated with NaOH (4.36 g, 109 mmol) for hydrolysis for a period of 5 h. The pH of the mixture was adjusted to 3.0-5.0 with 4 N HCl solution, followed by repeated dialysis against distilled water three times, concentration, and lyophilization to afford **bifunctionalized dextran (4)** (1.5 g).

^{1}H NMR (400 MHz, DMSO) δ 5.98 (t, J = 2.1 Hz, 1H), 4.81 (s, 3H), 4.39 (s, 2H),

4.04 (s, 32H), 3.70 (s, 4H), 3.30 (t, *J* = 6.7 Hz, 7H), 2.55 (s, 14H), 2.32 (q, *J* = 9.1 Hz, 13H), 2.06 – 1.90 (m, 6H), 1.81 (s, 9H), 1.62 – 1.49 (m, 5H), 1.39 (s, 4H).

3.2 Synthetic scheme 2: Preparation of CTX-GLA-linker A (10)

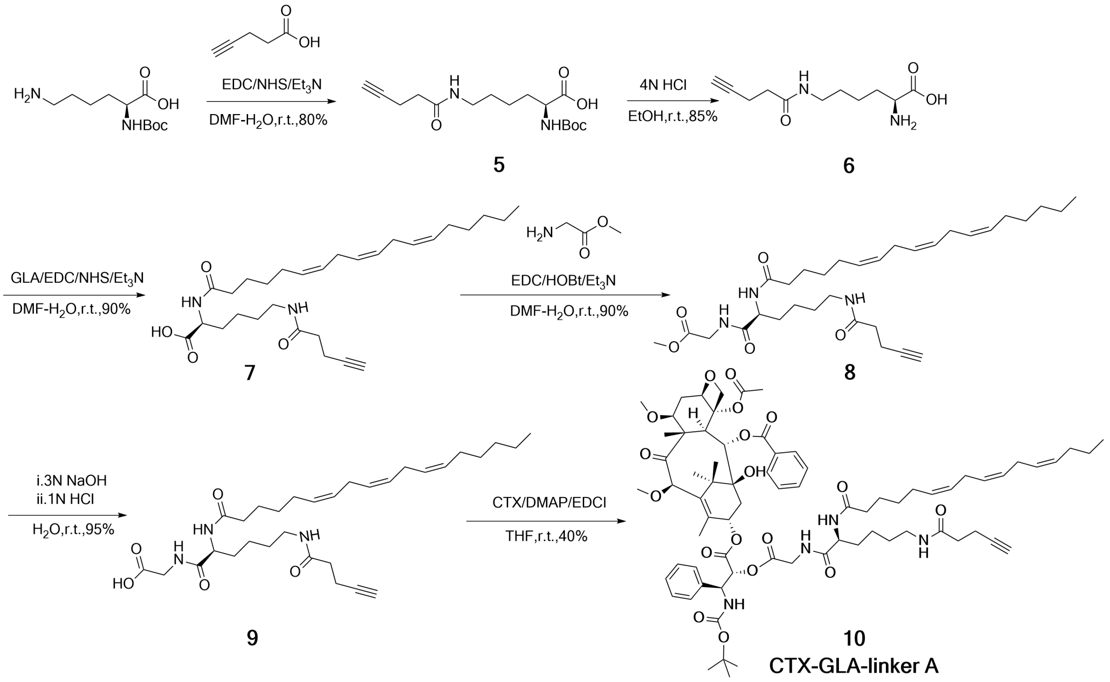

Scheme S2. Synthetic route of CTX-GLA-linker A

3.2.1 Preparation of compound 5

To 30 mL of anhydrous methylene chloride were added 4-pentynoic acid (2 g, 20.4 mmol), EDCI (5.9 g, 30.7 mmol), and NHS (3.52 g, 30.6 mmol), and the solution was stirred at ambient temperature for 3 h. Once the reaction finished, the solution was diluted with DCM (100 mL), washed with brine three times, dried over anhydrous Na_2SO_4, filtered, and evaporated to dryness. After dissolving N6-Boc-Linker-lysine (LL-1, 9.5 g, 38.4 mmol) in 30 mL DMF, triethylamine (5.4 mL, 38.4 mmol) and the above freshly prepared 4-pentynoic acid NHS ester in anhydrous DMF were introduced, followed by stirring at ambient temperature for 3 h. Following completion of the reaction, the resulting mixture was partitioned with ethyl acetate (100 mL) and brine (100 mL). The organic layer was washed twice with brine, dried over anhydrous Na_2SO_4, filtered, and concentrated. Further purification via silica gel column chromatography with 2-7% methanol in dichloromethane yielded compound 5 (6.6 g) with an 80% yield.

^{1}H NMR (400 MHz，DMSO)：δ12.42 (s, 1H), 7.86 (t, *J* = 5.6 Hz, 1H), 7.01 (d, *J* = 8.0 Hz, 1H), 3.82 (m, 1H), 3.02 (q, *J* = 6.3 Hz, 2H), 2.73 (t, *J* = 2.6 Hz, 1H), 2.37–2.31 (m, 2H), 2.27–2.21 (m, 2H), 1.66–1.49 (m, 2H), 1.38 (s, 9H), 1.35 (d, *J* = 4.7 Hz,

2H), 1.30 (m, 2H).

^{13}C NMR (100 MHz，DMSO)：δ175.00, 174.74, 170.51, 156.08, 84.23, 78.42, 71.70, 55.16, 53.88, 38.68, 34.71, 30.86, 29.17, 28.67, 28.42, 23.50, 14.77.

MS (ESI, m/z)：calcd for $C_{16}H_{26}N_2O_5$ $[M\text{-}H]^-$:325.1768; found:325.1771.

3.2.2 Preparation of compound 6

Compound **5** (6 g, 18.5 mmol) was taken up in 20 mL of a 3.0 N HCl solution in ethanol and stirred at ambient temperature for 2 h. After the reaction was finished, the mixture was concentrated and subjected to silica gel column chromatography using a gradient of methanol in dichloromethane (2-30%) as the eluent, affording compound **6** (3.6 g) in 85% yield.

^{1}H NMR (400 MHz, DMSO)：δ8.50 (s, 3H), 8.06 (t, *J* = 5.6 Hz, 1H), 3.86–3.78 (m, 1H), 3.03 (q, *J* = 6.3 Hz, 2H), 2.77 (t, *J* = 2.5 Hz, 1H), 2.41–2.31 (m, 2H), 2.27 (m, 2H), 1.86–1.73 (m, 2H), 1.47–1.22 (m, 4H).

^{13}C NMR (100 MHz, DMSO)：δ171.40, 170.58, 84.25, 71.78, 65.50, 62.15, 52.24, 38.50, 38.42, 34.68, 34.42, 29.98, 28.91, 22.11, 14.76, 14.43.

MS (ESI, m/z)：calcd for $C_{11}H_{18}N_2O_3$ $[M+H]^+$:227.13; found:227.12.

3.2.3 Preparation of compound 7

Anhydrous dichloromethane (20 mL) was used to dissolve GLA (1.5 g, 5.38 mmol), NHS (1.9 mg, 16.2 mmol), and EDCI (3.1 g, 16.2 mmol), and the resulting solution was stirred at ambient temperature for 2 h. After the reaction was finished, the mixture was taken up in dichloromethane (100 mL), washed with three portions of brine (100 mL each), dried over anhydrous Na_2SO_4, filtered, and concentrated to dryness. In the next step, a solution of compound **6** (2.5 g, 11.1 mmol) in DMF (30 mL) was treated with triethylamine (5.2 mL, 37 mmol) and the above-obtained GLA-NHS ester (dissolved in anhydrous DMF), and the resulting mixture was stirred at ambient temperature for 3 h. Following completion of the reaction, 1 N HCl was added to adjust the pH to 4.0-5.0. The mixture was then taken up in ethyl acetate (100 mL) and brine (100 mL), and the separated organic layer was washed with brine (100 mL × 2), dried over anhydrous Na_2SO_4, filtered, and concentrated. Column chromatography on silica gel (eluent: 2-11% methanol in dichloromethane) gave compound **7** (3.26 g) with a 90% yield.

^{1}H NMR (400 MHz, DMSO)：δ5.40–5.27 (m, 6H), 4.17–4.10 (m, 1H), 3.05–2.98 (m, 2H), 2.78 (t, *J* = 5.8 Hz, 4H), 2.73 (t, *J* = 2.6 Hz, 1H), 2.34 (m, 2H), 2.27–2.22 (m, 2H), 2.12 (m, 2H), 2.08–1.99 (m, 4H), 1.60–1.44 (m, 4H), 1.33–1.25 (m, 10H), 0.88–0.84 (m, 3H).

^{13}C NMR (100 MHz, DMSO)：δ174.37, 172.65, 170.49, 130.39, 130.20, 128.42, 128.36, 128.14, 128.02, 84.20, 71.69, 52.11, 38.71, 35.36, 34.70, 31.36, 31.19, 29.49, 29.19, 29.16, 29.09, 27.08, 26.95, 25.68, 25.40, 23.34, 22.45, 14.76, 14.40.

MS (ESI, m/z)：calcd for $C_{29}H_{46}N_2O_4$ [M-H]$^-$:485.34; found:485.41.

3.2.4 Preparation of compound 8

Anhydrous DMF (10 mL) was used to combine glycine methyl ester hydrochloride (2.3 g, 18.5 mmol) and triethylamine (3.5 mL, 24.7 mmol). The mixture was heated at 3°C for 1 h, and then cooled to room temperature. Subsequently, compound **7** (3 g, 6.17 mmol), EDCI (2.4 g, 12.34 mmol), and HOBt (1.7 g, 12.34 mmol) were added, and the resulting mixture was stirred at room temperature overnight. Upon completion, the mixture was extracted between ethyl acetate (100 mL) and brine (100 mL). The organic layer was washed twice with brine (50 mL each), dried over anhydrous Na_2SO_4, filtered, and concentrated. Purification by silica gel column chromatography using a gradient of methanol in dichloromethane (2 – 8%) as the eluent afforded compound **8** (3.1 g) in 90% yield .

^{1}H NMR (400 MHz, DMSO)：δ8.31 (t, *J* = 5.9 Hz, 1H), 7.90 (d, *J* = 8.2 Hz, 1H), 7.84 (t, *J* = 5.6 Hz, 1H), 5.42–5.25 (m, 6H), 4.25 (m, 1H), 3.91–3.74 (m, 2H), 3.62 (s, 3H), 3.01 (m, 2H), 2.78 (t, *J* = 5.8 Hz, 4H), 2.74 (d, *J* = 2.5 Hz, 1H), 2.39–2.29 (m, 2H), 2.28–2.19 (m, 2H), 2.13 (m, 2H), 2.03 (q, *J* = 7.2 Hz, 4H), 1.68–1.58 (m, 1H), 1.55–1.43 (m, 3H), 1.41–1.34 (m, 2H), 1.33–1.21 (m, 10H), 0.88–0.82 (m, 3H).

^{13}C NMR (100 MHz, DMSO)：δ172.92, 172.52, 170.71, 170.46, 130.40, 130.22, 128.43, 128.36, 128.13, 128.02, 84.22, 71.71, 52.45, 52.12, 40.96, 38.83, 35.46, 34.70, 32.18, 31.35, 29.19, 29.16, 27.07, 26.95, 25.68, 25.37, 23.14, 22.45, 14.76, 14.40.

MS (ESI, m/z)：calcd for $C_{32}H_{51}N_3O_5$ [M+H]$^+$:558.39; found:558.96.

3.2.5 Preparation of compound 9

A solution of compound **8** (3 g, 5.38 mmol) in 10 mL of methanol was prepared, to which 3 N NaOH was added, and the mixture was stirred at ambient temperature for 1 h. After the reaction was completed, the pH of the resulting solution was brought to 3.0-5.0 by the addition of 1.0 N HCl. The mixture was separated between ethyl acetate (100 mL) and brine (100 mL), after which the organic layer was rinsed with brine twice using 50 mL each time. After drying over anhydrous sodium sulfate, filtration, and evaporation, the crude product was subjected to silica gel column chromatography, eluting with a gradient of 2 – 20% methanol in methylene chloride, yielding compound **9** (2.77 g) with a 95% yield.

^{1}H NMR(400 MHz, DMSO)：δ7.98 (d, *J* = 8.2 Hz, 1H), 7.88 (m, 2H), 5.43–5.26 (m, 6H), 4.23 (m, 1H), 3.61 (t, *J* = 4.8 Hz, 2H), 3.00 (m, 2H), 2.78 (s, 4H), 2.74 (t, *J* =

2.6 Hz, 1H), 2.34 (m, 2H), 2.24 (m, 2H), 2.14(h, J = 6.8 Hz, 2H), 2.03 (m, 4H), 1.68–1.57 (m, 1H), 1.50 (m, 3H), 1.40–1.35 (m, 2H), 1.32–1.21 (m, 10H), 0.88–0.82 (m, 3H).

^{13}C NMR (100 MHz, DMSO)：δ172.58, 172.16, 170.48, 132.00, 130.39, 130.22, 129.13, 128.43, 128.36, 128.12, 128.02, 84.21, 71.71, 65.49, 52.72, 42.39, 38.80, 35.50, 34.69, 32.14, 31.35, 30.47, 29.48, 29.24, 29.19, 27.07, 26.95, 25.68, 25.39, 23.24, 22.45, 19.13, 14.77, 14.40, 14.02.

MS (ESI, m/z)：calcd for $C_{31}H_{49}N_3O_5$ [M-H]$^-$:542.36; found:542.37.

3.2.6 Preparation of CTX-GLA-linker A (10)

To 10 mL of anhydrous DMF were added compound **9** (2.5 g, 4.6 mmol), DMAP (468 mg, 3.83 mmol), and EDCI (1.47 g, 7.66 mmol). After stirring for 40 min, CTX (3.2 g, 3.83 mmol) was introduced, and the solution was stirred at ambient temperature for 3 h. Once the reaction finished, the resulting mixture was separated between ethyl acetate (100 mL) and brine (100 mL). After washing the organic layer twice with brine, drying over anhydrous Na_2SO_4, filtration, and concentration, the residue was purified by silica gel column chromatography eluted with 1-6% methanol in methylene chloride to yield 2 g of **CTX-GLA-linker A (10)**, with a yield of 40%.

^{1}H NMR (400 MHz, DMSO)：δ8.40–8.32 (m, 1H), 7.97 (dd, J = 8.8, 7.1 Hz, 2H), 7.84 (t, J = 5.7 Hz, 1H), 7.73 (dd, J = 8.4, 6.2 Hz, 1H), 7.66 (dd, J = 8.2, 6.7 Hz, 2H), 7.47–7.29 (m, 4H), 7.19 (t, J = 7.3 Hz, 1H), 5.84 (t, J = 9.1 Hz, 1H), 5.38–5.29 (m, 6H), 5.10 (d, J = 7.7 Hz, 2H), 4.98–4.90 (m, 1H), 4.68 (s, 1H), 4.52 (s, 1H), 4.27 (m, 1H), 4.04–3.96 (m, 3H), 3.74 (dd, J = 10.5, 6.7 Hz, 1H), 3.58 (d, J = 7.1 Hz, 1H), 3.28 (s, 3H), 3.20 (s, 3H), 3.01 (q, J = 6.4 Hz, 2H), 2.89 (s, 1H), 2.77 (t, J = 5.8 Hz, 3H), 2.75–2.72 (m, 2H), 2.37–2.29 (m, 2H), 2.28–2.19 (m, 5H), 2.18–2.08 (m, 2H), 2.03 (m, 4H), 1.78 (s, 3H), 1.60 (p, J = 6.9 Hz, 2H), 1.49 (d, J = 7.9 Hz, 6H), 1.39 (s, 9H), 1.33–1.22 (m, 13H), 0.98 (d, J = 7.6 Hz, 6H), 0.85 (t, J = 6.8 Hz, 3H).

^{13}C NMR (100 MHz, DMSO)：δ205.10, 172.94, 172.65, 170.44, 170.15, 169.72, 169.26, 165.71, 162.78, 155.70, 138.94, 137.80, 135.40, 133.96, 130.40, 130.37, 130.20, 130.06, 129.17, 129.10, 128.57, 128.44, 128.35, 128.15, 128.01, 127.85, 84.19, 83.71, 82.48, 80.78, 80.63, 79.07, 77.17, 75.70, 74.79, 71.70, 71.56, 57.10, 56.97, 56.45, 55.54, 52.51, 46.84, 43.40, 38.78, 36.25, 35.49, 34.90, 34.70, 32.11, 31.35, 31.24, 29.50, 29.31, 29.18, 28.58, 27.07, 26.94, 25.68, 25.34, 23.21, 23.01, 22.57, 22.45, 21.58, 14.75, 14.54, 14.43, 14.40, 10.59.

MS (ESI,m/z)：calcd for $C_{76}H_{104}N_4O_{18}$ [M+H]$^+$:1361.74; found:1361.72.

3.3 Synthetic scheme 3: Preparation of EuK-linker B (20), a targeted molecule for PSMA receptors

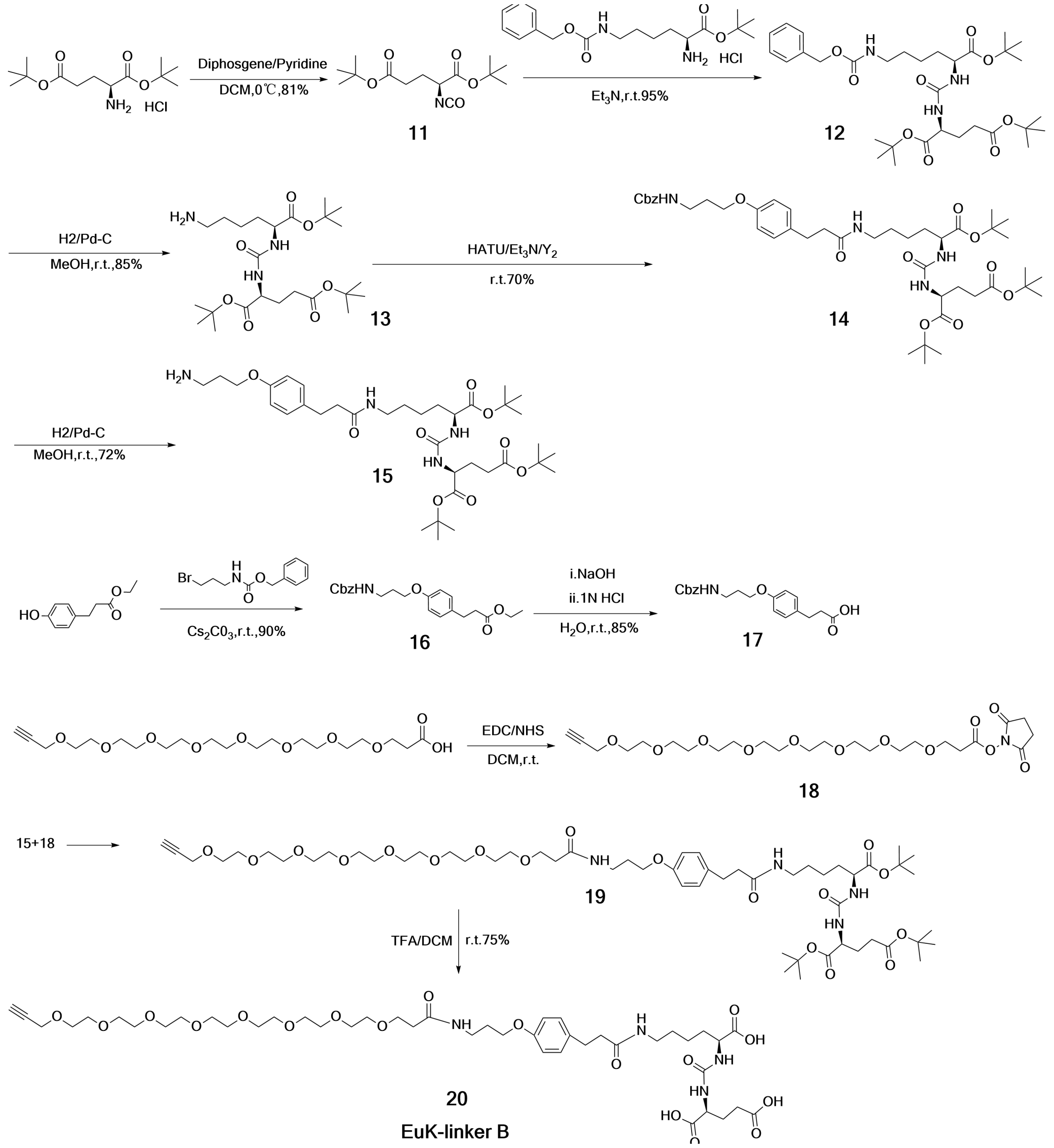

Scheme S3. Synthetic route of EuK-linker B

3.3.1 Preparation of compound 11

L-Glutamic acid di-tert-butyl ester hydrochloride (2 g, 6.8 mmol) was placed in a 250 mL round-bottom flask and dissolved in anhydrous DCM and pyridine (2.74 g, 34 mmol) under nitrogen protection. The solution was cooled to 0ºC, followed by the gradual addition of diphosgene (1.6 g, 8.1 mmol) dissolved in anhydrous DCM, and stirring was maintained for 5 h. Once the reaction finished, the solution was adjusted to pH 3.0-5.0 using 1.0 N HCl. Partitioning of the mixture between DCM (100 mL) and brine (100 mL) was followed by washing the organic phase with brine (25 mL×2),

drying over anhydrous sodium sulfate, filtration, and concentration to afford the crude product. Analytical TLC of compound **11** showed no detectable impurities. It was therefore used directly in the subsequent reaction.

3.3.2 Preparation of compound 12

Compound **11** (1.5 g, 5.26 mmol) was placed in a 250 mL round-bottom flask, followed by dissolution of H-Lys(Z)-OtBu (1.63 g, 4.45 mmol) in anhydrous DCM. Triethylamine (1.84 mL) was then added dropwise, followed by stirring for 3 h. After the reaction was completed, the resulting mixture was separated between ethyl acetate (100 mL) and brine (100 mL). After washing the organic layer twice with brine, drying over anhydrous Na_2SO_4, filtration, and concentration, the residue was purified by silica gel column chromatography eluted with 1-5% methanol in methylene chloride to yield 2.6 g of compound 12 (95% yield).

^1H NMR (400 MHz, $CDCl_3$)：δ7.40–7.29 (m, 5H), 5.17 (d, J = 8.0 Hz, 2H), 4.33 (m, 2H), 3.18 (d, J = 7.4 Hz, 2H), 2.36–2.23 (m, 2H), 2.06 (q, J = 5.7 Hz, 2H), 1.88–1.75 (m, 2H), 1.52 (s, 2H), 1.44 (d, J = 7.0 Hz, 27H), 1.30–1.20 (m, 2H).

^{13}C NMR (100 MHz, DMSO)：δ172.71, 172.36, 171.88, 157.56, 156.53, 137.73, 128.80, 128.19, 81.04, 80.74, 80.19, 65.56, 53.49, 52.60, 40.53, 32.21, 31.32, 29.53, 28.18, 28.09, 22.79.

MS (ESI, m/z)：calcd for $C_{32}H_{51}N_3O_9$ $[M+H]^+$:622.36; found:622.14.

3.3.3 Preparation of compound 13

In a 50 ml double-mouth bottle, compound **12** was dissolved in methanol, and palladium carbon (palladium 10% on Carbon) was gently added after complete dissolution. Be sure to remove air from the device and check air tightness. Remove the surrounding combustible material and connect hydrogen at one end of the device. The reaction lasted for 2 h.TLC analysis using a DCM/MeOH (9:1, v/v) mixture was performed to track the reaction progress. Upon completion of the hydrogenation, the hydrogen atmosphere was gently released before removing the reaction flask. After filtration, the resulting pale green filtrate was concentrated under reduced pressure, and the product was dried completely in vacuo. Silica gel column chromatography of the crude residue with 2-15% methanol in methylene chloride furnished 1.67 g of compound **13**, with an isolated yield of 85%.

^1H NMR (400 MHz, DMSO)：δ6.32 (dd, J = 16.5, 8.3 Hz, 2H), 3.99 (m, 2H), 3.17 (s, 2H), 2.29–2.15 (m, 2H), 1.91–1.81 (m, 1H), 1.67 (m, 1H), 1.55 (m, 2H), 1.41–1.35 (m, 29H), 1.34–1.29 (m, 2H).

^{13}C NMR (100 MHz, DMSO)：δ172.79, 172.39, 171.90, 157.60, 81.02, 80.69, 80.20, 53.52, 52.61, 49.06, 41.73, 33.03, 32.36, 31.34, 28.19, 28.12, 28.09, 28.03, 22.87.

MS (ESI, m/z)：calcd for $C_{24}H_{45}N_3O_7$ $[M+H]^+$:488.33; found:488.39.

3.3.4 Preparation of compound 14

In a 250 mL round-bottom flask containing 2 g (5.6 mmol) of compound **17** and 2.2 g (5.8 mmol) of HATU dissolved in DMF, 1 mL (7.46 mmol) of triethylamine was introduced, followed by stirring at ambient temperature for 30 min. Subsequently, compound 13 was added, and the mixture was stirred for an additional 3 h at room temperature. After the reaction reached completion, the mixture was partitioned between 100 mL of ethyl acetate and 100 mL of brine. The organic layer was then washed twice with 100 mL portions of brine, dried over anhydrous sodium sulfate, filtered, concentrated, and purified via silica gel column chromatography using a gradient of 0-4% methanol in dichloromethane as the eluent. This process yielded 2.2 g of the target compound **14**, corresponding to a 70% yield.

^{1}H NMR (400 MHz, DMSO)：δ7.78 (d, *J* = 5.8 Hz, 1H), 7.34 (d, *J* = 6.6 Hz, 5H), 7.08 (dd, *J* = 8.6, 2.1 Hz, 2H), 6.80 (dd, *J* = 8.5, 2.1 Hz, 2H), 6.28 (m, 2H), 5.01 (d, *J* = 2.0 Hz, 2H), 4.09–3.90 (m, 4H), 3.16 (t, *J* = 6.7 Hz, 2H), 3.01 (t, *J* = 6.7 Hz, 2H), 2.79–2.62 (m, 4H), 2.37–2.14 (m, 5H), 1.89–1.82 (m, 3H), 1.74–1.50 (m, 4H), 1.39 (d, *J* = 2.3 Hz, 27H), 1.25 (d, *J* = 8.5 Hz, 2H).

^{13}C NMR (100 MHz, DMSO)：δ172.75, 172.37, 171.90, 171.61, 157.58, 157.24, 156.61, 137.70, 133.72, 129.64, 129.56, 128.82, 128.23, 114.68, 81.06, 80.75, 80.21, 65.64, 65.44, 53.48, 52.59, 38.67, 37.91, 37.82, 32.20, 31.33, 30.79, 29.66, 29.26, 28.18, 28.09, 22.96.

MS (ESI, m/z)：calcd for $C_{44}H_{66}N_4O_{11}$ $[M+H]^+$:827.47; found:827.42.

3.3.5 Preparation of compound 15

Compound **14** was subjected to hydrogenolysis to remove the Cbz protecting group, yielding compound **15**. This hydrogenation was performed under identical conditions to those previously described for the hydrogenation step of compound **13**. Following the reaction, the mixture was concentrated in vacuo and the residue was further dried under high vacuum. Purified on a silica gel column eluted with methanol in methylene chloride (2-15 %) to offer 1.2 g of compound **15**. Yield: 72%.

^{1}H NMR (400 MHz, DMSO)：δ7.77 (t, *J* = 5.7 Hz, 1H), 7.09 (d, *J* = 8. 5 Hz, 2H), 6.82 (d, *J* = 8.5 Hz, 2H), 6.28 (dd, *J* = 13.7, 8.3 Hz, 2H), 4.06–

3.92 (m, 4H), 3.00 (d, *J* = 6.9 Hz, 2H), 2.86 (t, *J* = 7.2 Hz, 2H), 2.72 (t, *J* = 7.8 Hz, 2H), 2.33–2.17 (m, 6H), 1.90 (q, *J* = 6.1 Hz, 2H), 1.71–1.54 (m, 2 H), 1.39 (d, *J* = 3.0 Hz, 31H), 1.24 (s, 2H).

^{13}C NMR (100 MHz, DMSO)：δ172.75, 172.37, 171.90, 171.60, 157.59, 157.06, 133.90, 129.59, 129.37, 128.68, 125.79, 114.69, 81.06, 80.75, 80.22, 65.13, 53.49, 52.59, 38.66, 37.88, 37.38, 32.19, 31.32, 30.76, 29.25, 28.85, 28.18, 28.10, 28.06, 22.97, 21.52.

MS (ESI, m/z)：calcd for $C_{36}H_{60}N_4O_9$ $[M+H]^+$:693.43; found:693.53.

3.3.6 Preparation of compound 16

Ethyl p-hydroxycinnamate (2.0 g, 10.3 mmol), benzyl 3-bromopropylcarbamate (3.4 g, 12.4 mmol), and Cs_2CO_3 (10.0 g, 30.9 mmol) were combined in anhydrous DMF (100 mL). The reaction mixture was heated at 60°C for 5 h, with its progress monitored using thin-layer chromatography (TLC). Once the reaction was deemed complete, the mixture was partitioned between 100 mL of ethyl acetate and 100 mL of brine. The organic layer was then washed twice with 50 mL portions of brine, dried over anhydrous sodium sulfate, filtered, concentrated, and purified via silica gel column chromatography. The column was eluted with a gradient of 1-5% methanol in dichloromethane, yielding 3.6 g of compound **16** with a 90% yield.

^{1}H NMR (400 MHz, DMSO)：δ7.37–7.30 (m, 5H), 7.15–7.08 (m, 2H), 6.84–6.79 (m, 2H), 5.01 (s, 2H), 4.03 (q, *J* = 7.1 Hz, 2H), 3.93 (t, *J* = 6.3 Hz, 2H), 3.16 (q, *J* = 6.5 Hz, 2H), 2.77 (t, *J* = 7.5 Hz, 2H), 2.55 (t, *J* = 7.5 Hz, 2H), 1.84 (p, *J* = 6.5 Hz, 2H), 1.15 (t, *J* = 7.1 Hz, 3H).

^{13}C NMR (100 MHz, DMSO)：δ172.70, 157.41, 156.63, 137.71, 132.85, 129.66, 128.82, 128.22, 127.83, 114.75, 65.65, 65.46, 60.24, 38.32, 37.82, 35.91, 29.93, 29.65, 14.57.

MS (ESI, m/z)：calcd for $C_{22}H_{27}NO_5$ $[M-H]^-$:384.19; found:383.98.

3.3.7 Preparation of compound 17

Compound **16** (3.5 g, 9.1 mmol) was dissolved in 3 N sodium hydroxide solution and stirred for 1.5 h. After the reaction was complete, the resulting solution was acidified to pH 3.0-5.0 using 1.0 N hydrochloric acid. The mixture was then partitioned between 100 mL of ethyl acetate and 100 mL of brine, and the organic layer was washed twice with 50 mL portions of brine. The organic phase was dried over anhydrous sodium sulfate, filtered, concentrated, and purified via silica gel column chromatography. The column was eluted with a gradient of 1.0-4.0% methanol in dichloromethane, yielding

2.76 g of compound **17** with an 85% yield.

^{1}H NMR (400 MHz, DMSO)：δ12.10 (s, 1H), 7.38–7.29 (m, 6H), 7.15–7.10 (m, 2H), 6.84–6.78 (m, 2H), 5.02 (s, 2H), 3.93 (t, *J* = 6.3 Hz, 2H), 3.16 (q, *J* = 6.5 Hz, 2H), 2.75 (t, *J* = 7.6 Hz, 2H), 2.51–2.46 (m, 2H), 1.84 (p, *J* = 6.6 Hz, 2H).

^{13}C NMR (100 MHz, DMSO)：δ174.30, 157.34, 156.63, 137.71, 133.22, 129.64, 128.82, 128.22, 127.82, 114.74, 65.66, 65.46, 37.82, 36.06, 29.98, 29.65.

MS (ESI, m/z)：calcd for $C_{20}H_{23}NO_5$ $[M-H]^-$:356.16; found:356.44.

3.3.8 Preparation of compound 18 and 19

In a 250 mL round-bottom flask, Propargyl-PEG8-acid (1.1 g, 2.5 mmol), N-hydroxysuccinimide (NHS, 0.86 g, 7.5 mmol), and EDCI(1.44 g, 7.5 mmol) were combined. Anhydrous dichloromethane (30 mL) was added to the mixture, which was then stirred at room temperature for 3 h. After the reaction was complete, the mixture was partitioned between 100 mL of ethyl acetate and 100 mL of brine. The organic layer was washed twice with 50 mL portions of brine, dried over anhydrous sodium sulfate, filtered, and concentrated.

A 250 mL round-bottom flask was charged with 1.24 g (2.3 mmol) of compound **15**, 1.6 mL of triethylamine, and 30 mL of anhydrous N,N-dimethylformamide (DMF). The mixture was stirred at room temperature for 0.5 h under a nitrogen atmosphere. Compound **18** (1.24 g, 2.3 mmol) was then added, and stirring continued for an additional 3 h at room temperature. After the reaction was complete, the solution was acidified to pH 3.0-5.0 using 1.0 N hydrochloric acid. The mixture was partitioned between 100 mL of ethyl acetate and 100 mL of brine, and the organic layer was washed twice with 50 mL portions of brine. The organic phase was dried over anhydrous sodium sulfate, filtered, concentrated, and purified via silica gel column chromatography. The column was eluted with a gradient of 1-6% methanol in dichloromethane, yielding 2 g of compound **19** with a 70% yield.

^{1}H NMR (400 MHz, DMSO) δ7.90 (t, *J* = 5.7 Hz, 1H), 7.76 (t, *J* = 5.6 Hz, 1H), 7.08 (d, *J* = 8.5 Hz, 2H), 6.83–6.78 (m, 2H), 6.28 (dd, *J* = 14.3, 8.3 Hz, 2H), 4.14 (d, *J* = 2.4 Hz, 2H), 4.04 (m, 1H), 3.93 (m, 3H), 3.59 (t, *J* = 6.5 Hz, 2H), 3.55–3.45 (m, 28H), 3.42 (t, *J* = 2.4 Hz, 1H), 3.18 (q, *J* = 6.5 Hz, 2H), 3.00 (q, *J* = 6.4 Hz, 2H), 2.75–2.69 (m, 2H), 2.34–2.16 (m, 6H), 1.84 (m, *J* = 19.7, 6.2 Hz, 3H), 1.70–1.53 (m, 2H), 1.39 (d, *J* = 3.0 Hz, 27H), 1.24 (s, 3H).

^{13}C NMR (100 MHz, DMSO)：δ 172.74, 172.36, 171.89, 171.60, 170.51, 157.57, 157.25, 133.72, 129.55, 114.70, 81.06, 80.79, 80.75, 80.21, 77.57, 70.24, 70.14, 70.00,

69.97, 68.97, 67.32, 65.55, 57.95, 55.39, 53.48, 52.61, 38.67, 37.92, 36.64, 35.94, 32.21, 31.34, 30.79, 29.40, 29.27, 28.19, 28.10, 22.96.

MS (ESI, m/z): calcd for $C_{56}H_{94}N_4O_{18}$ $[M+Na]^+$:1133.65; found:1133.78.

3.3.9 Preparation of EuK-linker B (20)

A 100 mL round-bottom flask was loaded with 1.5 g of compound **19**, followed by the addition of 3 mL of dichloromethane (DCM) and 3 mL of trifluoroacetic acid (TFA). The mixture was stirred at ambient temperature overnight. After the reaction concluded, the solution was evaporated to dryness and subjected to purification via reversed-phase column chromatography. The column was eluted with a gradient of 20% to 65% water in acetonitrile, yielding 1.1 g of **EuK-linker B** (20) with a 75% yield.

^{1}H NMR (400 MHz, DMSO): δ7.33–7.28 (m, 1H), 7.11 (d, *J* = 8.5 Hz, 2H), 6.90–6.74 (m, 2H), 4.14 (d, *J* = 2.4 Hz, 2H), 3.99 (s, 28H), 3.29 (t, *J* = 2.4 Hz, 1H), 3.24–3.19 (m, 2H), 3.01 (t, *J* = 6.9 Hz, 2H), 2.74 (t, *J* = 7.5 Hz, 2H), 2.31 (m, 6H), 2.01–1.70 (m, 5H), 1.68–1.48 (m, 2H), 1.35 (q, *J* = 6.5 Hz, 2H), 1.25 (p, *J* = 6.3 Hz, 3H).

^{13}C NMR (101 MHz, DMSO): δ 175.03, 174.65, 174.20, 171.68, 170.57, 157.77, 157.25, 133.73, 129.56, 114.69, 80.79, 77.53, 70.23, 70.13, 69.99, 69.96, 68.97, 67.31, 65.54, 57.94, 52.72, 52.11, 38.76, 37.88, 36.62, 35.95, 32.24, 30.79, 30.35, 29.38, 29.29, 27.98, 23.07.

MS (ESI, m/z): calcd for $C_{44}H_{70}N_4O_{18}$ $[M+H]^+$:943.46; found:943.58.

3.4 Synthetic scheme 4: Preparation of compound CTX-linker C (23)

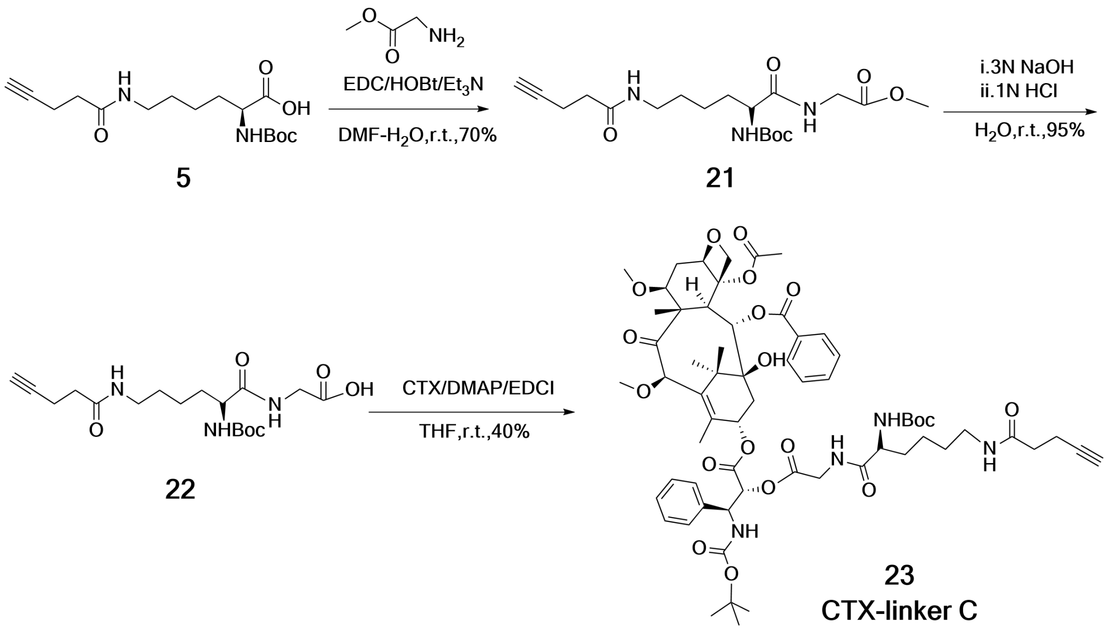

Scheme S4. Synthetic route of CTX-linker C

3.4.1 Preparation of compound 21

Glycine methyl ester hydrochloride (1.14 g, 9.3 mmol) and triethylamine (1.73 mL, 12.4 mmol) were dissolved in 10 mL of anhydrous N,N-dimethylformamide (DMF), heated at 35°C for 1 h, and then cooled to ambient temperature. Subsequently, compound **5** (1 g, 3.1 mmol), EDCI (1.2 g, 6.2 mmol), and 1-hydroxybenzotriazole (HOBt, 0.84 g, 6.2 mmol) were added, and the mixture was stirred at room temperature overnight. After the reaction was completed, the mixture was partitioned between 100 mL of ethyl acetate and 100 mL of brine. The organic layer was washed twice with 50 mL portions of brine, dried over anhydrous sodium sulfate, filtered, concentrated, and purified via silica gel column chromatography. The column was eluted with a gradient of 2-8% methanol in dichloromethane, yielding 0.86 g of compound **21** with a 70% yield.

^{1}H NMR (400 MHz, DMSO)：δ8.22 (t, J = 5.9 Hz, 1H), 7.85 (t, J = 5.6 Hz, 1H), 6.83 (d, J = 8.2 Hz, 1H), 3.95–3.75 (m, 3H), 3.62 (s, 3H), 3.09–2.95 (m, 2H), 2.74 (t, J = 2.6 Hz, 1H), 2.35 (m, 2H), 2.25 (dd, J = 7.8, 6.2 Hz, 2H), 1.65–1.44 (m, 2H), 1.38 (s, 11H), 1.31 (s, 2H).

^{13}C NMR (100 MHz, DMSO)：δ173.26, 170.74, 170.49, 155.78, 84.23, 78.46, 71.72, 54.48, 52.12, 40.97, 38.79, 34.72, 32.06, 29.28, 28.66, 28.44, 23.25, 14.77.

MS (ESI, m/z)：calcd for $C_{19}H_{31}N_3O_6$ $[M+H]^+$:398.22; found:398.21.

3.4.2 Preparation of compound 22

Compound **21** (0.86 g, 2.17 mmol) was dissolved in 8 mL of methanol, and 3 N sodium hydroxide (NaOH) solution was added subsequently. The mixture was stirred at room temperature for 1 h. After the reaction was completed, the solution was adjusted to a pH range of 3.0-5.0 using 1.0 N hydrochloric acid (HCl). The reaction mixture was then partitioned between 100 mL of ethyl acetate and 100 mL of brine. The organic layer was washed twice with 50 mL portions of brine, dried over anhydrous sodium sulfate, filtered, concentrated, and purified via silica gel column chromatography. The column was eluted with a gradient of 2-20% methanol in dichloromethane, yielding 0.8 g of compound **22** with a 95% yield.

^{1}H NMR (400 MHz, DMSO)：δ8.06 (t, J = 5.8 Hz, 1H), 7.85 (t, J = 5.6 Hz, 1H), 6.82 (d, J = 8.3 Hz, 1H), 3.91 (m, 1H), 3.83–3.65 (m, 2H), 3.01 (m, 2H), 2.75 (t, J = 2.6 Hz, 1H), 2.37–2.32 (m, 2H), 2.24 (dd, J = 7.7, 6.2 Hz, 2H), 1.65–1.43 (m, 2H), 1.38 (s, 11H), 1.30–1.21 (m, 2H).

^{13}C NMR (100 MHz, DMSO)：δ173.03, 171.66, 170.49, 155.79, 128.26, 127.47, 124.82, 119.52, 110.19, 84.24, 78.46, 71.74, 54.51, 41.07, 38.80, 34.71, 32.12, 29.28, 28.67, 28.44, 23.30, 14.77.

MS (ESI, m/z): calcd for $C_{18}H_{29}N_3O_6$ $[M-H]^-$:382.21; found:382.06.

3.4.3 Preparation of CTX-linker C (23)

The title compound was prepared using the method similar to that described for **CTX-GLA-linker A (10)**.

^{1}H NMR (400 MHz, DMSO): δ8.24 (m, 1H), 7.97 (dd, *J* = 8.3, 6.7 Hz, 2H), 7.93–7.83 (m, 2H), 7.77–7.71 (m, 1H), 7.66 (dd, *J* = 8.2, 6.7 Hz, 2H), 7.47–7.27 (m, 4H), 7.19 (t, *J* = 7.4 Hz, 1H), 6.86 (d, *J* = 8.5 Hz, 1H), 5.84 (t, *J* = 9.1 Hz, 1H), 5.37 (d, *J* = 7.0 Hz, 1H), 5.13–5.06 (m, 2H), 4.97–4.92 (m, 1H), 4.68 (s, 1H), 4.51 (s, 1H), 4.05–4.01 (m, 2H), 3.62 (s, 1H), 3.57 (s, 1H), 3.27 (s, 3H), 3.20 (s, 3H), 3.01 (q, *J* = 6.5 Hz, 2H), 2.89 (s, 1H), 2.79–2.56 (m, 4H), 2.34 (m, 3H), 2.24 (d, *J* = 7.2 Hz, 4H), 1.77 (s, 2H), 1.50 (s, 4H), 1.38 (d, *J* = 3.4 Hz, 21H), 1.29–1.24 (m, 2H), 0.97 (d, *J* = 8.1 Hz, 6H).

^{13}C NMR (100 MHz, DMSO): δ205.10, 173.25, 170.75, 170.48, 170.15, 169.74, 169.29, 165.71, 162.78, 155.86, 155.78, 155.69, 138.96, 137.80, 135.37, 133.96, 130.37, 130.06, 129.17, 129.10, 128.58, 127.85, 84.24, 84.21, 83.71, 82.47, 80.76, 80.65, 79.08, 78.49, 77.18, 75.70, 74.79, 71.73, 71.53, 60.23, 57.09, 56.96, 56.44, 55.58, 54.48, 52.12, 46.85, 43.39, 40.98, 38.79, 38.74, 36.26, 34.88, 34.71, 32.07, 31.98, 31.24, 29.33, 29.29, 28.67, 28.59, 27.09, 23.32, 23.01, 21.59, 21.24, 14.77, 14.56, 10.60.

MS (ESI, m/z): calcd for $C_{63}H_{84}N_4O_{19}$ $[M+H]^+$:1201.57; found:1201.12.

3.5 Synthetic scheme 5: Preparation of Dextran-CTX-GLA-EuK (24)

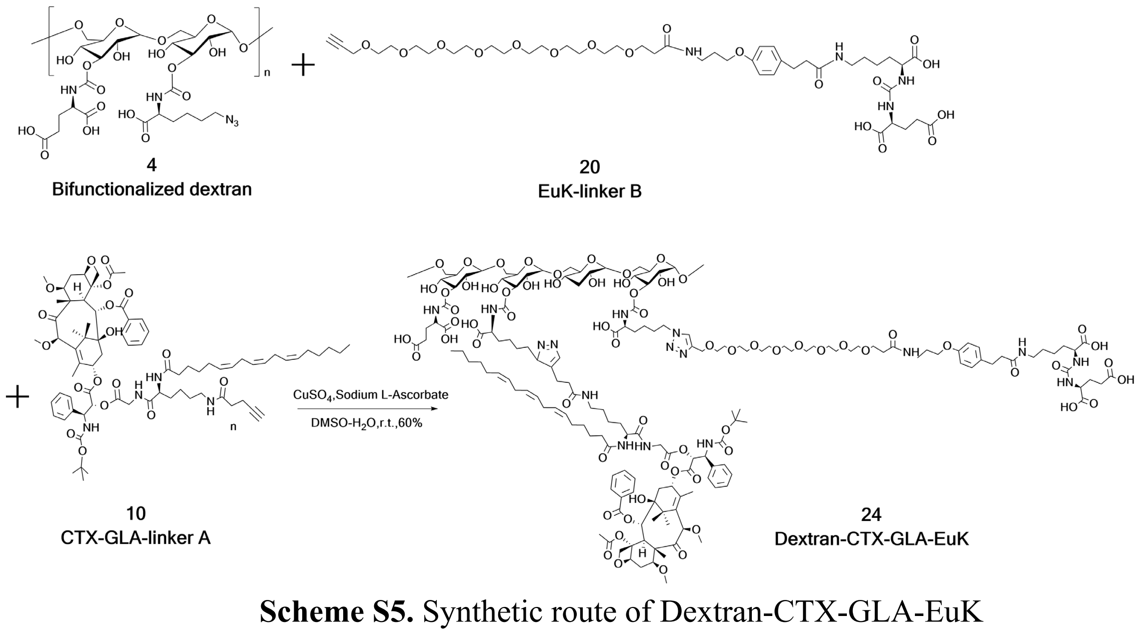

Scheme S5. Synthetic route of Dextran-CTX-GLA-EuK

Bifunctionalized dextran (4) (254 mg), **EuK-linker B (20)** (0.024 mmol), and

CTX-GLA-linker A (10) (0.024 mmol) were mixed in a 9:1 DMSO-water co-solvent system. Subsequently, 100 μL of 1.0 N copper sulfate ($CuSO_4$, 0.1 mmol) and 200 μL of 1.0 M sodium ascorbate (0.2 mmol) were added, and the mixture was stirred at room temperature overnight. After the reaction was completed, the mixture was diluted with 50 mL of distilled water and filtered. The resulting filtrate was washed with 50 mL of dichloromethane, dialyzed against distilled water three times, concentrated, and purified via Sephadex G-100 column chromatography. The column was eluted with a gradient of 5-10% methanol in distilled water. The target product fractions were collected, concentrated, and lyophilized to yield 223 mg of the dual conjugate **Dextran-CTX-GLA-EuK (24)**, with a 56% yield. The conjugates **Dextran-CTX-GLA**, **Dextran-CTX-EuK and Dextran-CTX** were obtained by the same synthesis and treatment methods.

3.6 Synthetic scheme 6: Preparation of Dextran-Cy7.5 and Dextran-Cy7.5-EuK

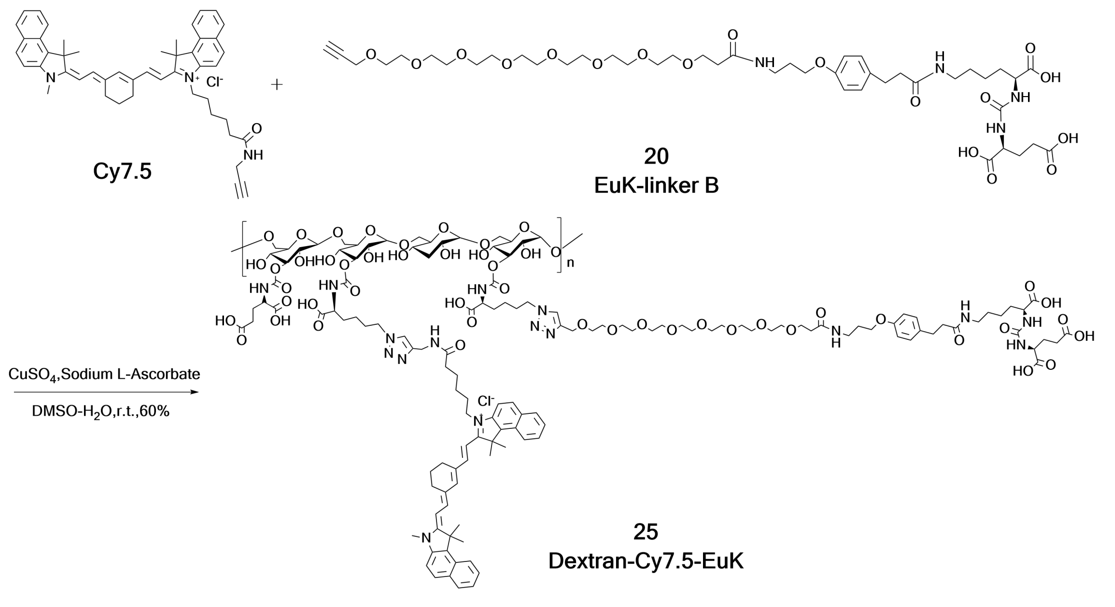

Scheme S6. Synthetic route of Dextran-Cy7.5-EuK

Bifunctionalized dextran (4) (250 mg), **EuK-linker B (20)** (0.024 mmol), and compound **Cy7.5** (10 mg) were combined in a 9:1 dimethyl sulfoxide (DMSO)-water mixture. Next, 100 μL of 1.0 N copper sulfate ($CuSO_4$, 0.1 mmol) and 200μL of 1.0 M sodium ascorbate (0.2 mmol) were added sequentially, and the reaction mixture was stirred at ambient temperature overnight. Upon reaction completion, the mixture was diluted with 50 mL of distilled water and filtered. The resulting filtrate was rinsed with 50 mL of dichloromethane, dialyzed against distilled water three times, concentrated, and purified using a Sephadex G-100 column. Elution was performed with a 5-10% methanol-in-distilled water gradient. Target product fractions were collected,

concentrated, and freeze-dried to yield 200 mg of the dual conjugate **Dextran-Cy7.5-EuK (25)**. The conjugate **Dextran-Cy7.5** was obtained by the same synthesis and treatment methods.

4 ^{1}H NMR, ^{13}C NMR and HRMS spectra of Compounds

^{1}H NMR、^{13}C NMR and MS spectra of compound **1**

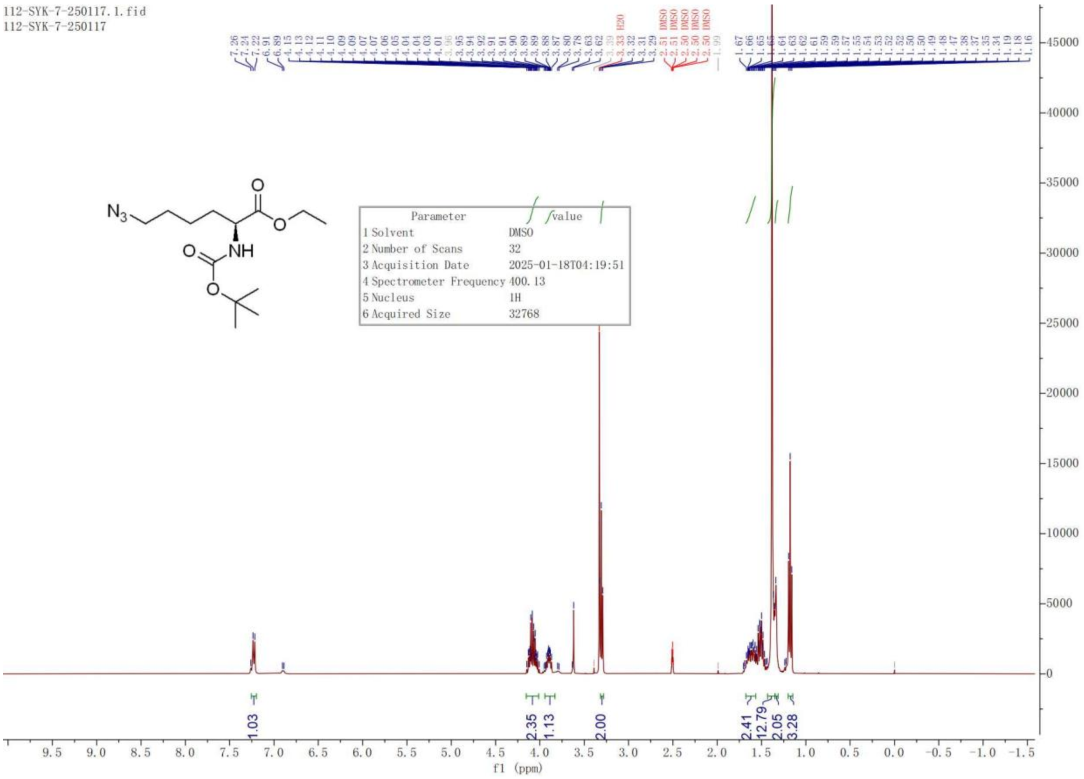

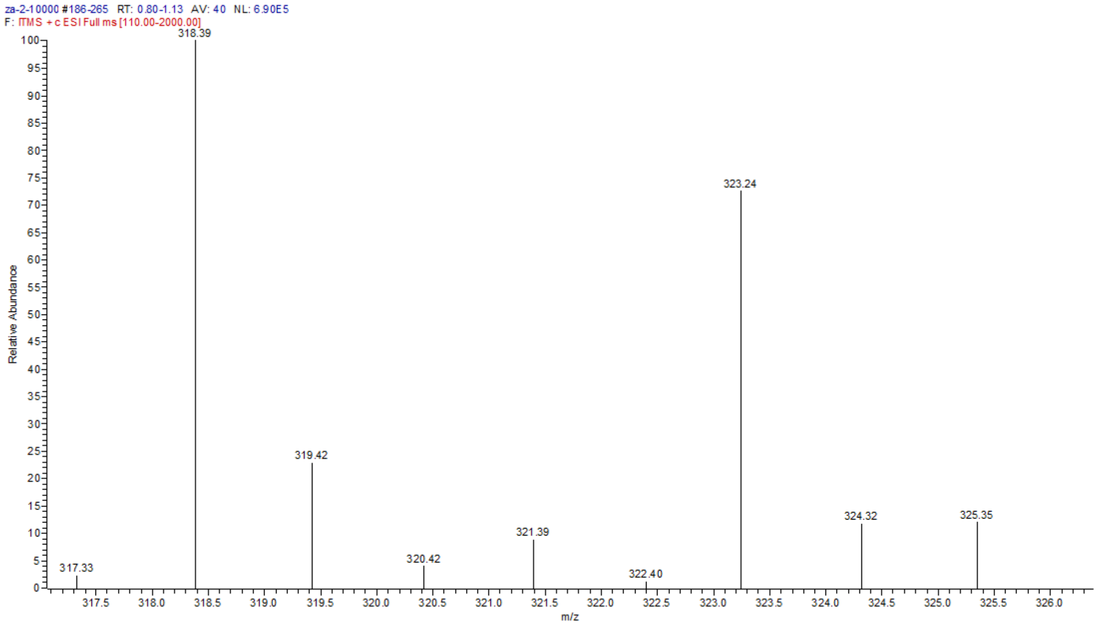

^{1}H NMR、^{13}C NMR and MS spectra of compound **2**

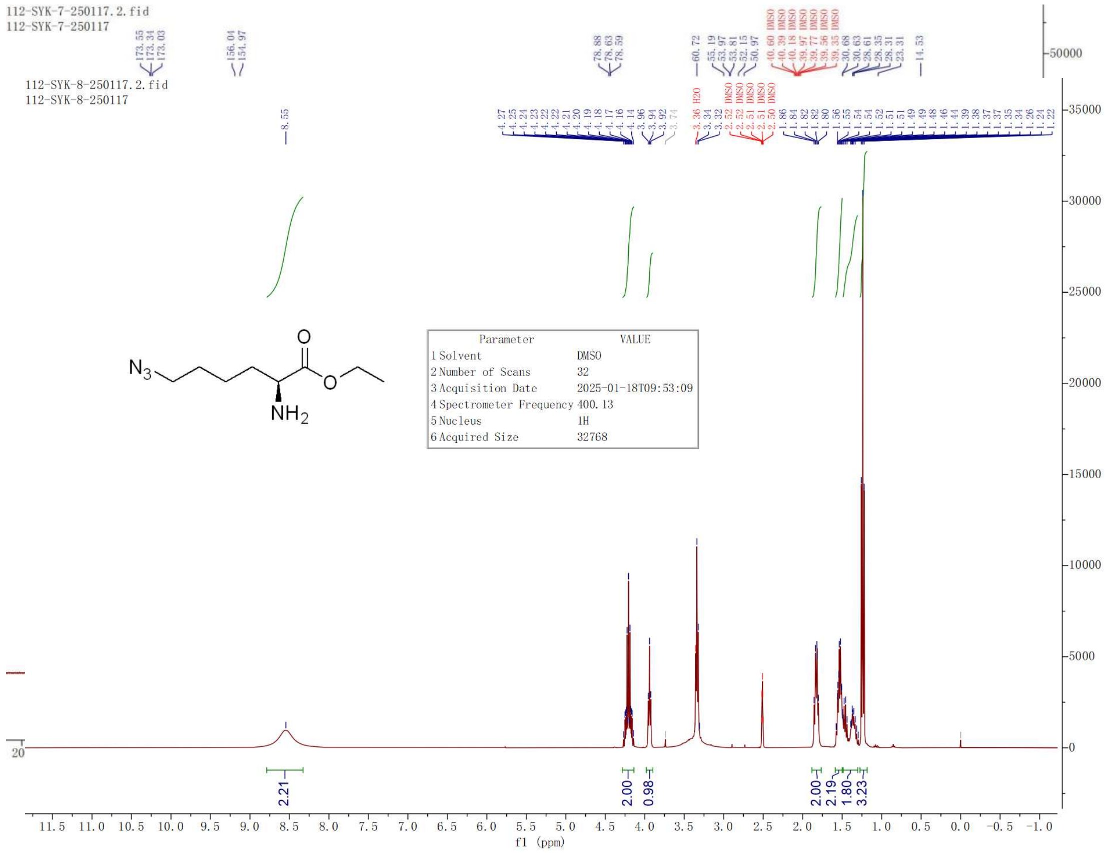

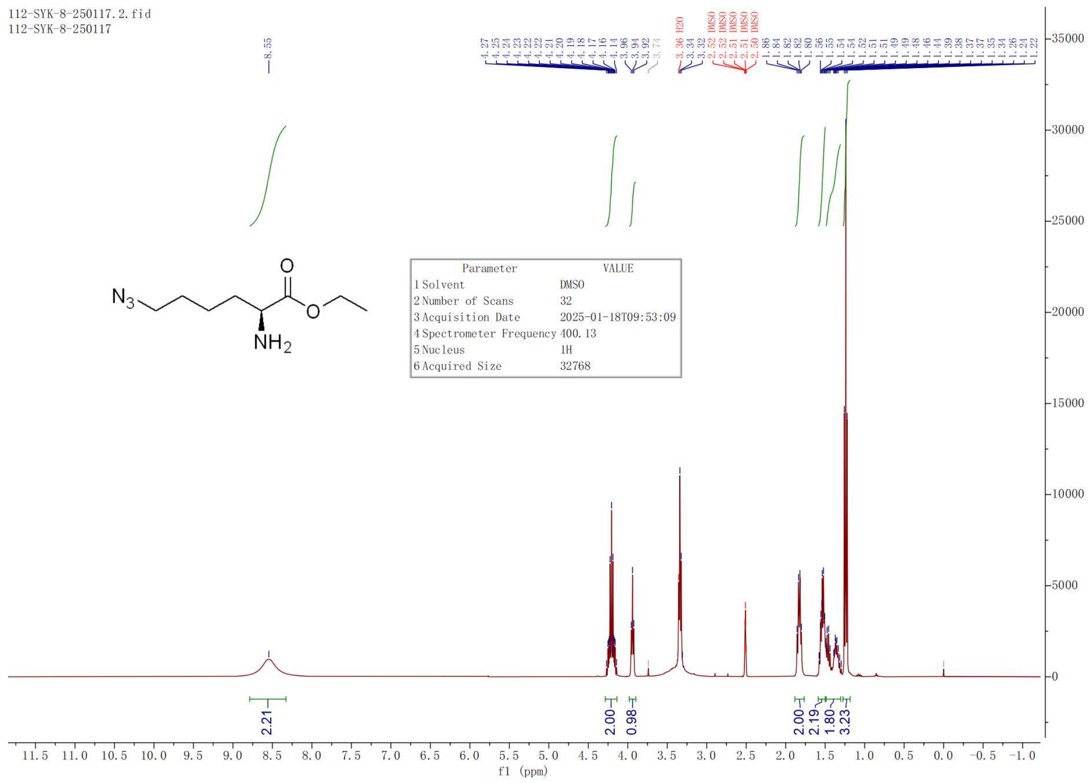
112-SYK-8-250117.2.fid
112-SYK-8-250117
Parameter VALUE
1 Solvent DMSO
2 Number of Scans 32
3 Acquisition Date 2025-01-18T09:53:09
4 Spectrometer Frequency 400.13
5 Nucleus 1H
6 Acquired Size 32768
f1 (ppm)

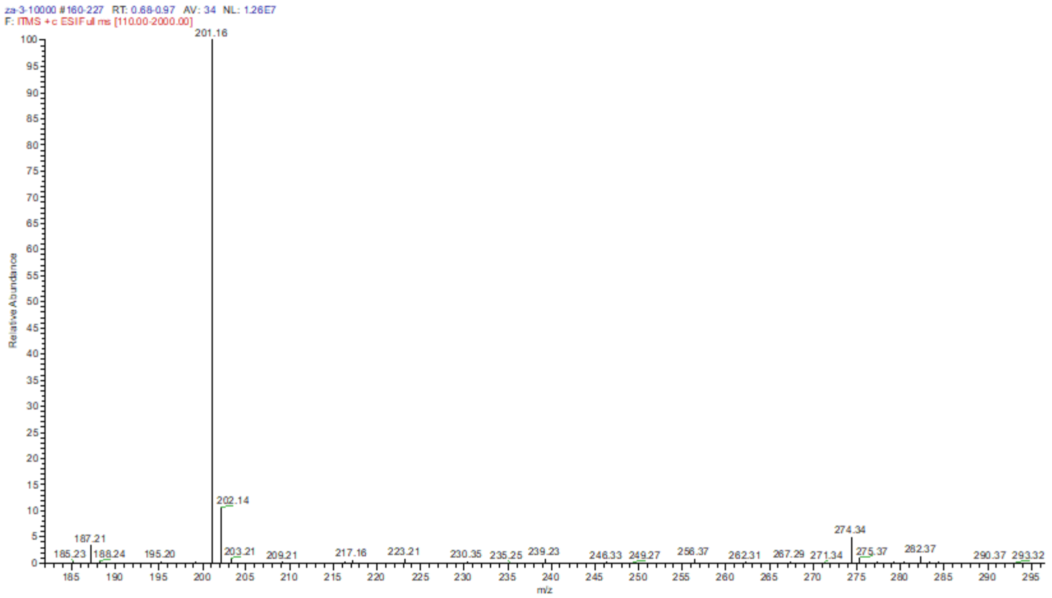
za-3-10000 #160-227 RT: 0.68-0.97 AV: 34 NL: 1.26E7
F: ITMS + c ESI Full ms [110.00-2000.00]
201.16
202.14
274.34
Relative Abundance
m/z

^{1}H NMR of compound **4**

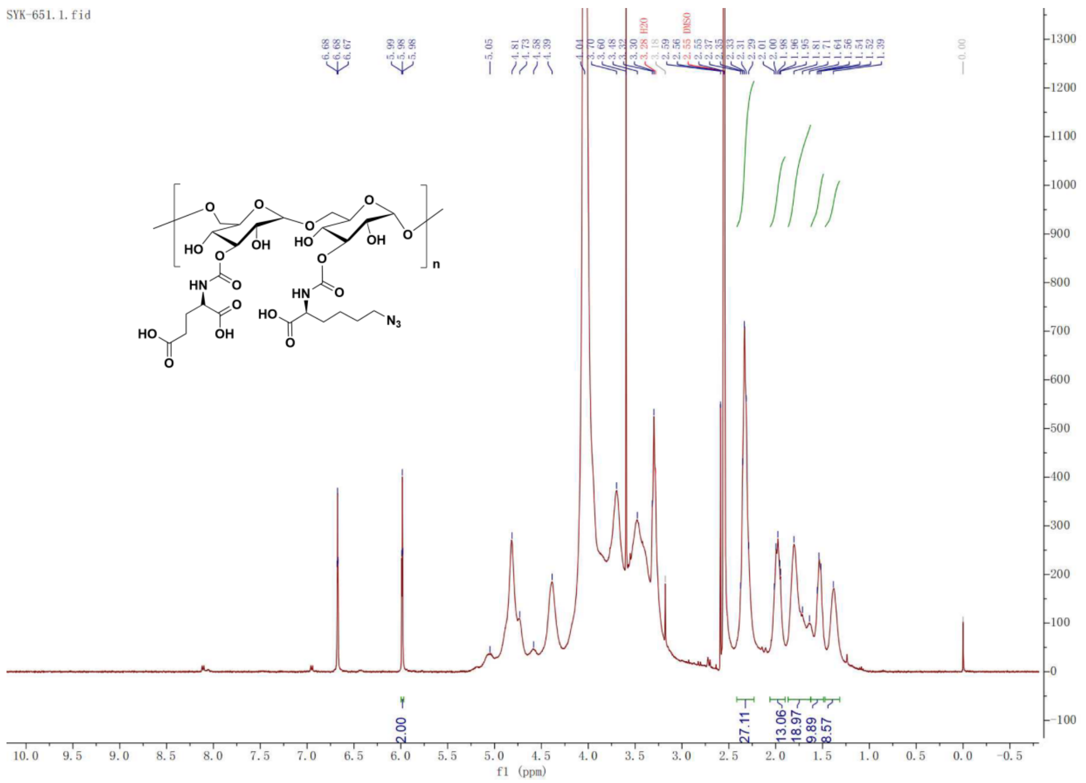

^{1}H NMR、^{13}C NMR and MS spectra of compound **5**

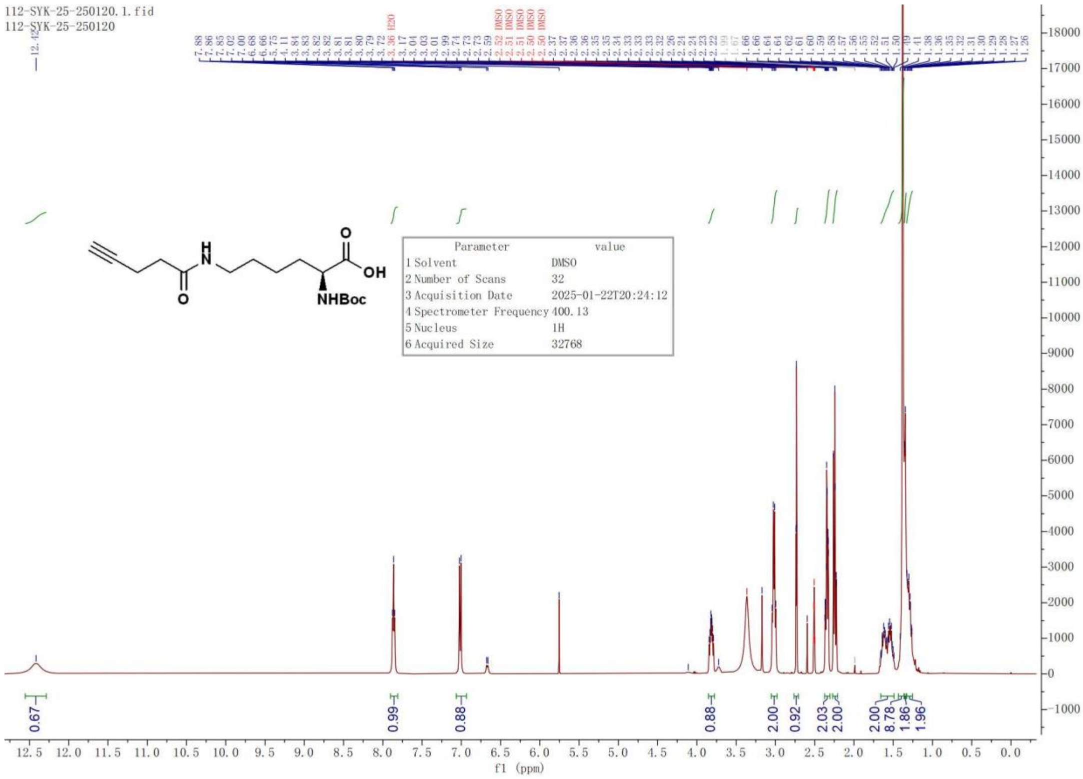

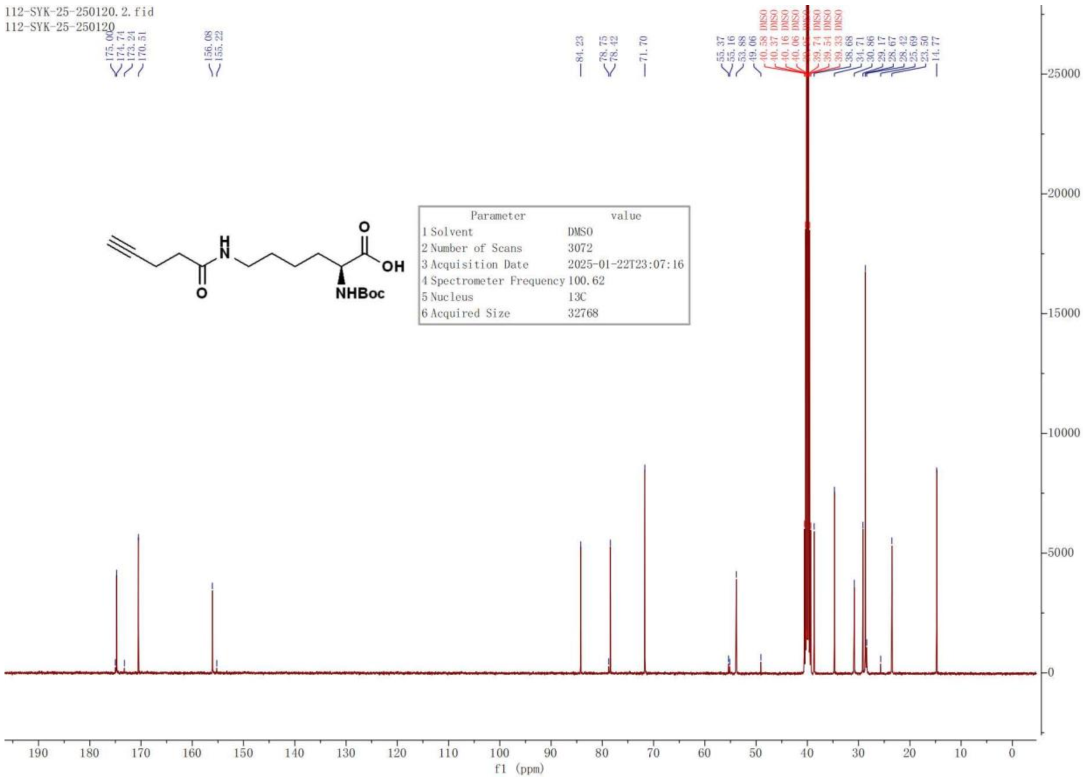

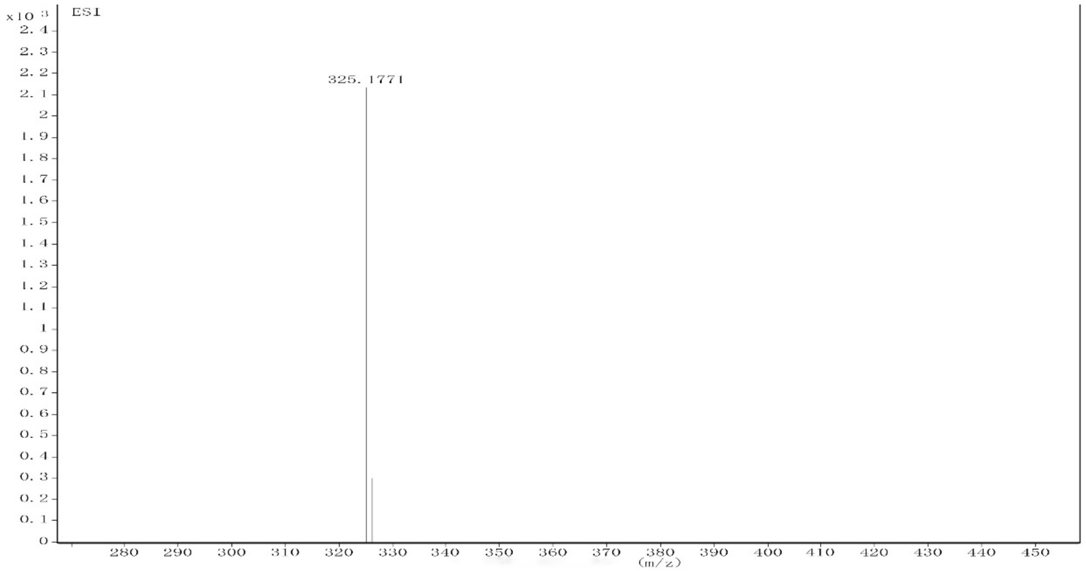

^{1}H NMR、^{13}C NMR and MS spectra of compound **6**

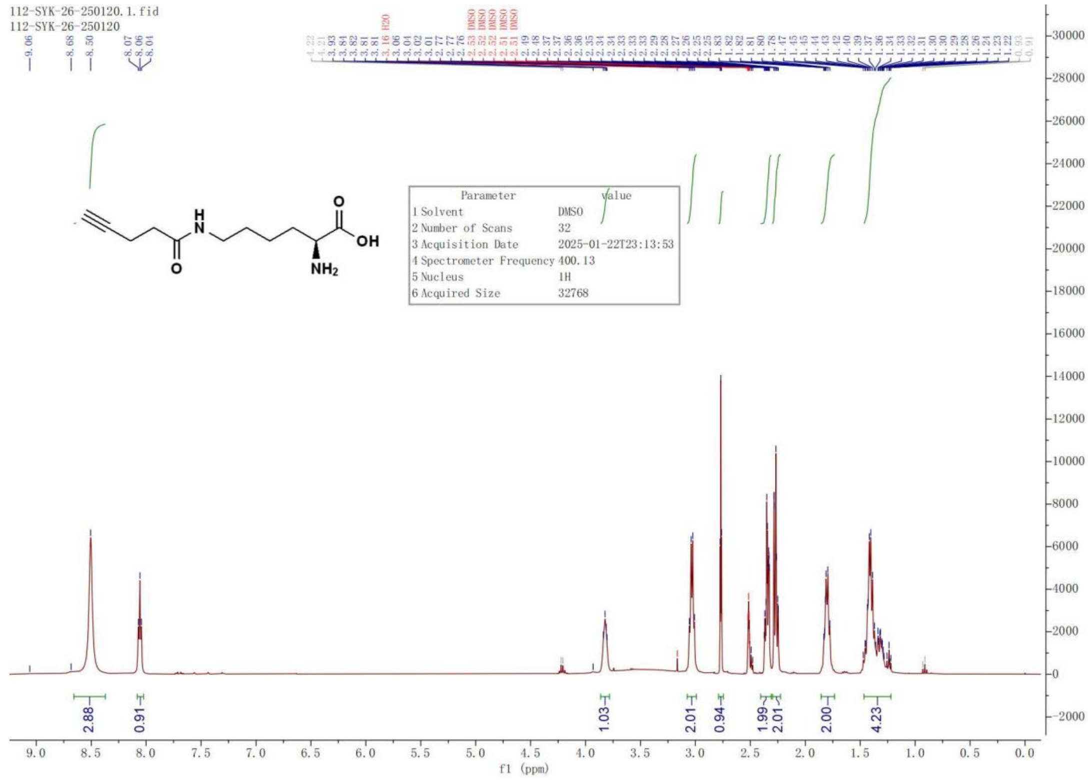

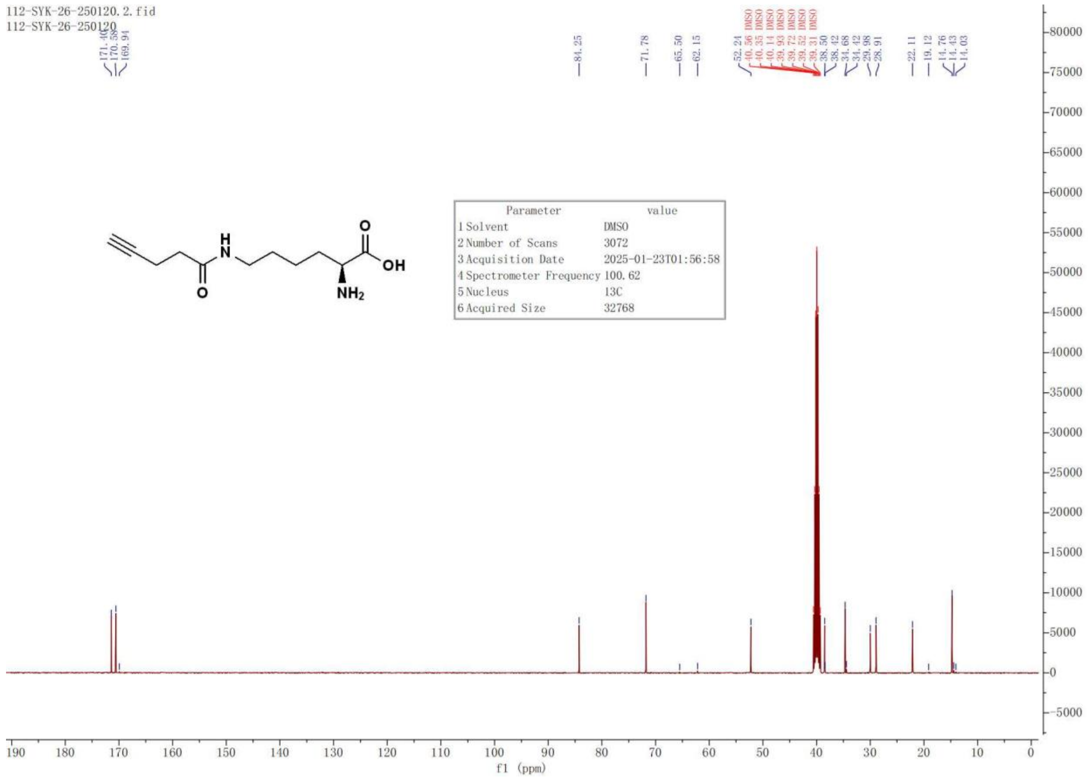
112-SYK-26-250120.2.fid
112-SYK-26-250120
171.46
170.59
169.94
84.25
71.78
65.50
62.15
52.24
40.56 DMSO
40.35 DMSO
40.14 DMSO
39.93 DMSO
39.72 DMSO
39.52 DMSO
39.31 DMSO
38.50
38.42
34.68
34.42
29.98
28.91
22.11
19.12
14.76
14.43
14.03
Parameter value
1 Solvent DMSO
2 Number of Scans 3072
3 Acquisition Date 2025-01-23T01:56:58
4 Spectrometer Frequency 100.62
5 Nucleus 13C
6 Acquired Size 32768
H
N
O
OH
O
NH2
f1 (ppm)

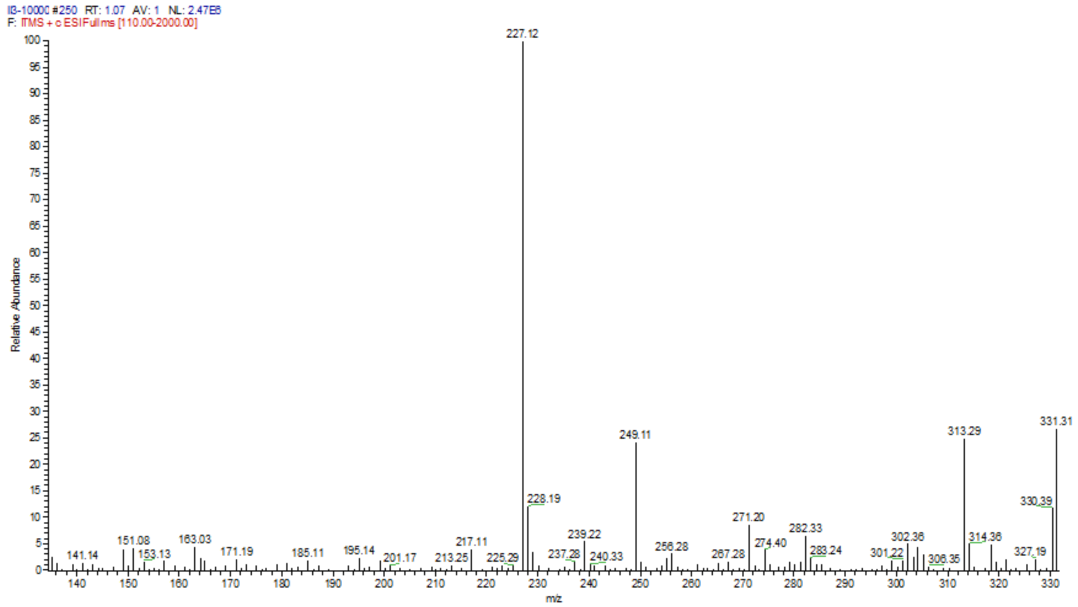
II8-10000 #250 RT: 1.07 AV: 1 NL: 2.47E6
F: ITMS + c ESI Full ms [110.00-2000.00]
Relative Abundance
227.12
249.11
313.29
331.31
330.39
228.19
271.20
282.33
239.22
302.36
314.36
217.11
163.03
151.08
274.40
256.28
283.24
237.28
240.33
195.14
185.11
171.19
153.13
141.14
213.25
225.29
201.17
267.28
301.22
306.35
327.19
m/z

^{1}H NMR、^{13}C NMR and MS spectra of compound **7**

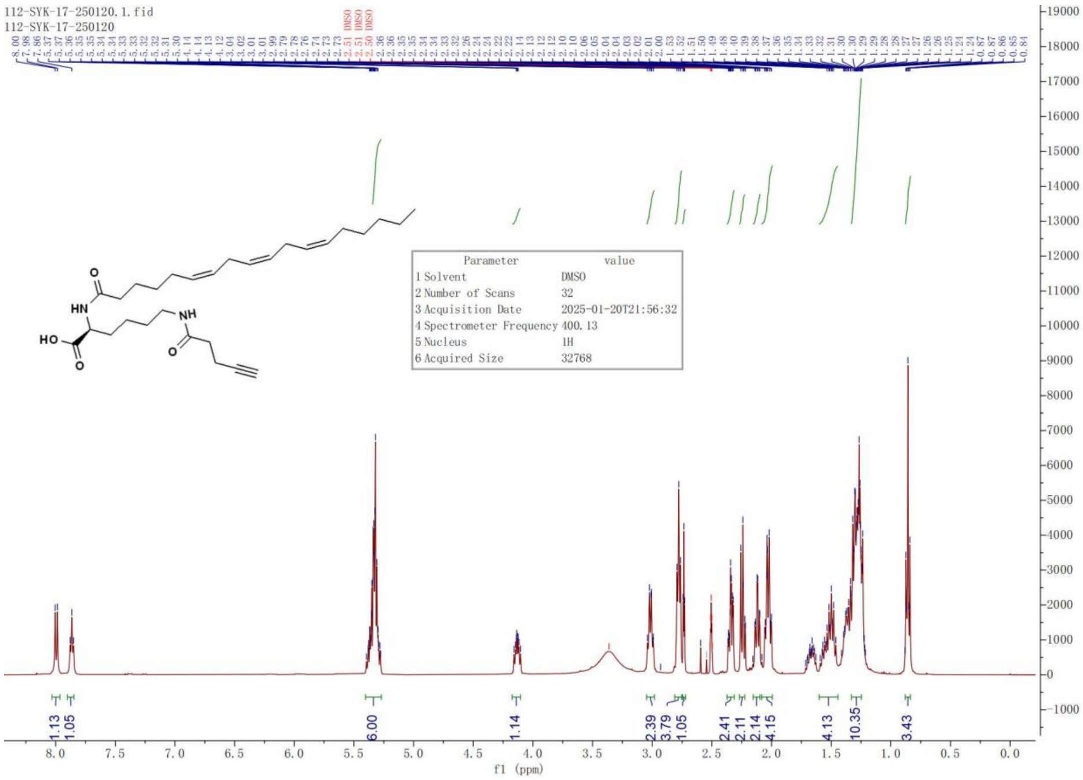

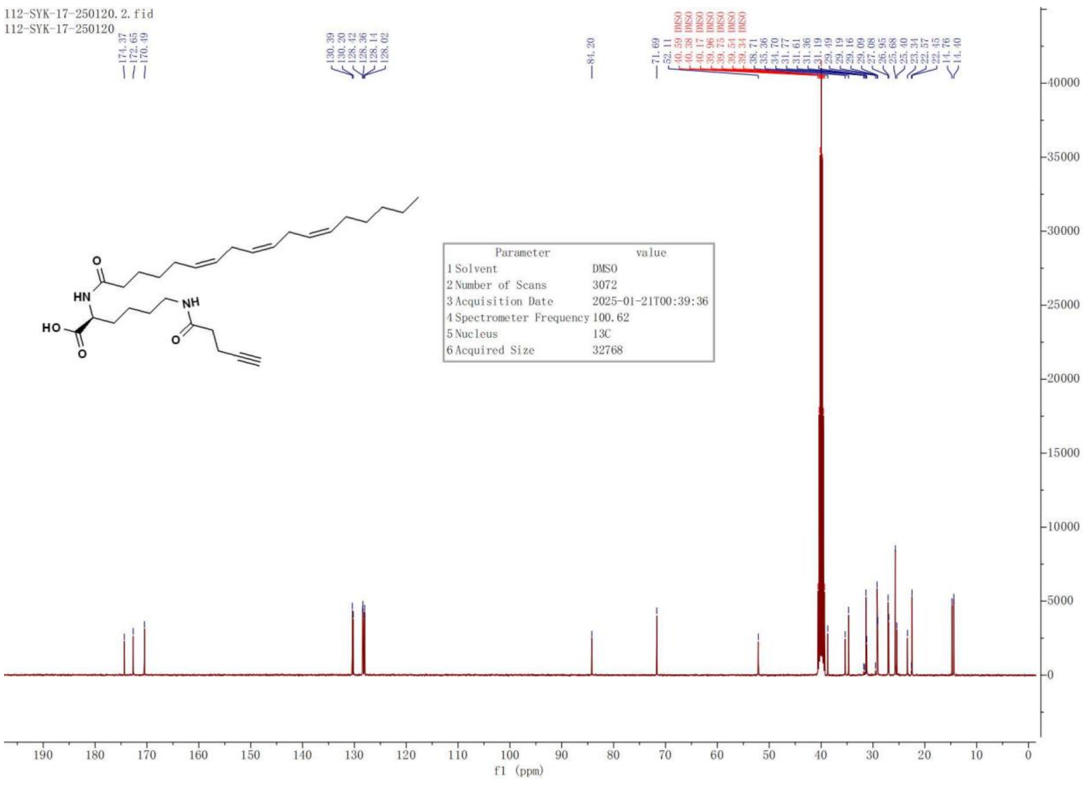

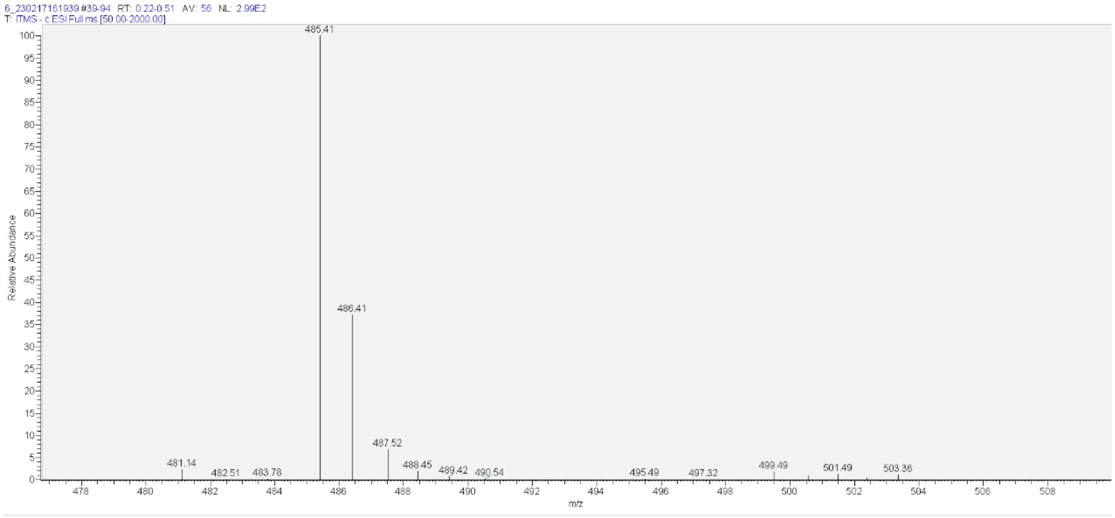

^{1}H NMR、^{13}C NMR and MS spectra of compound **8**

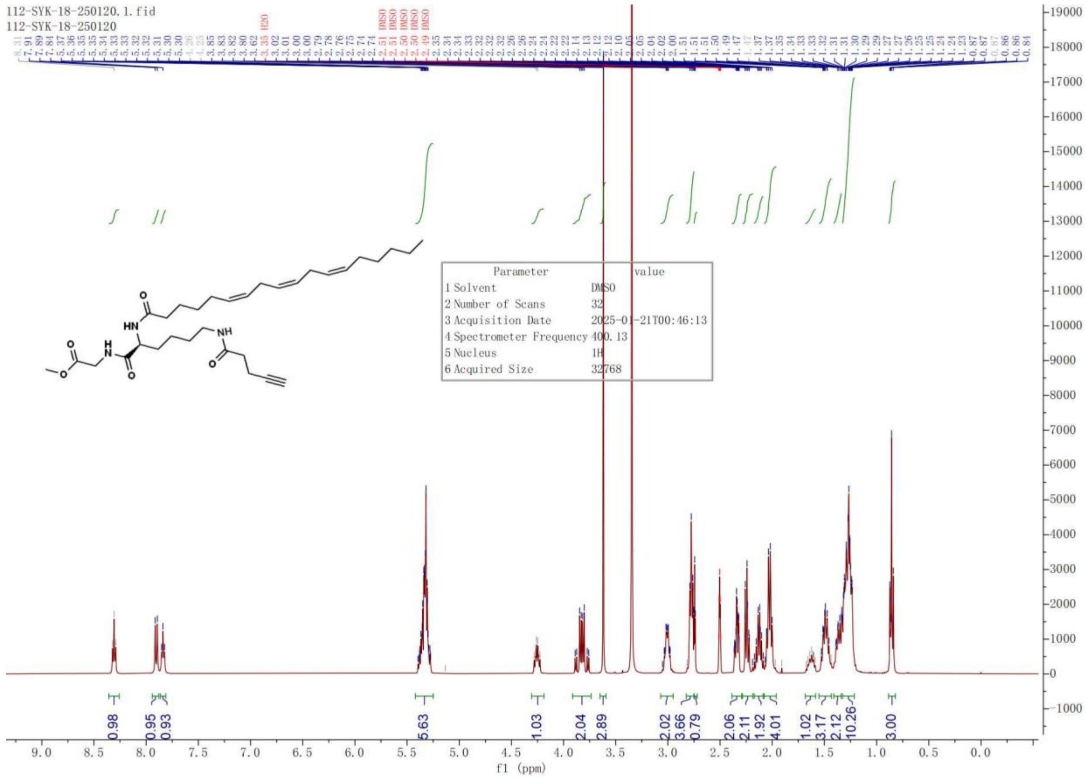

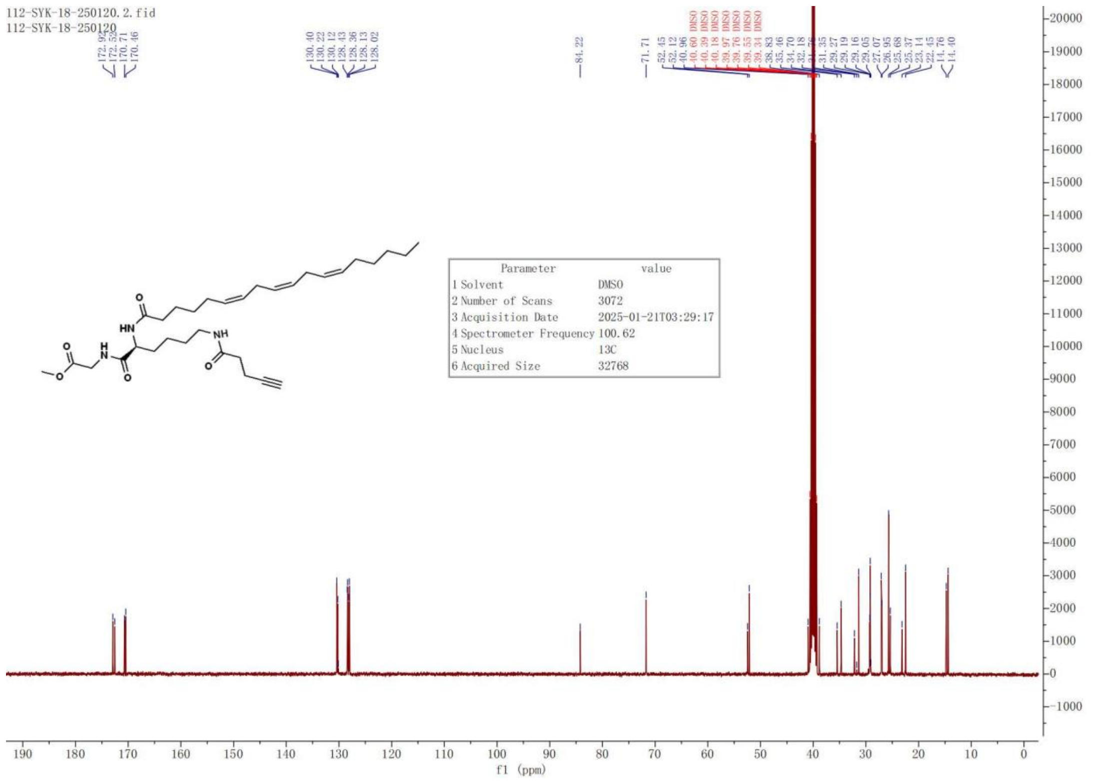

112-SYK-18-250120.2.fid
112-SYK-18-250120
172.92
172.53
170.71
170.46
130.40
130.22
130.12
128.43
128.36
128.13
128.02
84.22
71.71
52.45
52.12
Parameter value
1 Solvent DMSO
2 Number of Scans 3072
3 Acquisition Date 2025-01-21T03:29:17
4 Spectrometer Frequency 100.62
5 Nucleus 13C
6 Acquired Size 32768
f1 (ppm)

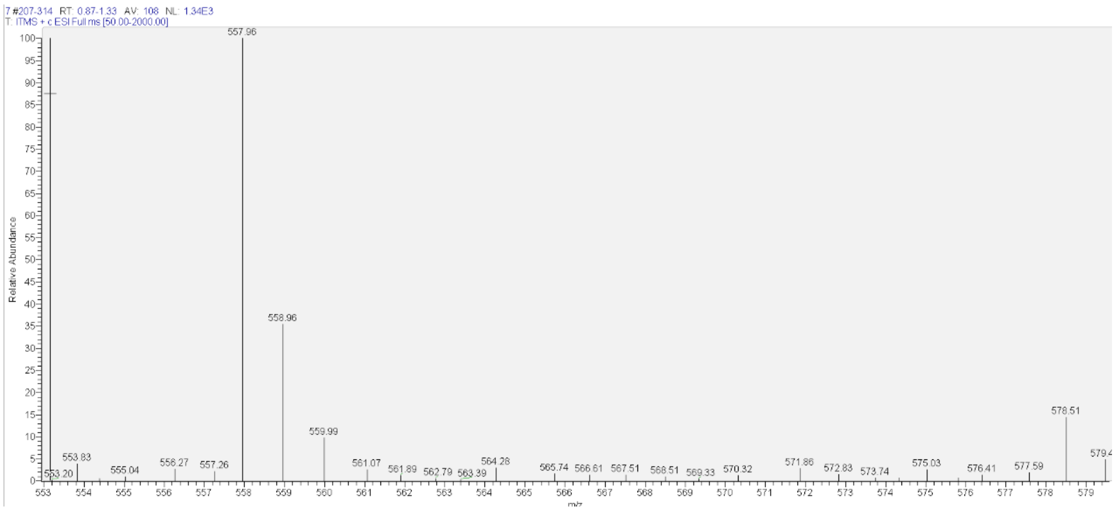

7 #207-314 RT: 0.87-1.33 AV: 108 NL: 1.34E3
T: ITMS + c ESI Full ms [50.00-2000.00]
Relative Abundance
557.96
558.96
559.99
578.51
553.20
553.83
555.04
556.27
557.26
561.07
561.89
562.79
563.39
564.28
565.74
566.61
567.51
568.51
569.33
570.32
571.86
572.83
573.74
575.03
576.41
577.59
m/z

^{1}H NMR、^{13}C NMR and MS spectra of compound **9**

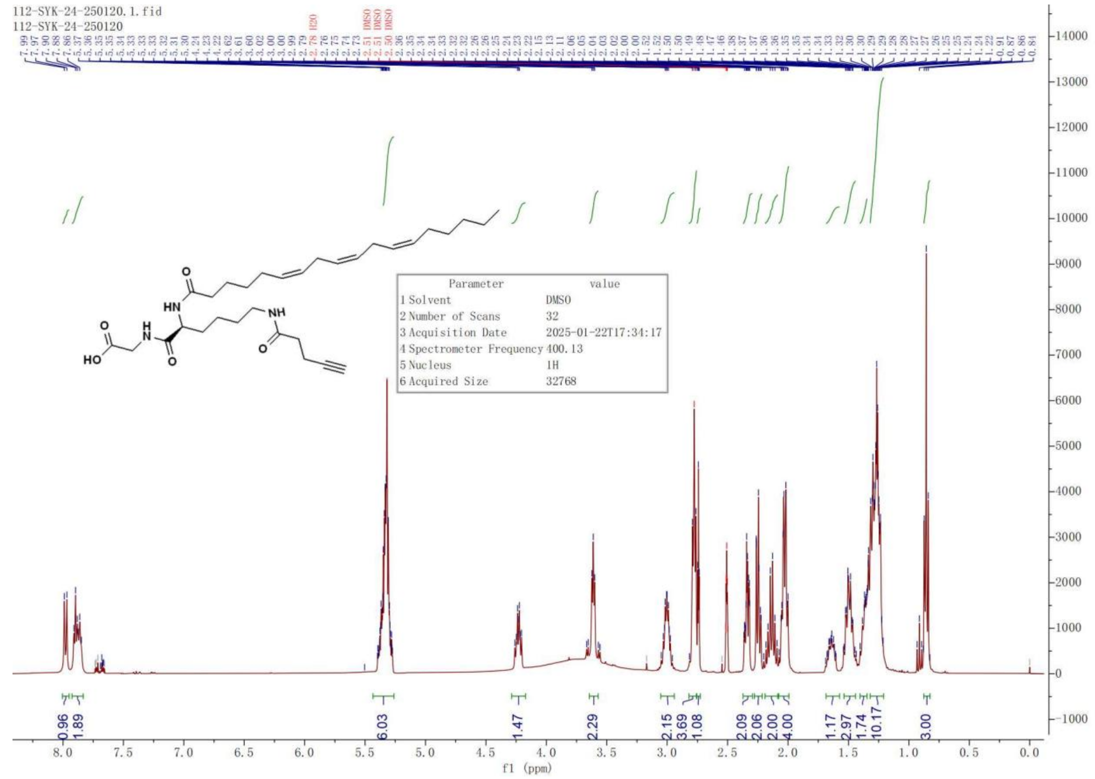

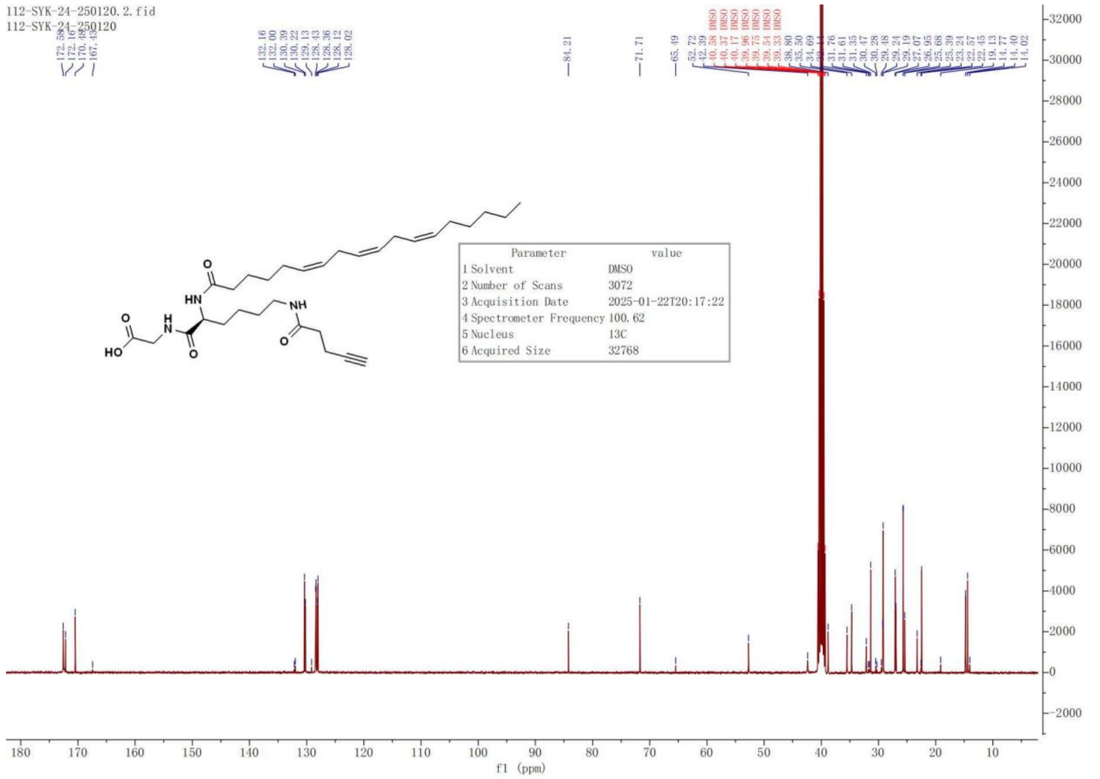

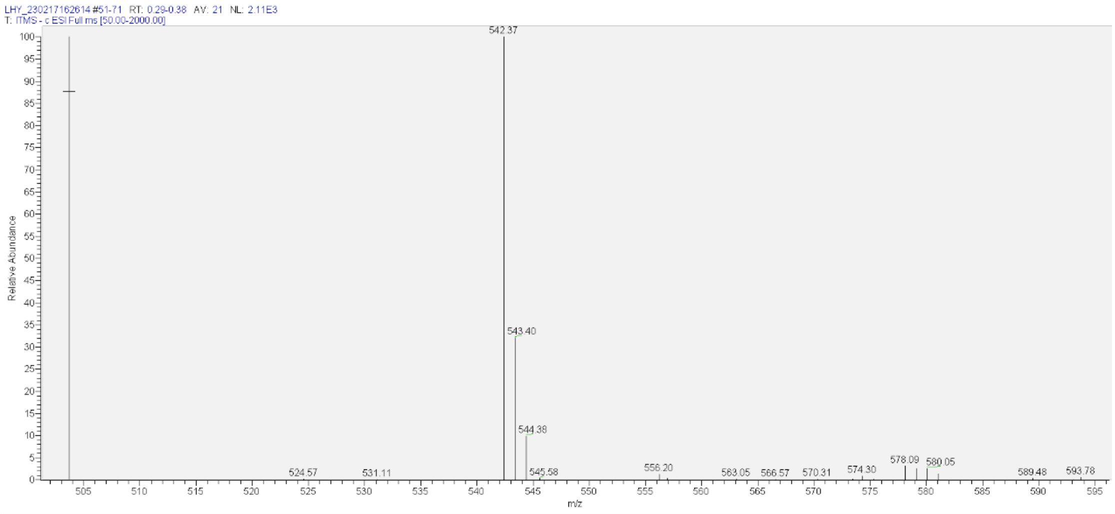

^{1}H NMR、^{13}C NMR and MS spectra of **CTX-GLA-linker A (10)**

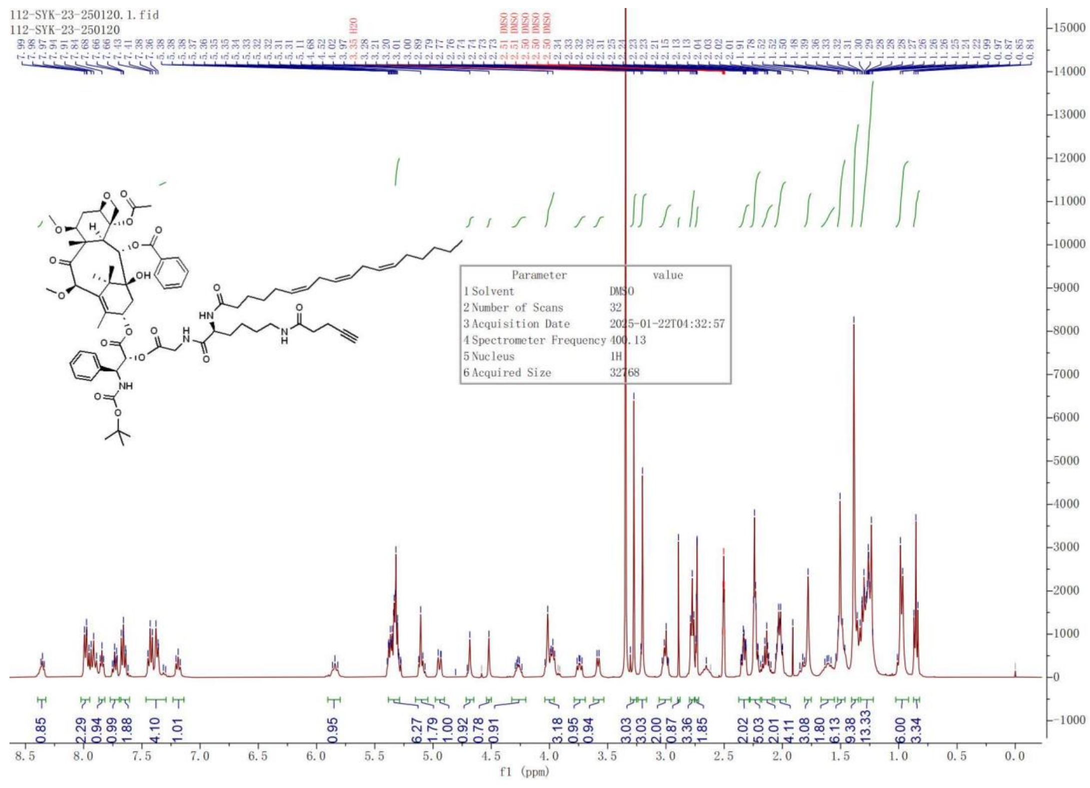

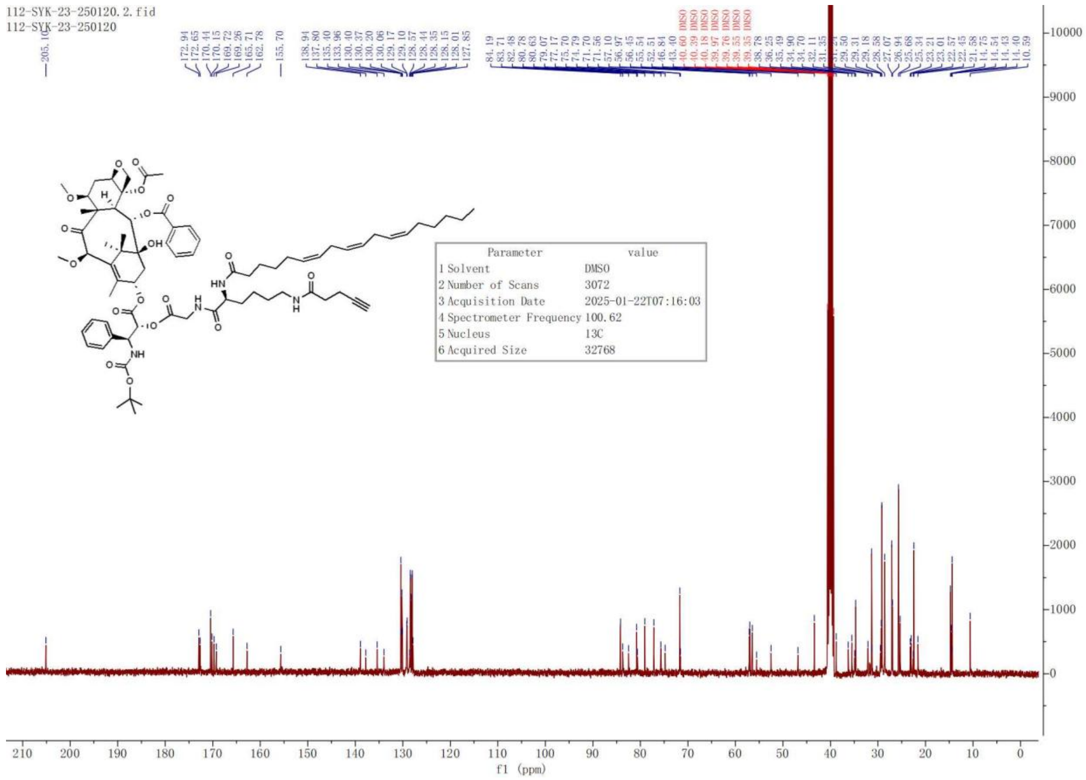
112-SYK-23-250120. 2. fid
112-SYK-23-250120
Parameter value
1 Solvent DMSO
2 Number of Scans 3072
3 Acquisition Date 2025-01-22T07:16:03
4 Spectrometer Frequency 100.62
5 Nucleus 13C
6 Acquired Size 32768
f1 (ppm)

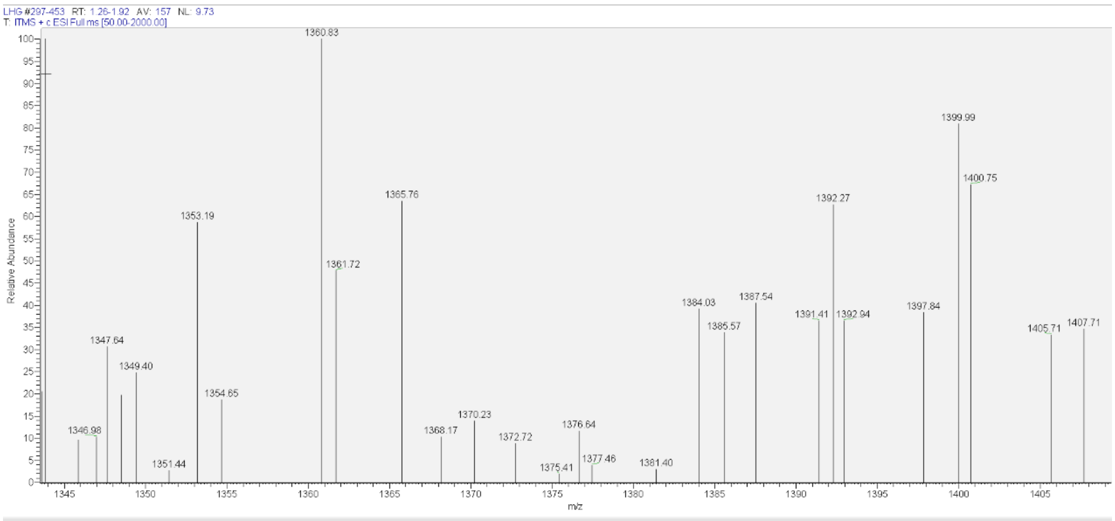
LHG #297-453 RT: 1.26-1.92 AV: 157 NL: 9.73
T: ITMS + c ESI Full ms [50.00-2000.00]
Relative Abundance
m/z

^{1}H NMR、^{13}C NMR and MS spectra of compound **12**

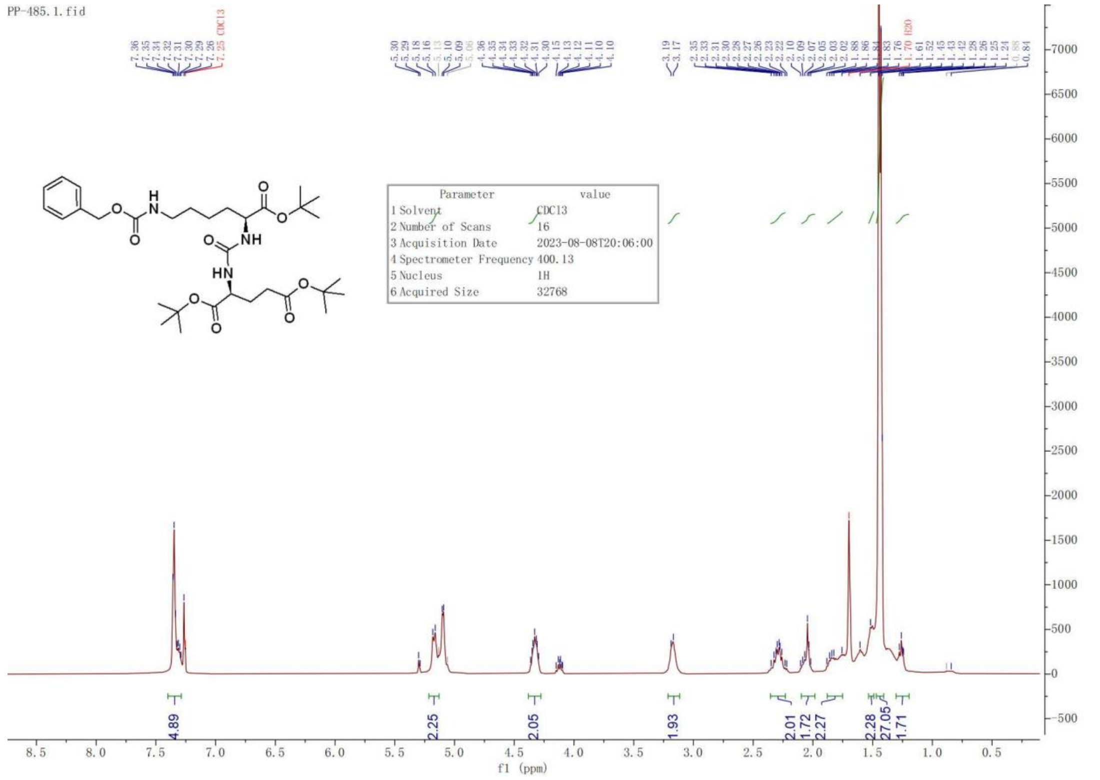

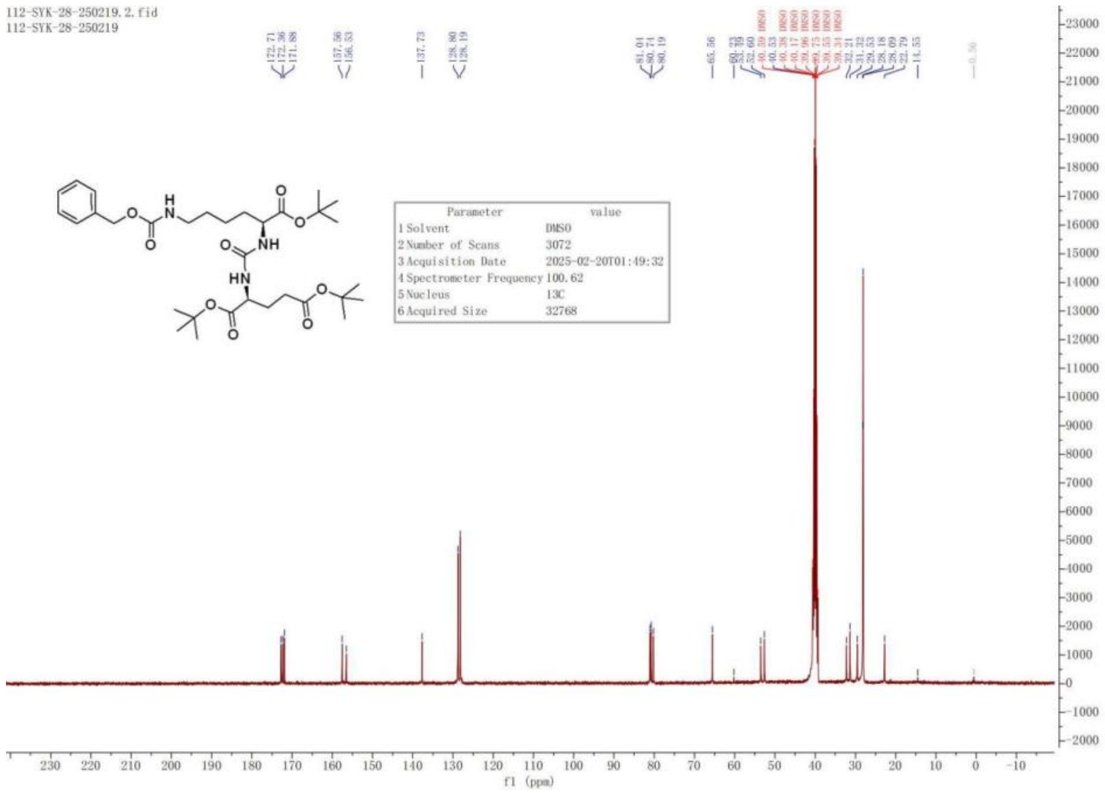

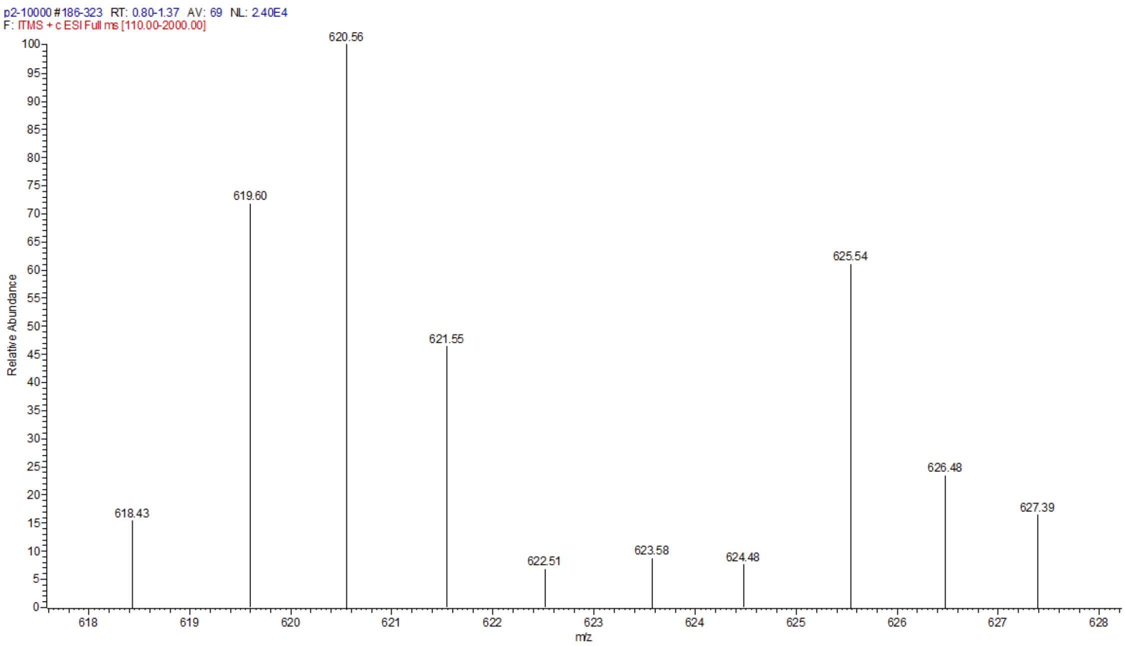

^{1}H NMR、^{13}C NMR and MS spectra of compound **13**

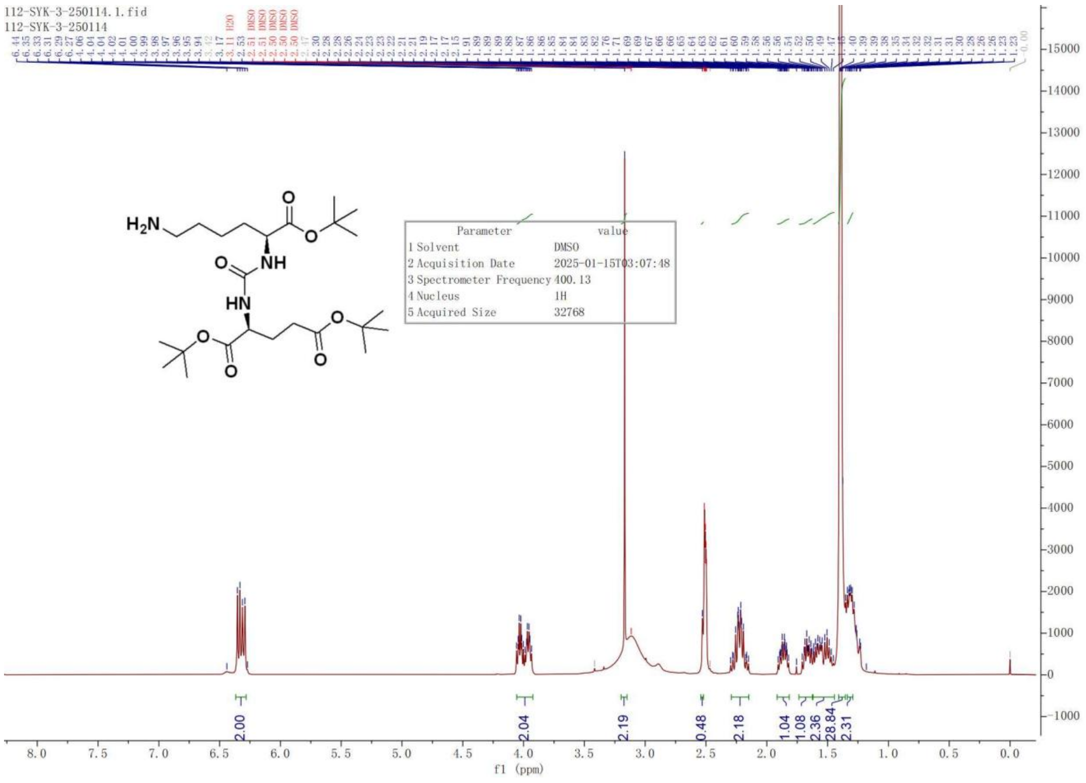

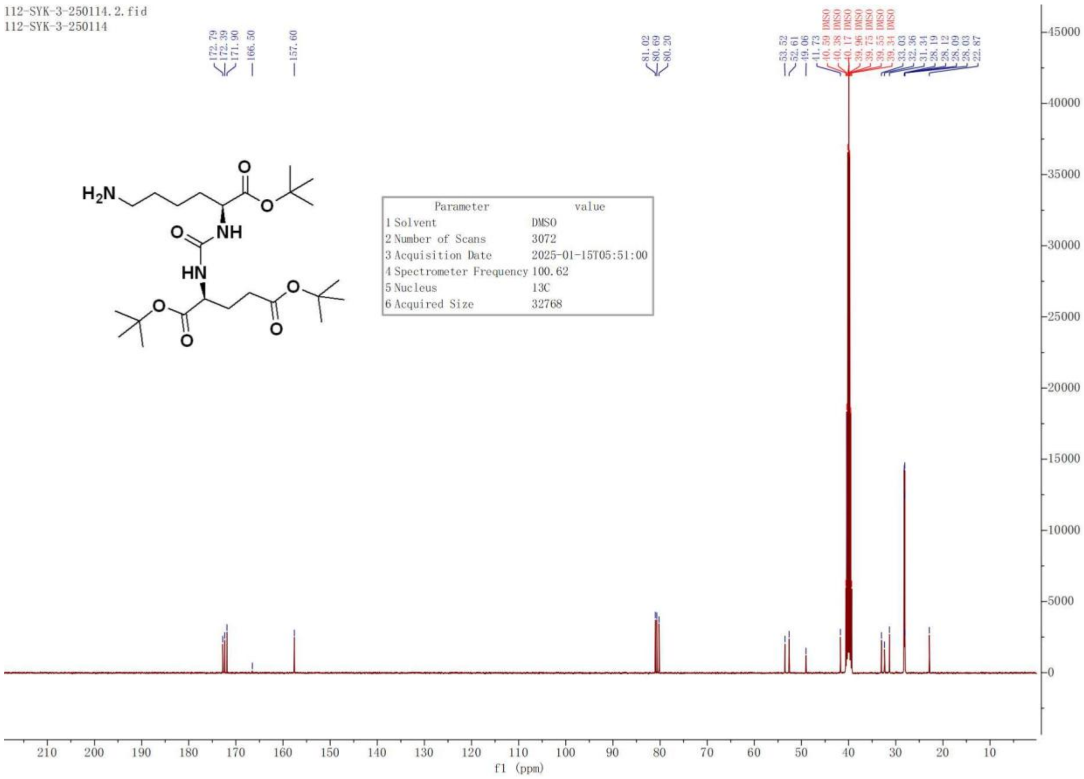

112-SYK-3-250114.2.fid
112-SYK-3-250114
172.79
172.39
171.90
166.50
157.60
81.02
80.69
80.20
53.52
52.61
49.06
41.73
40.59 DMSO
40.38 DMSO
40.17 DMSO
39.96 DMSO
39.75 DMSO
39.55 DMSO
39.34 DMSO
33.03
32.36
31.34
28.19
28.12
28.09
28.03
22.87
H2N
O
NH
HN
O
Parameter value
1 Solvent DMSO
2 Number of Scans 3072
3 Acquisition Date 2025-01-15T05:51:00
4 Spectrometer Frequency 100.62
5 Nucleus 13C
6 Acquired Size 32768
f1 (ppm)

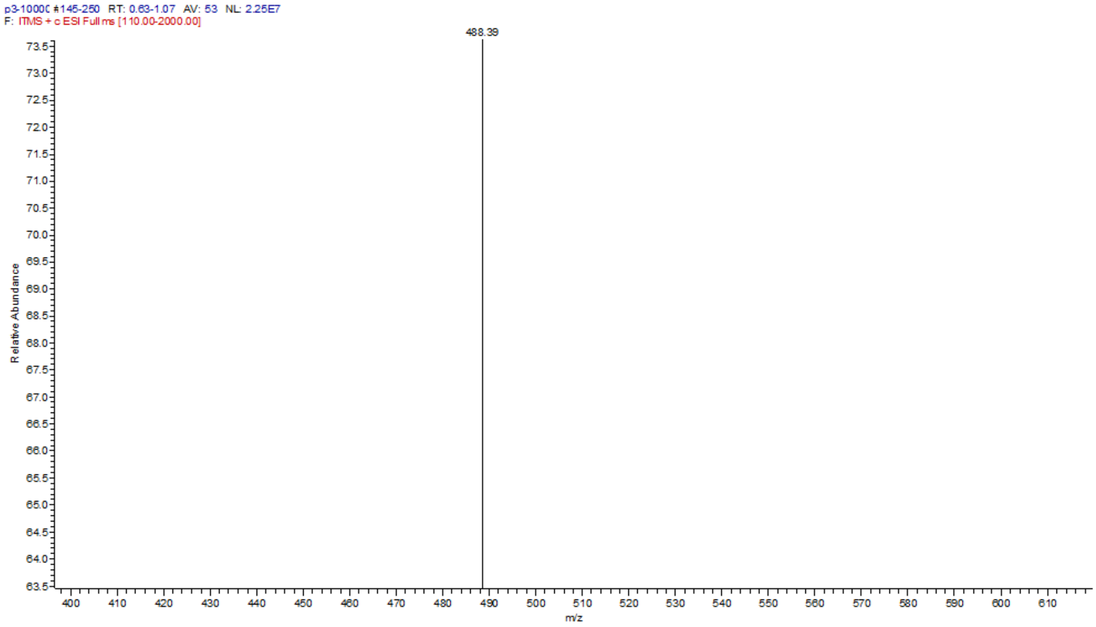

p3-10000 #145-250 RT: 0.63-1.07 AV: 53 NL: 2.25E7
F: ITMS + c ESI Full ms [110.00-2000.00]
488.39
Relative Abundance
m/z

^{1}H NMR、^{13}C NMR and MS spectra of compound **14**

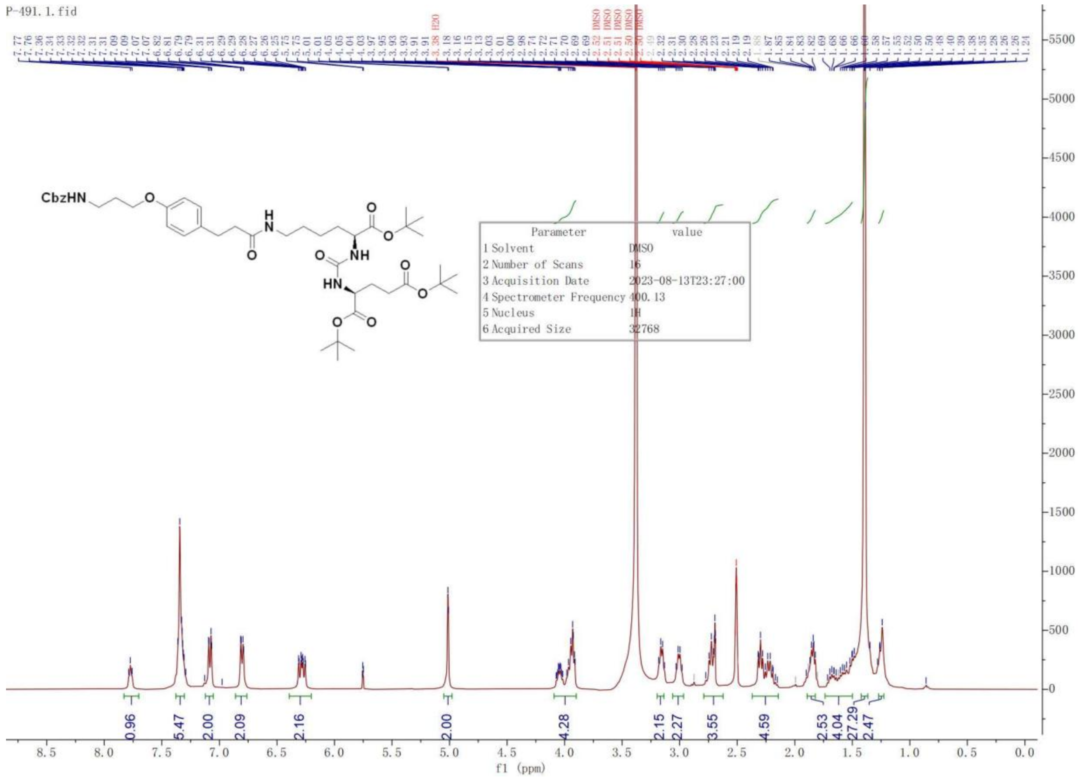

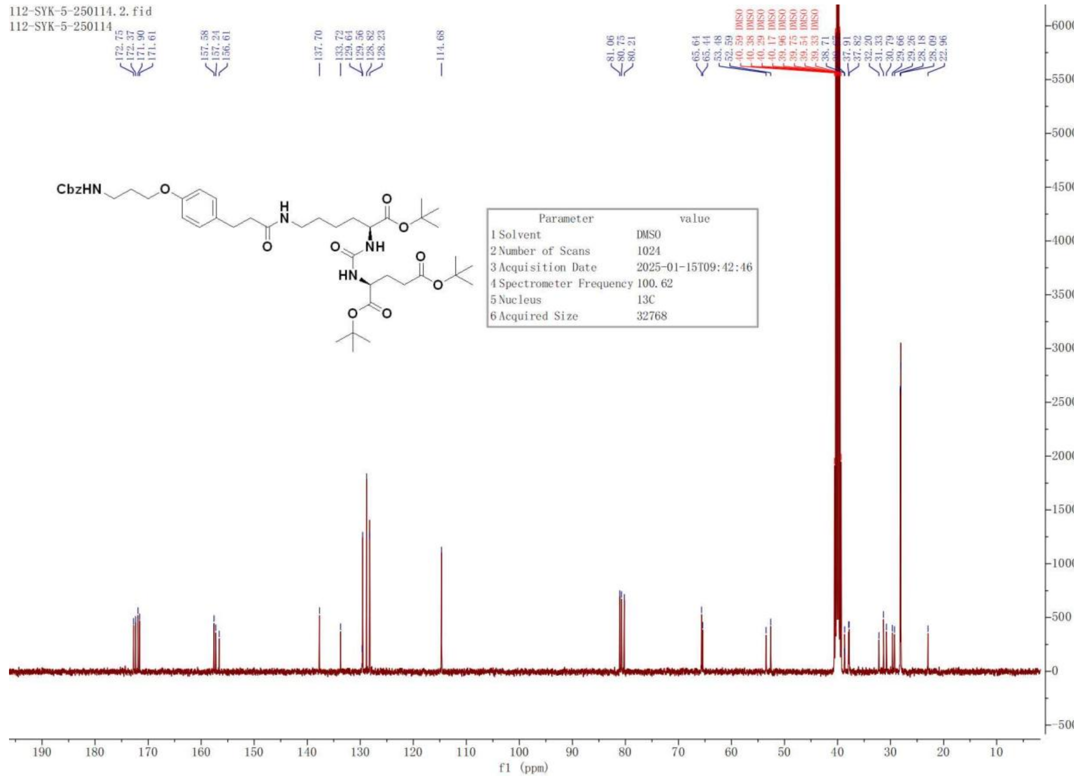

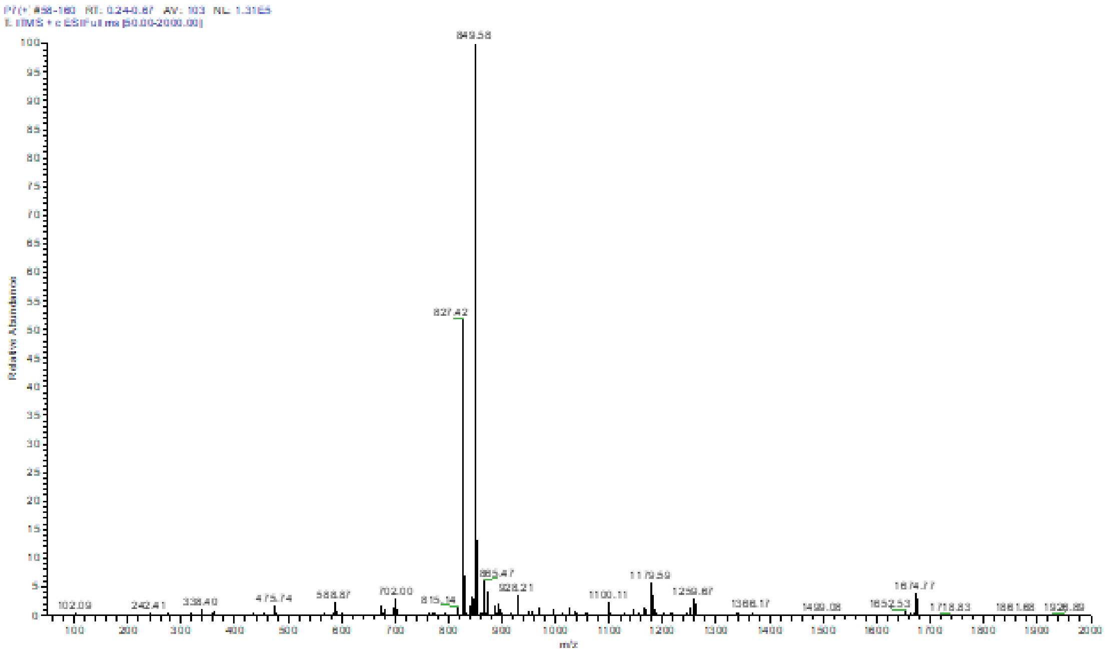

^{1}H NMR、^{13}C NMR and MS spectra of compound **15**

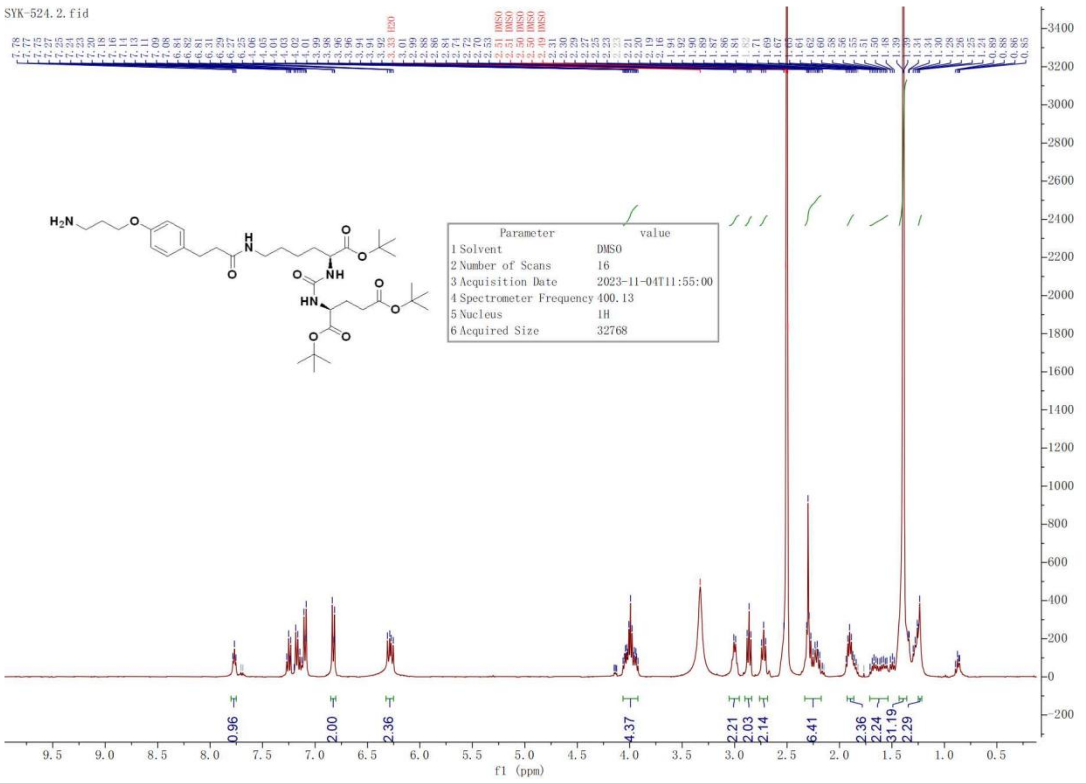

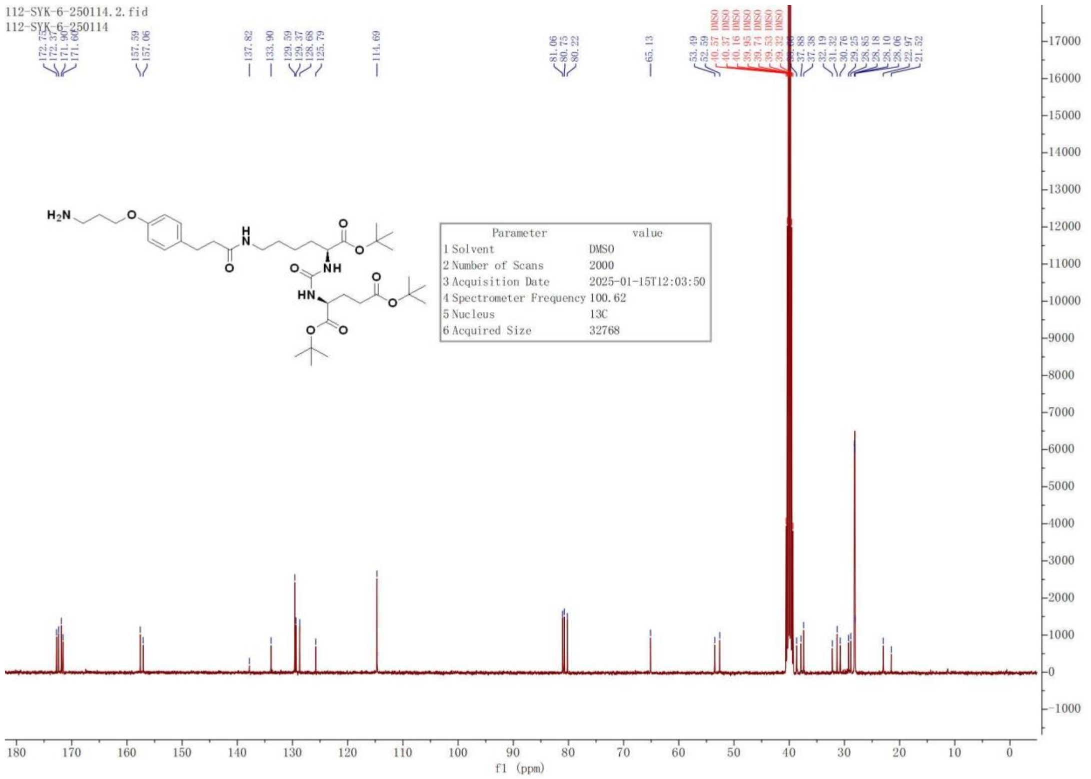

112-SYK-6-250114.2.fid
112-SYK-6-250114
Parameter value
1 Solvent DMSO
2 Number of Scans 2000
3 Acquisition Date 2025-01-15T12:03:50
4 Spectrometer Frequency 100.62
5 Nucleus 13C
6 Acquired Size 32768
f1 (ppm)

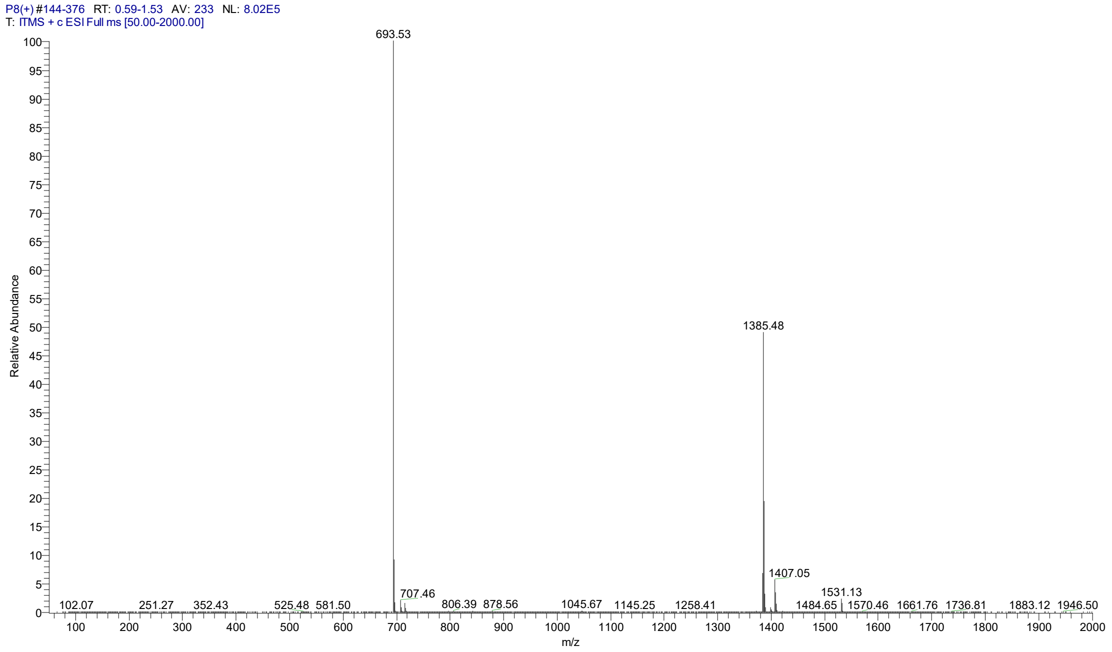

P8(+) #144-376 RT: 0.59-1.53 AV: 233 NL: 8.02E5
T: ITMS + c ESI Full ms [50.00-2000.00]
693.53
1385.48
1407.05
Relative Abundance
m/z

^{1}H NMR、^{13}C NMR and MS spectra of compound **16**

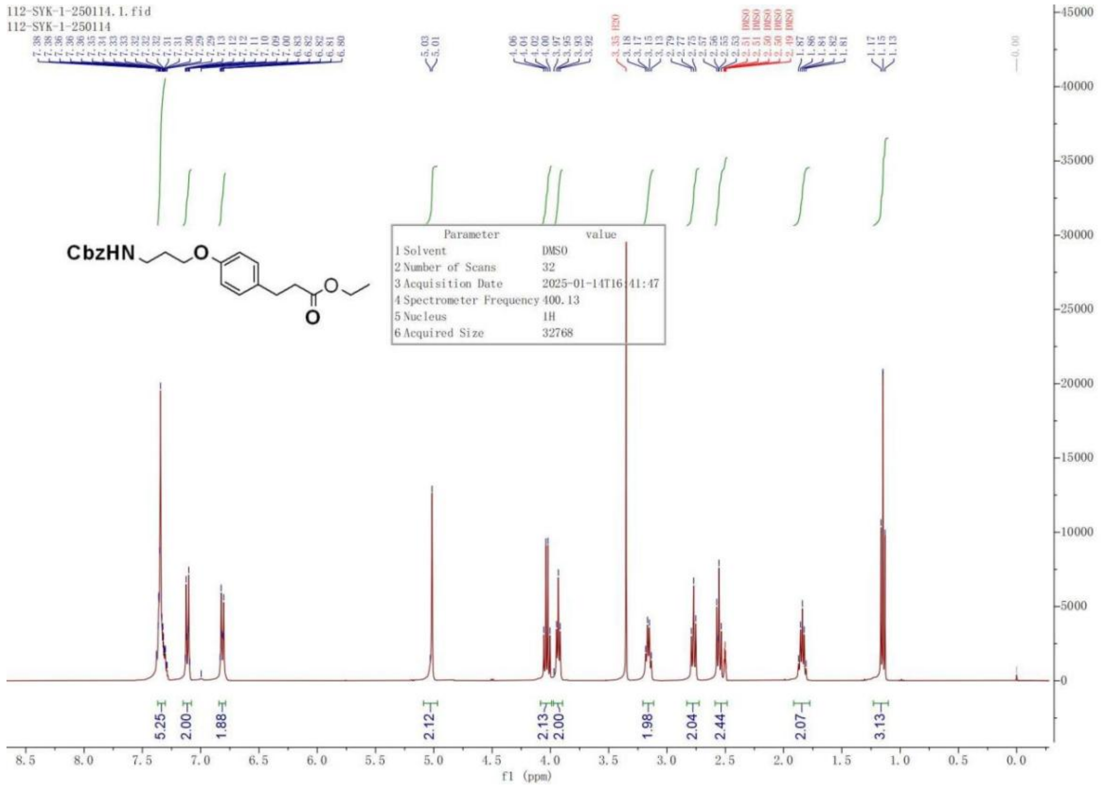

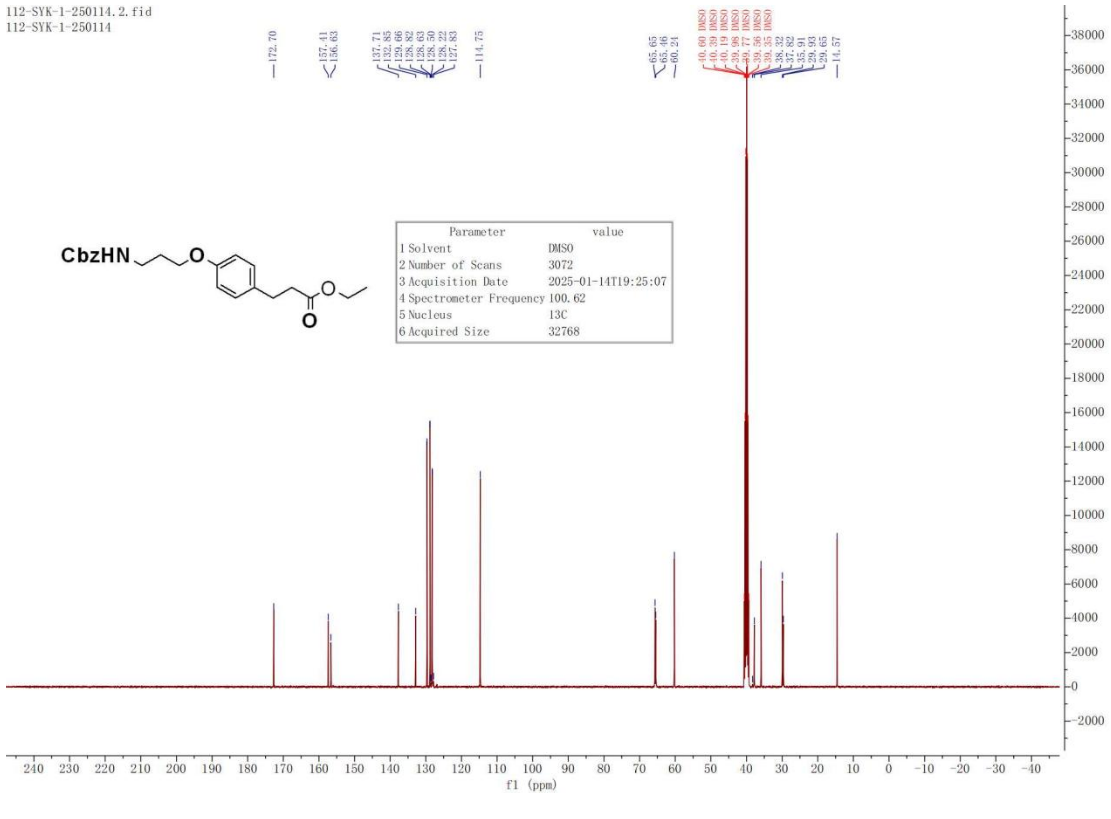

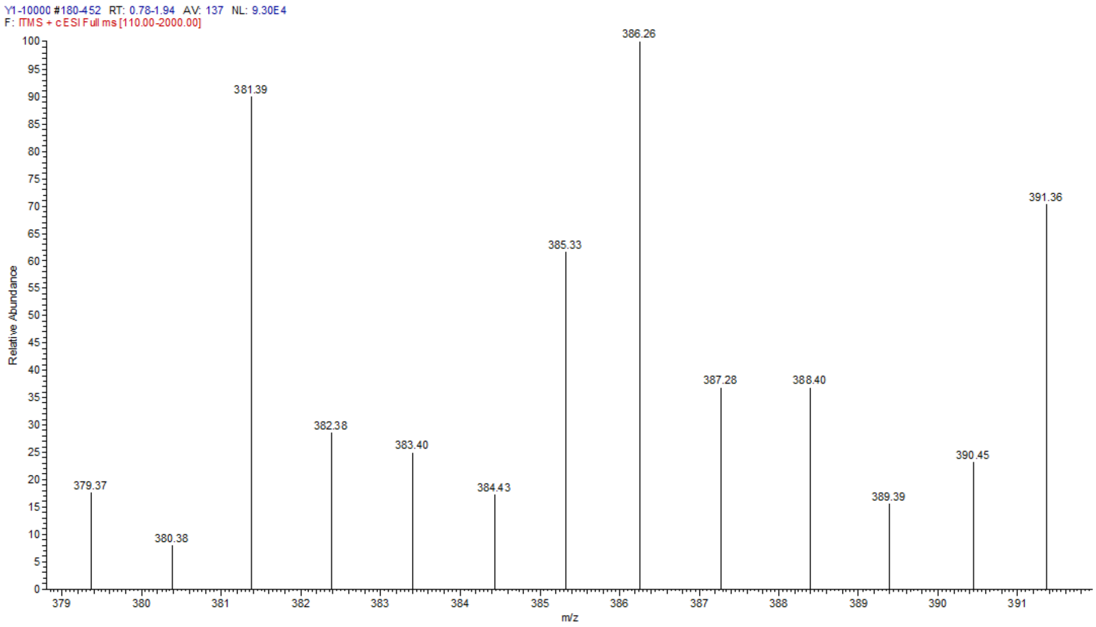

^{1}H NMR、^{13}C NMR and MS spectra of compound **17**

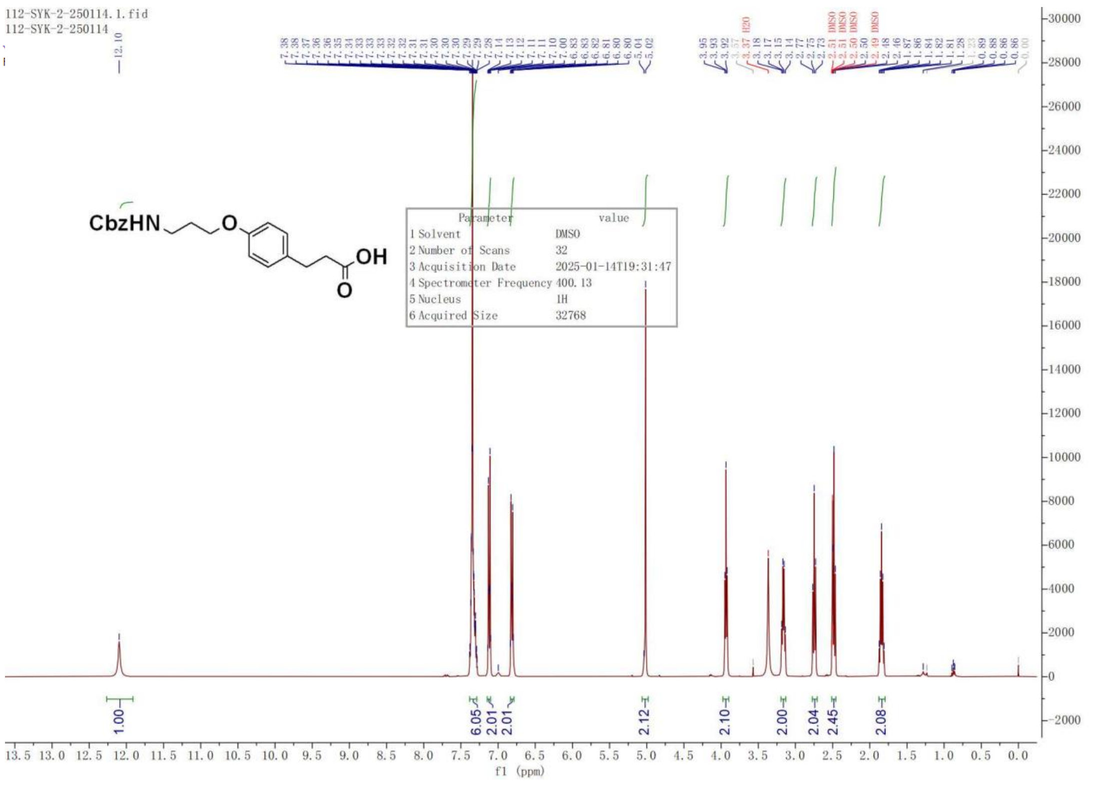

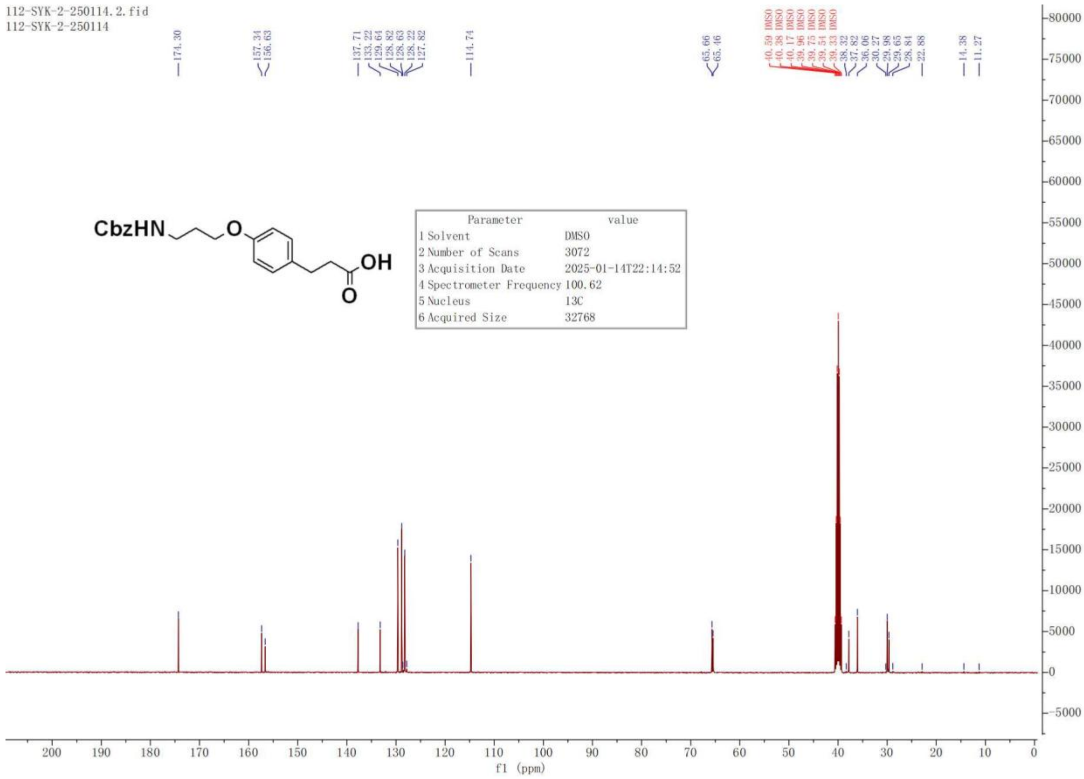
112-SYK-2-250114.2.fid
112-SYK-2-250114
174.30
157.34
156.63
137.71
133.22
129.64
128.82
128.63
128.22
127.82
114.74
65.66
65.46
40.59 DMSO
40.38 DMSO
40.17 DMSO
39.96 DMSO
39.75 DMSO
39.54 DMSO
39.33 DMSO
38.32
37.82
36.06
30.27
29.98
29.65
28.84
22.88
14.38
11.27
CbzHN
O
OH
O
Parameter value
1 Solvent DMSO
2 Number of Scans 3072
3 Acquisition Date 2025-01-14T22:14:52
4 Spectrometer Frequency 100.62
5 Nucleus 13C
6 Acquired Size 32768
f1 (ppm)

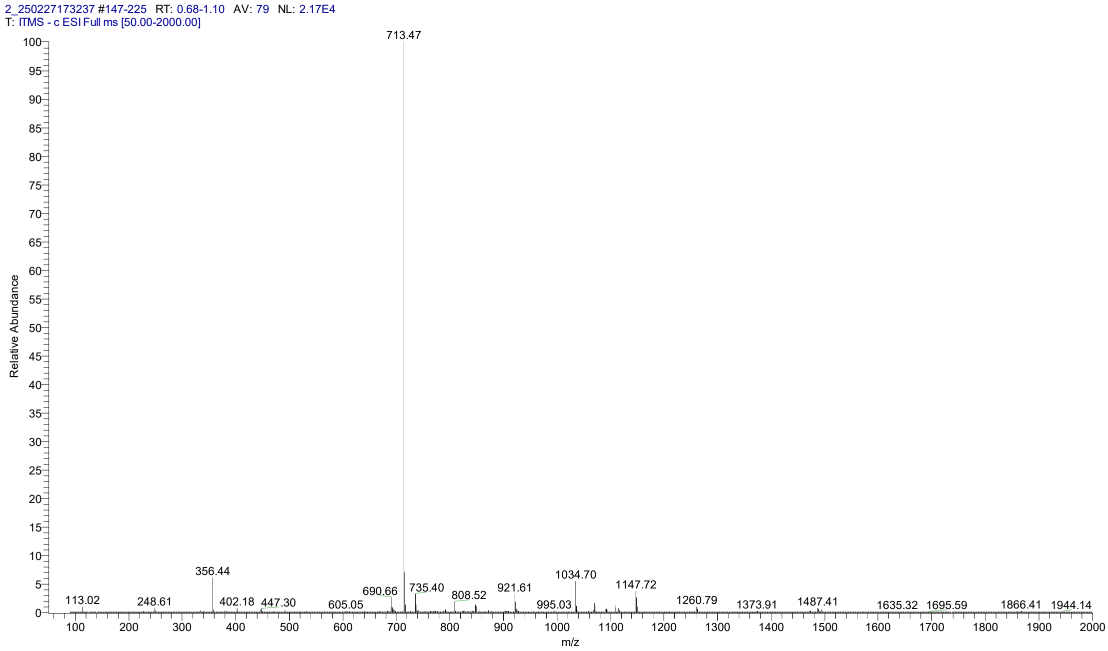
2_250227173237 #147-225 RT: 0.68-1.10 AV: 79 NL: 2.17E4
T: ITMS - c ESI Full ms [50.00-2000.00]
713.47
356.44
113.02
248.61
402.18
447.30
605.05
690.66
735.40
808.52
921.61
995.03
1034.70
1147.72
1260.79
1373.91
1487.41
1635.32
1695.59
1866.41
1944.14
Relative Abundance
m/z

^{1}H NMR、^{13}C NMR and MS spectra of compound **19**

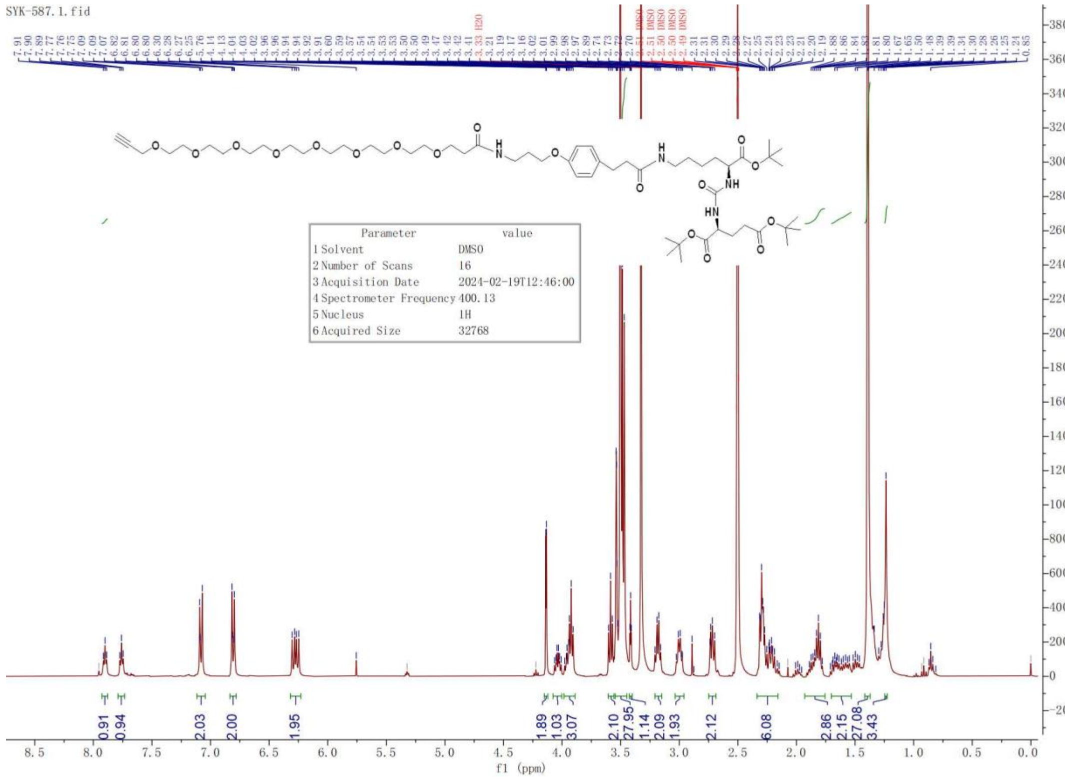

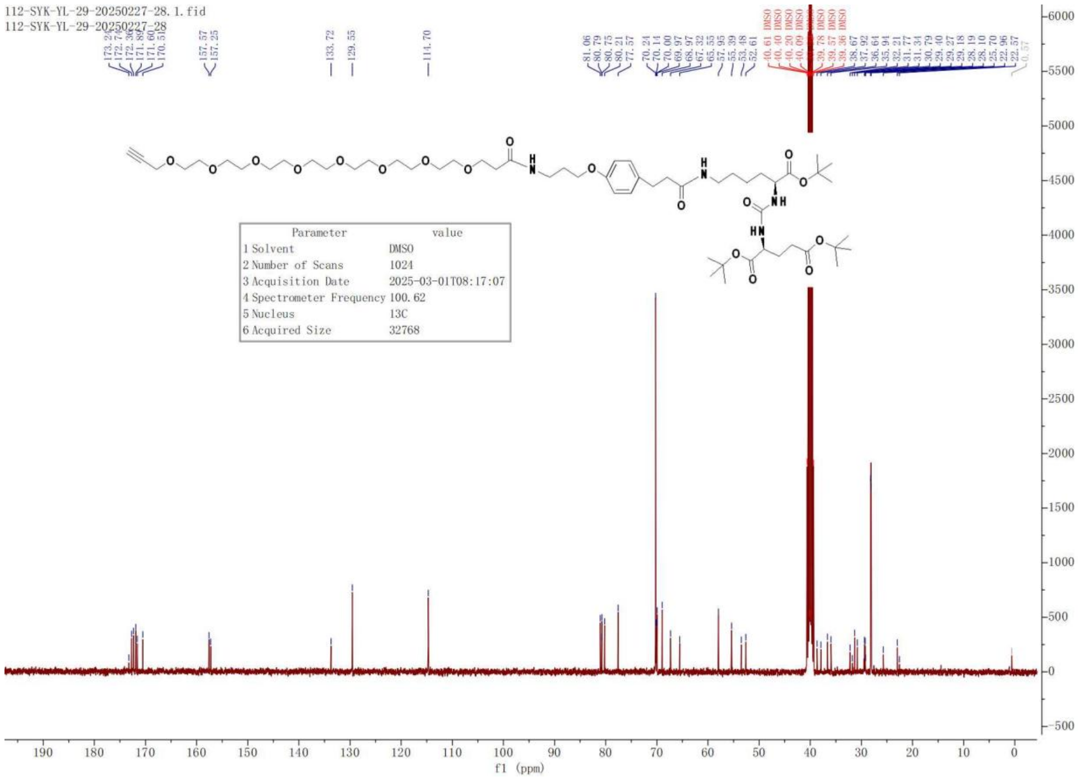

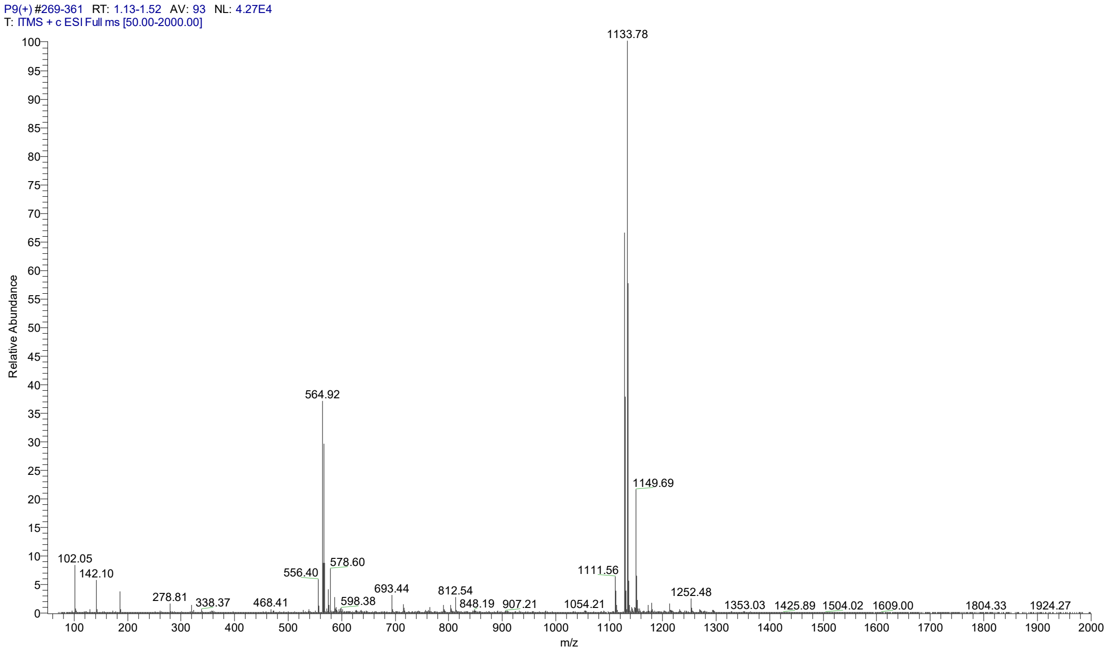

^{1}H NMR、^{13}C NMR and MS spectra of compound **EuK-linker B (20)**

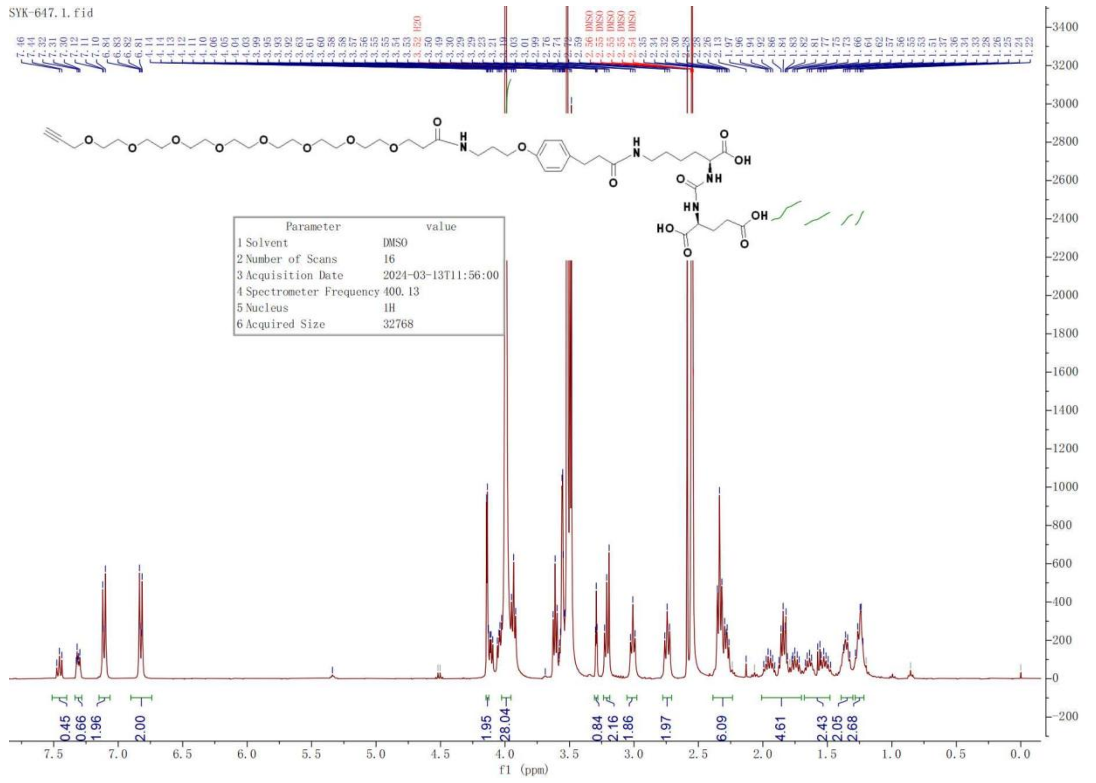

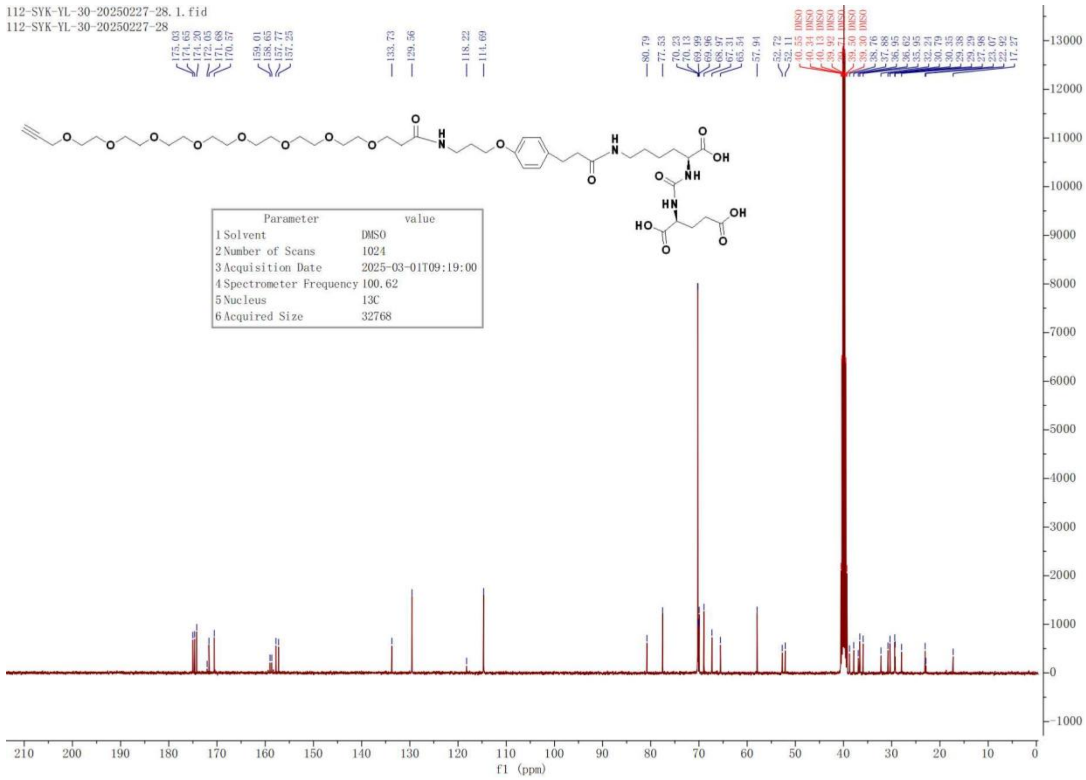

112-SYK-YL-30-20250227-28.1.fid
112-SYK-YL-30-20250227-28
175.03 174.65 174.20 172.05 171.68 170.57 159.01 158.65 157.77 157.25 133.73 129.56 118.22 114.69 80.79 77.53 70.23 70.13 69.99 69.96 68.97 67.31 65.54 57.94 52.72 52.11
40.55 DMSO 40.34 DMSO 40.13 DMSO 39.92 DMSO 39.71 DMSO 39.50 DMSO 39.30 DMSO
38.76 37.88 36.95 36.62 35.95 32.21 30.79 30.35 29.38 29.29 27.98 23.07 22.92 17.27
Parameter | value
1 Solvent | DMSO
2 Number of Scans | 1024
3 Acquisition Date | 2025-03-01T09:19:00
4 Spectrometer Frequency | 100.62
5 Nucleus | 13C
6 Acquired Size | 32768
f1 (ppm)

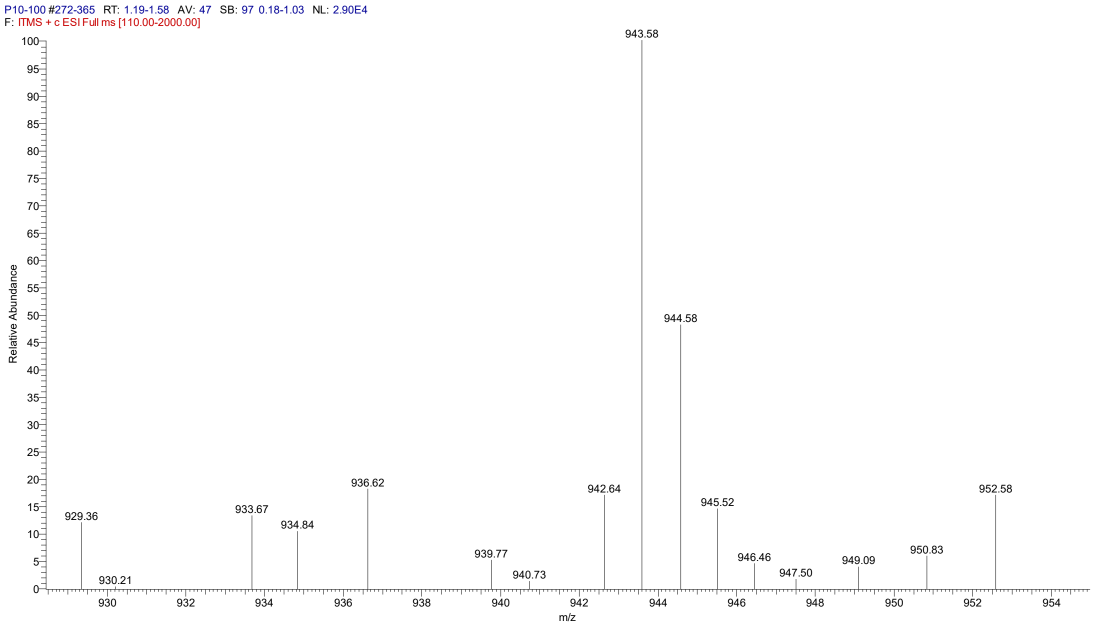

P10-100 #272-365 RT: 1.19-1.58 AV: 47 SB: 97 0.18-1.03 NL: 2.90E4
F: ITMS + c ESI Full ms [110.00-2000.00]
943.58
944.58
929.36
930.21
933.67
934.84
936.62
939.77
940.73
942.64
945.52
946.46
947.50
949.09
950.83
952.58
Relative Abundance
m/z

^{1}H NMR、^{13}C NMR and MS spectra of compound **21**

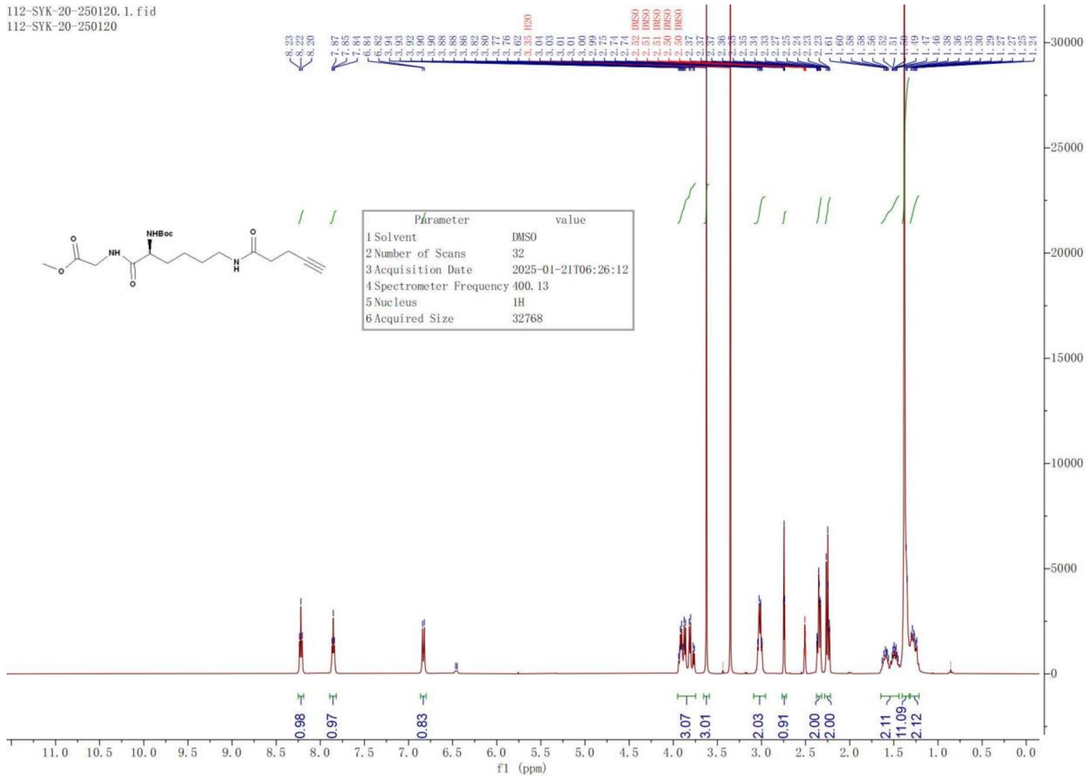

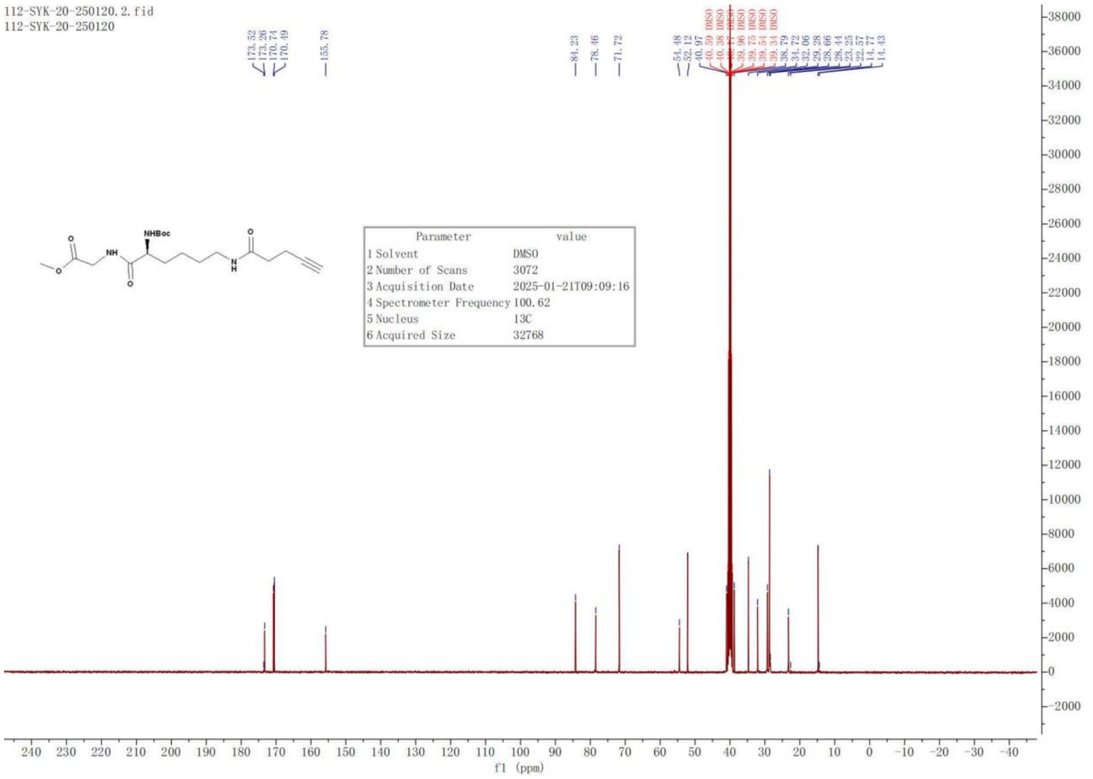

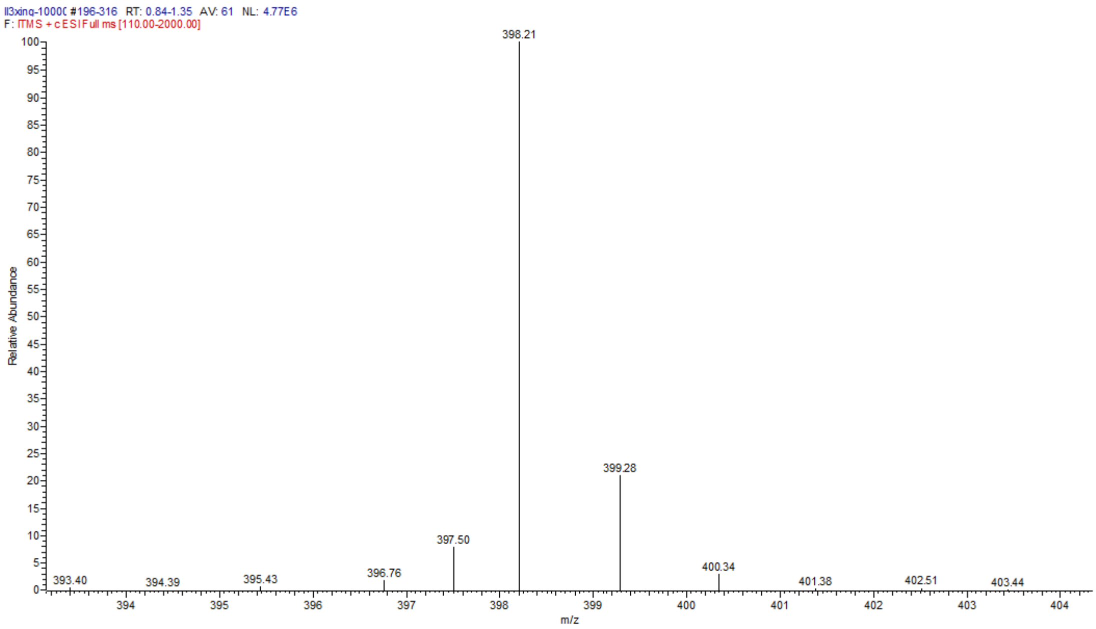

^{1}H NMR、^{13}C NMR and MS spectra of compound **22**

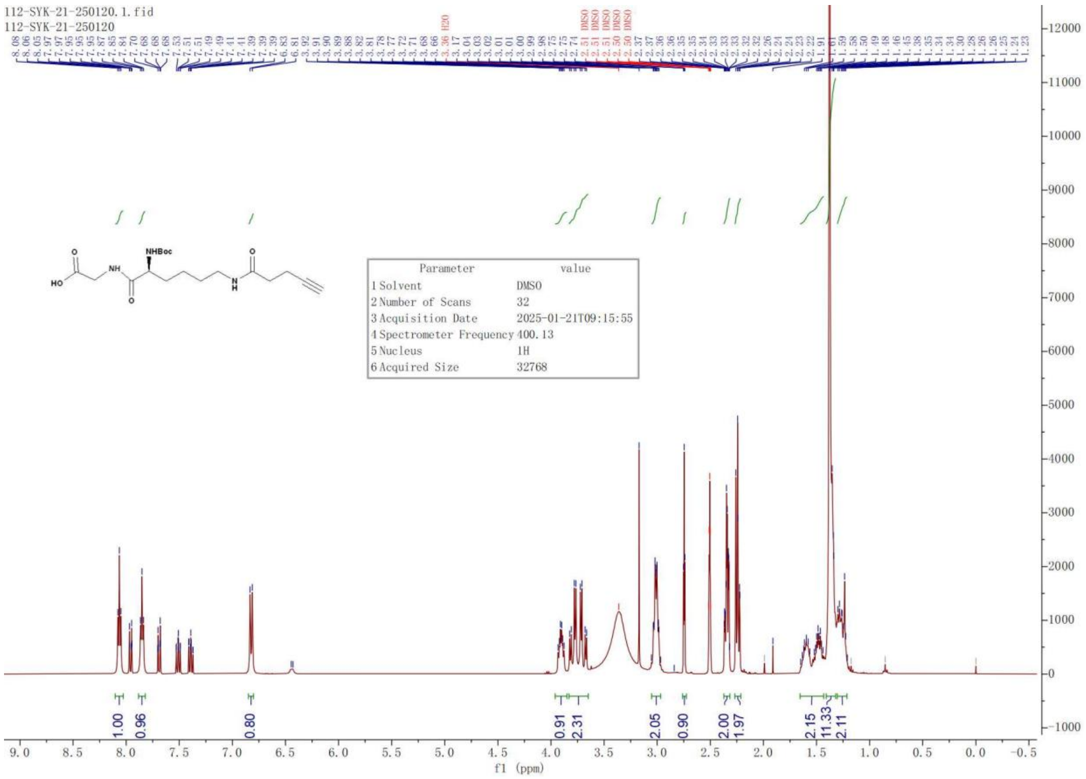

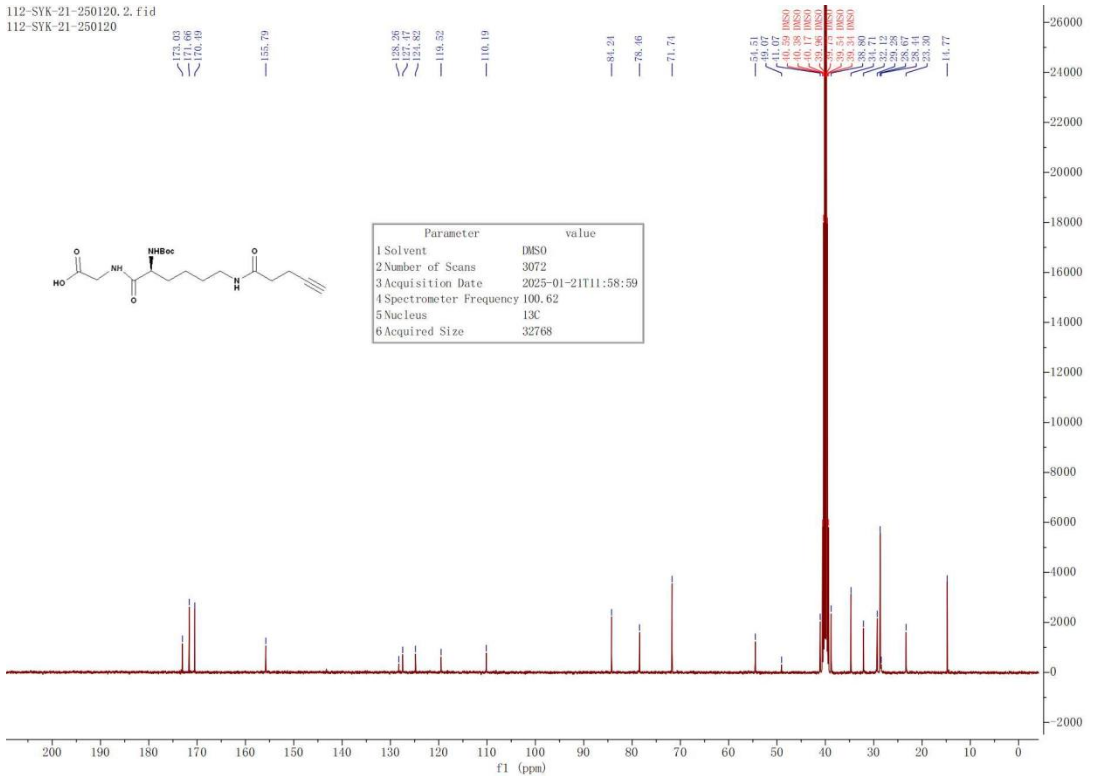

112-SYK-21-250120.2.fid
112-SYK-21-250120
173.03
171.66
170.49
155.79
128.26
127.47
124.82
119.52
110.19
84.24
78.46
71.74
54.51
49.07
41.07
40.59 DMSO
40.38 DMSO
40.17 DMSO
39.96 DMSO
39.75 DMSO
39.54 DMSO
39.34 DMSO
38.80
34.71
32.12
29.28
28.67
28.44
23.30
14.77
NHBoc
HO
NH
N
H
O
Parameter value
1 Solvent DMSO
2 Number of Scans 3072
3 Acquisition Date 2025-01-21T11:58:59
4 Spectrometer Frequency 100.62
5 Nucleus 13C
6 Acquired Size 32768
f1 (ppm)

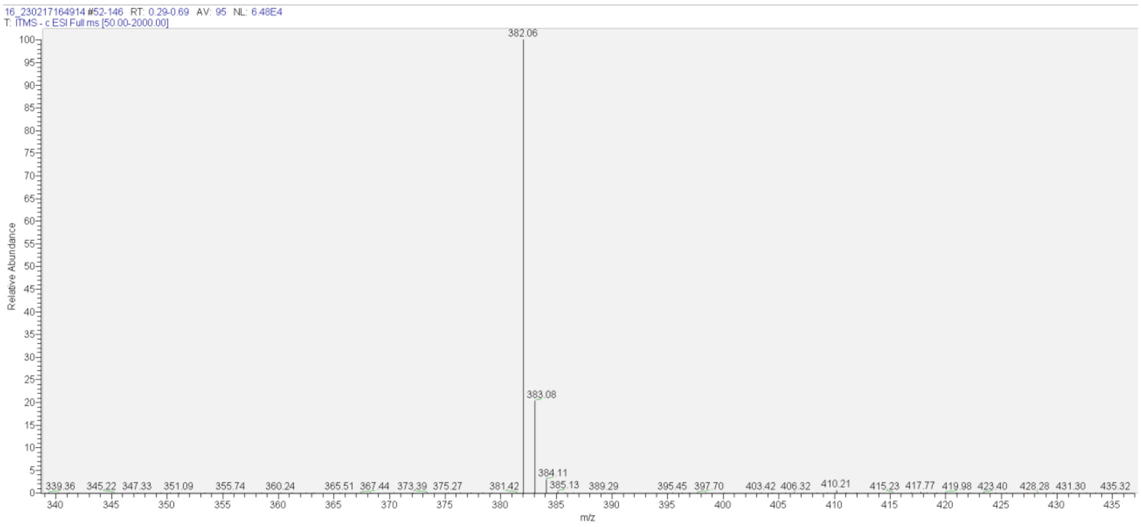

16_230217164914 #52-146 RT: 0.29-0.69 AV: 95 NL: 6.48E4
T: ITMS - c ESI Full ms [50.00-2000.00]
382.06
383.08
384.11
385.13
Relative Abundance
m/z

^{1}H NMR、^{13}C NMR and MS spectra of compound **CTX-linker C (23)**

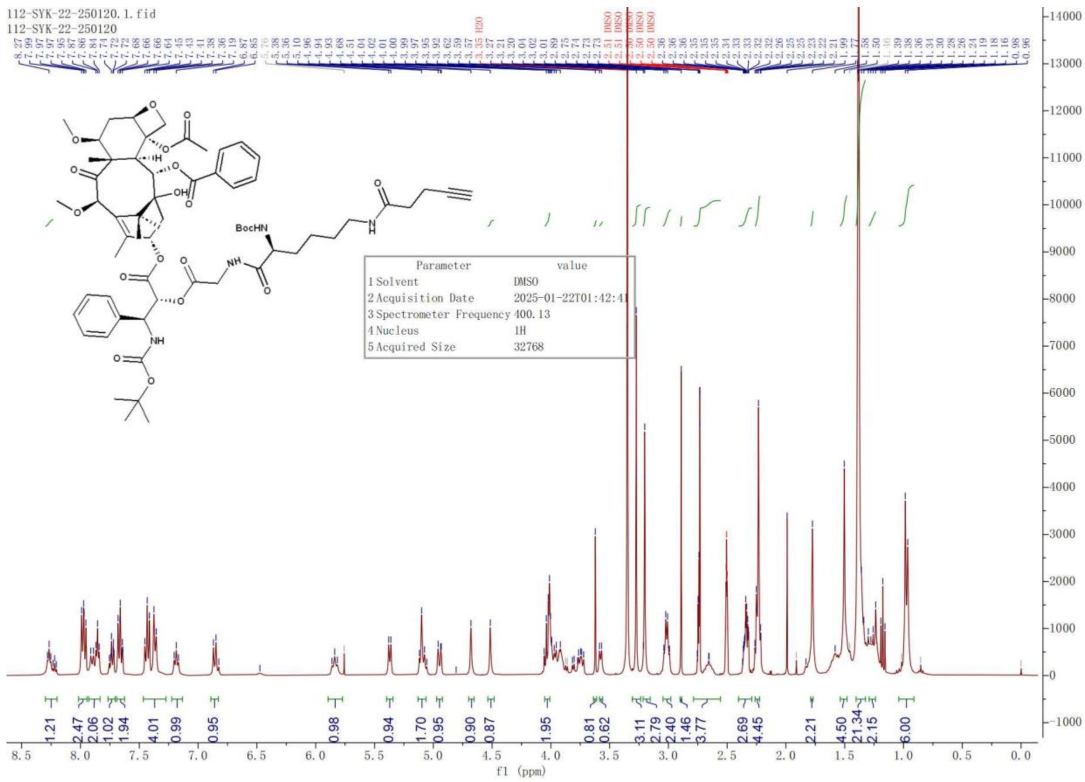

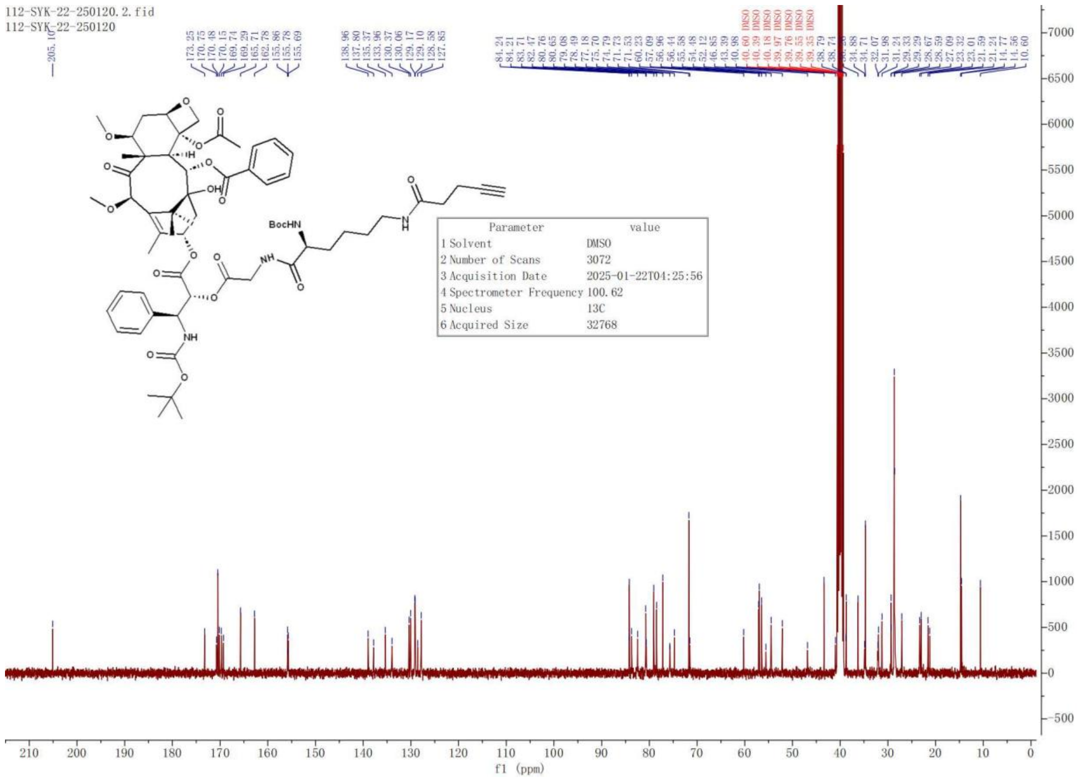

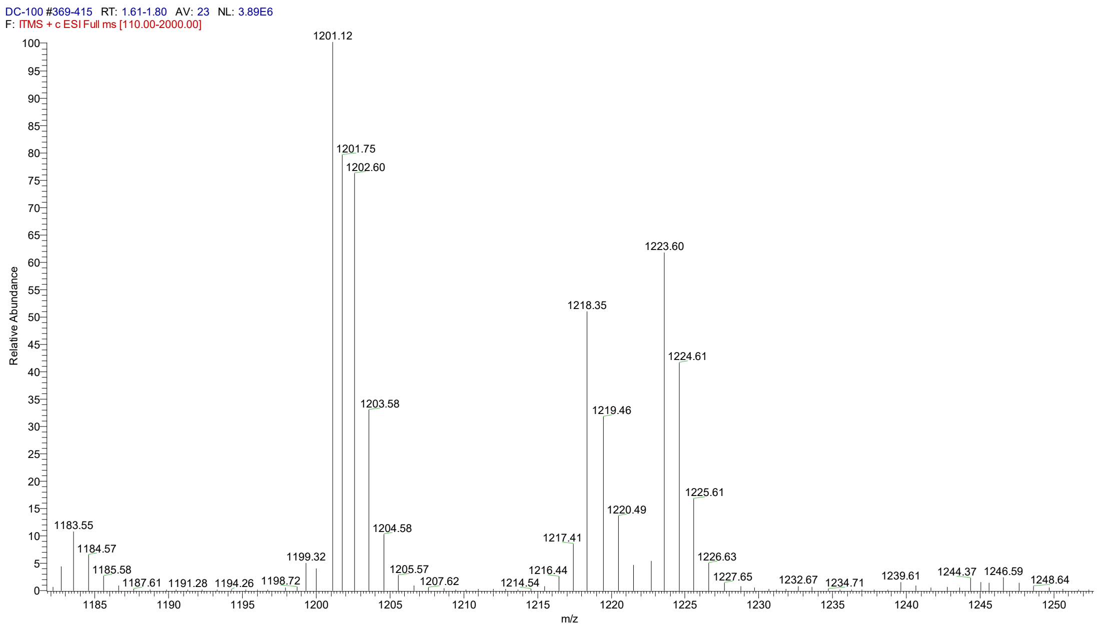

^{1}H NMR spectra of compound **Dextran-CTX-GLA-EuK (24)**

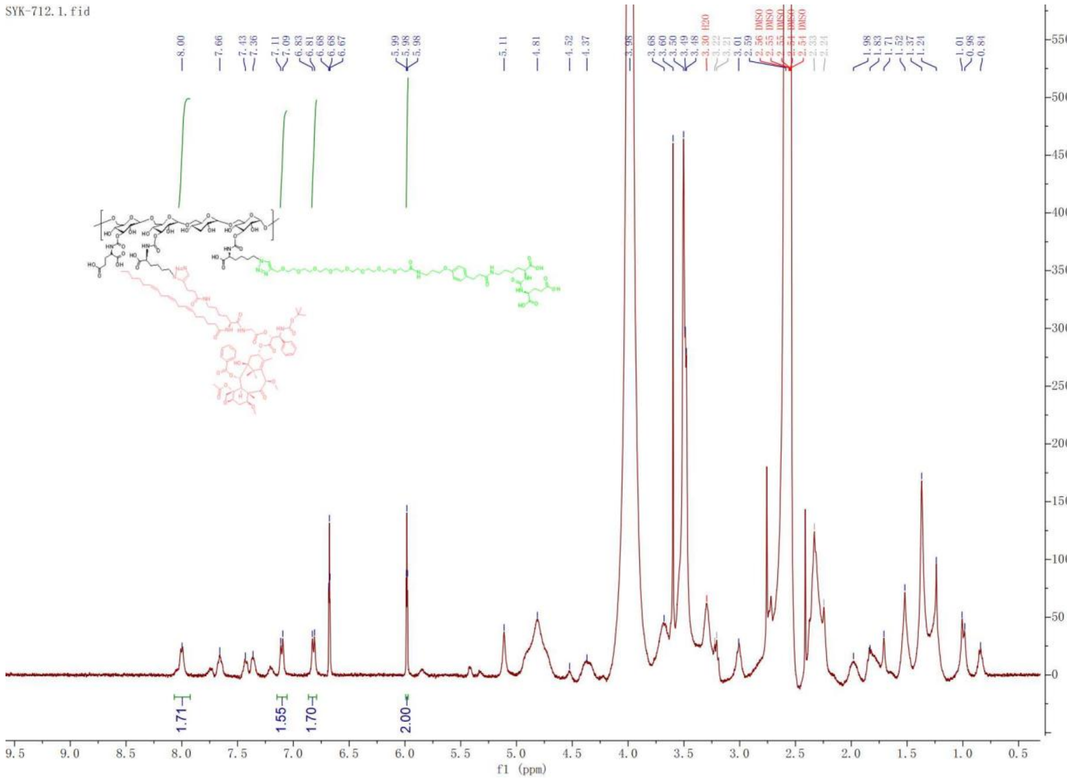

^{1}H NMR spectra of compound **Dextran-Cy7.5-EuK (25)**

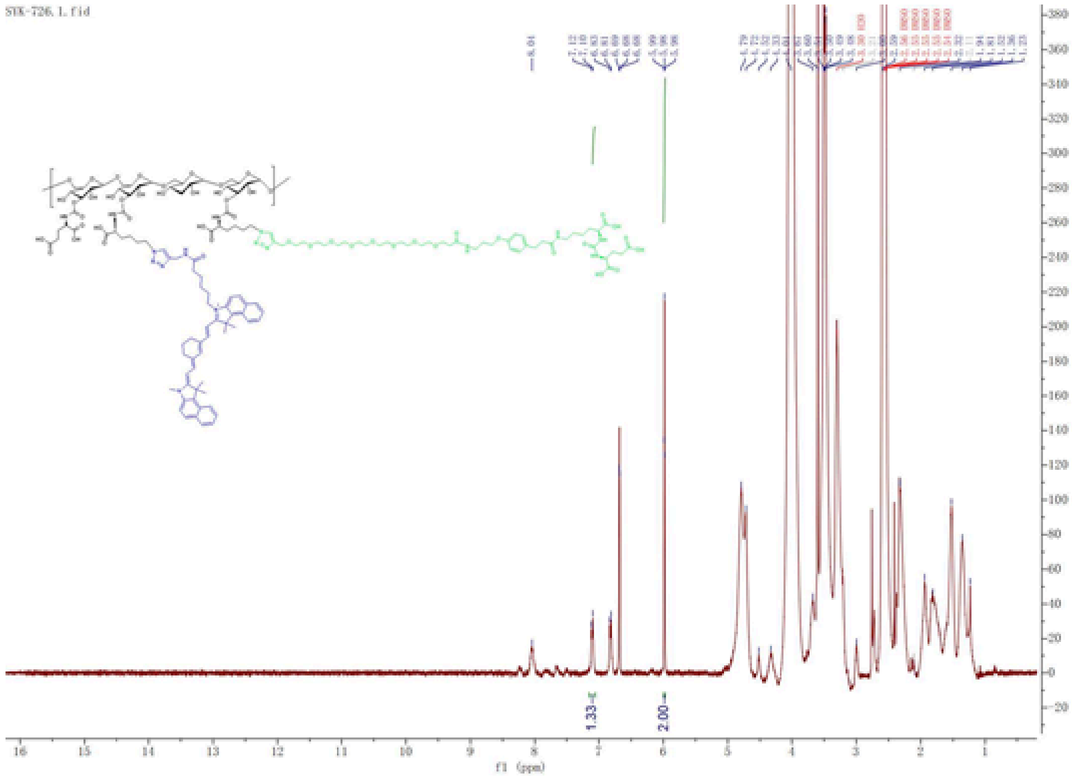

Supplement: Supplementary file 1 — Supplementary methods, figures. [file thnov16p6843s1.pdf]
